# A trehalase-derived MAMP triggers LecRK-V–mediated immune responses in *Arabidopsis*

**DOI:** 10.1126/sciadv.adv8896

**Published:** 2025-07-30

**Authors:** Erika Iino, Yasuhiro Kadota, Noriko Maki, Kazuki Sato, Erika Ono, Nobuaki Ishihama, Mason Rugen-Hankey, Siyuan Wei, Bruno Pok Man Ngou, Naoyoshi Kumakura, Marc W. Schmid, Sebastian Eves-van den Akker, Takamasa Suzuki, Taketo Uehara, Ken Shirasu

**Affiliations:** ^1^ RIKEN Center for Sustainable Resource Science (CSRS), Suehiro-cho 1-7-22 Tsurumi-ku, Yokohama, Kanagawa, 230-0045, Japan.; ^2^ Graduate school of Science, The University of Tokyo, 7-3-1, Hongo, Bunkyo-ku, Tokyo, 113-8654, Japan.; ^3^ The Crop Science Centre, Department of Plant Sciences, University of Cambridge, Cambridge, UK.; ^4^ MWSchmid GmbH, Glarus, Switzerland.; ^5^ College of Bioscience and Biotechnology, Chubu University, Kasugai, 487-0027, Japan.; ^6^ Institute for Plant Protection, National Agriculture and Food Research Organization, Tsukuba, Japan.

## Abstract

Plant-parasitic nematodes (PPNs) cause major agricultural losses worldwide, yet the molecular basis of plant immunity against these pathogens remains poorly understood. To investigate how plants recognize PPNs, we aimed to identify microbe-associated molecular patterns (MAMPs) from nematodes and the corresponding plant immune components. Because of the limited availability of material from obligate PPNs, we used *Caenorhabditis elegans*, a free-living nematode, as a MAMP source. *C. elegans* extract activated MAMP-triggered immune responses in *Arabidopsis* Col-0. Through chromatography-based purification, we identified a secreted trehalase and pinpointed a conserved peptide region essential for its MAMP activity. A corresponding peptide from root-knot nematode trehalase enabled the identification of lectin receptor kinases LecRK-V.5 and LecRK-V.6 as key components in immune induction. Notably, this peptide region is conserved across some phytophagous insects and fungal pathogens, with LecRK-Vs required for immune responses to these peptides, highlighting the role of LecRK-V–mediated mechanism for broad-spectrum pathogen detection via trehalase-derived peptides.

## Teaser

Trehalase-derived peptides trigger LecRK-V-mediated plant immunity, potentially enabling broad detection of pathogenic organisms.

## INTRODUCTION

Plant-parasitic nematodes (PPNs) rank among the most destructive agricultural pests, causing an estimated annual global crop loss of ~175 billion USD (*1*, *2*). The most economically damaging PPNs are sedentary endoparasites, including root-knot nematodes (RKNs) and cyst nematodes (CNs) (*1*). RKNs exhibit a broad host range, infecting thousands of plant species (*1*), while CNs, athough more selective, target important crops such as potatoes and soybeans (*1*). Upon root penetration, these endoparasites secrete a suite of effectors from their subventral gland cells to the plant apoplast to facilitate tissue migration (*3*). Notably, CNs migrate intracellularly, while RKNs move intercellularly. Once reaching the vascular tissue, these nematodes secrete effectors from the dorsal gland cell, reprogramming plant cells into specialized feeding structures, such as giant cells or syncytia, which sustain them throughout their sedentary stage (*4*).

Plants detect microbes through the perception of microbe-associated molecular patterns (MAMPs) via surface-localized pattern recognition receptors (PRRs), leading to the activation of MAMP-triggered immunity (MTI) (*5*). A well-characterized example of a MAMP is flg22, a peptide derived from flagellin originally identified in the plant pathogenic *Pseudomonas* (*6*), which is recognized by the PRR FLS2 (*7*). Another notable MAMP is elf18, a peptide from bacterial elongation factor Tu (EF-Tu), isolated from nonpathogenic *Escherichia coli* (*8*) and perceived by the PRR EFR (EF-TU RECEPTOR) (*9*). Similarly, *N*-acetylchitooligosaccharides (chitin), fungal MAMPs (*10*), are detected by the PRR CERK1 (CHITIN ELICITOR RECEPTOR KINASE 1). (*11*). The activation of these PRRs triggers a series of downstream immune responses, including calcium influx (*12*, *13*) and reactive oxygen species (ROS) bursts (*14*, *15*), cell wall fortification through lignin and callose deposition, activation of mitogen-activated protein kinases (MAPKs), phytoalexin biosynthesis, and the induction of defense-related genes (*16*). Because nematodes maintain close physical contact with plant cells during both migratory and sedentary stages, MTI likely plays a crucial role in plant defense against these parasites (*17*). Thus, understanding the molecular basis of nematode recognition, likely via their MAMPs, is essential for advancing our knowledge of plant immunity against PPNs.

Previous studies have identified the conserved nematode pheromone ascaroside (ascr#18) as the first nematode-derived MAMP (*18*). Ascr#18 was shown to be recognized by the PRR NILR1 (NEMATODE-INDUCED LEUCINE-RICH REPEAT RECEPTOR-LIKE KINASE 1), which was originally identified to be involved in MTI induction in response to nematode-derived compound (*19*–*21*). Although this is the only currently proposed MAMP-PRR pair in nematode-plant interactions, many more MAMPs and their corresponding PRRs are likely to remain undiscovered. Finding previously unknown MAMP-PRR pairs in nematode-plant interactions will be key to understanding how plants defend themselves against these persistent agricultural pests.

In this study, we describe the identification of a novel MAMP peptide derived from secreted trehalase in nematodes using a biochemical approach. Using the natural variation in *Arabidopsis thaliana*, we successfully mapped the locus responsible for immune responses triggered by the MAMP peptide. This locus contains a gene cluster encoding L-type lectin receptor kinases (LecRKs), and knockout analysis confirmed that *LecRK-V.5* and *LecRK-V.6* as key components required for the MAMP-inducible immune responses. Intriguingly, the MAMP sequence was conserved not only in nematodes but also across a range of other plant pathogens, suggesting that secreted trehalase may be a common target for plant immune recognition. This conservation highlights the significance of trehalase, a trehalose-degrading enzyme, as a crucial factor for diverse pathogens using distinct infection strategies. These findings expand our understanding of MAMP-triggered plant immunity and suggest broad implications for developing resistance strategies against multiple pathogens.

## RESULTS

### A screen for MAMPs in nematode extract

**Fig. 1.**
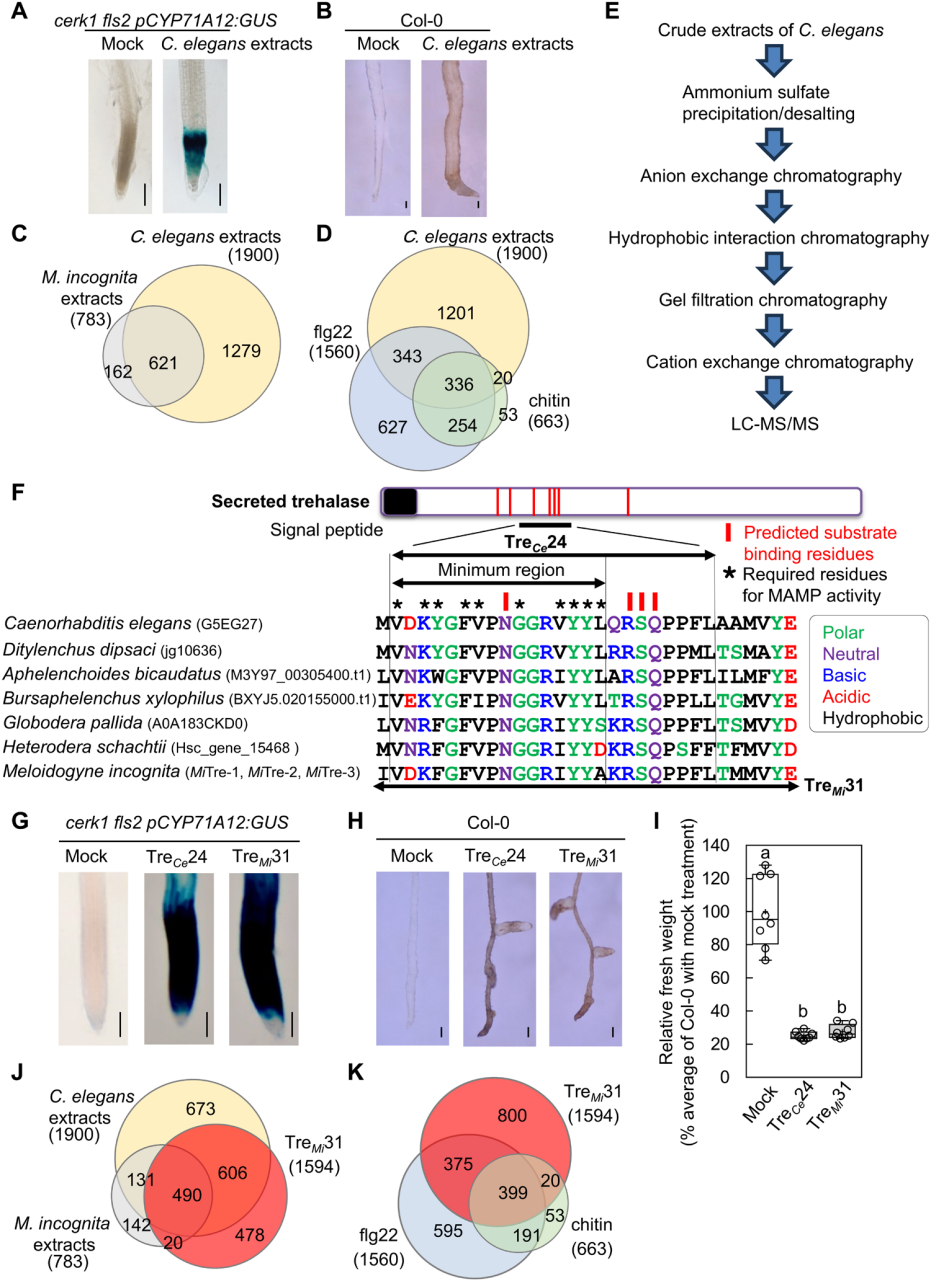
Purification and characterization of MAMPs from *C. elegans*. (**A** and **B**) *C. elegans* extract (100 μg/ml) triggers MAMP-inducible responses in *Arabidopsis* Col-0, including *CYP71A12* expression (A) and root pigmentation (B). *CYP71A12* expression was assessed using the *cerk1 fls2 pCYP71A12:GUS* line. Scale bars, 100 μm. Each treatment included four seedlings, and experiments were repeated at least three times with consistent results. (**C**) Venn diagram showing genes up-regulated [log_2_fold change (log_2_FC) ≥ 1, adjusted *P* ≤ 0.01] in *efr fls2 cerk1* triple mutants treated with *C. elegans* and *M. incognita* extract (100 μg/ml, 12 hours). (**D**) Genes up-regulated by *C. elegans* extract substantially overlap with those induced by flg22 (*25*) and chitin (*26*). (**E**) Workflow for MAMP identification from *C. elegans* extract via chromatography and LC-MS/MS. (**F**) Sequence alignment of trehalase-derived peptides from *C. elegans* and plant-parasitic nematodes (PPNs). Red lines mark predicted substrate-binding residues; asterisks indicate essential residues for MAMP activity (see fig. S9). Amino acids are color-coded by chemical properties. Secreted trehalase gene models in *M. incognita* were manually refined to correct annotation errors (figs. S6 to S8). (**G** to **I**) 50 μM Tre*_Ce_*24 and Tre*_Mi_*31 peptides induce *CYP71A12* expression (G), root pigmentation (H), and seedling growth inhibition (I) in Col-0. Scale bars, 100 μm. Box plots show median (line), interquartile range (box), range (whiskers), and mean (cross); individual points represent eight biological replicates. Different letters indicate significant differences [*P* ≤ 0.00001, one-way analysis of variance (ANOVA) with Tukey’s test]. Each treatment included four seedlings, and experiments were repeated at least three times with consistent results. (**J**) Venn diagram showing genes up-regulated (log_2_FC ≥ 1, adjusted *P* ≤ 0.01) in Col-0 treated with Tre*_Mi_*31 (50 μM, 12 hours), overlapping with genes induced by *C. elegans* and *M. incognita* extract. (**K**) Tre*_Mi_*31-induced genes substantially overlap with those induced by flg22 (*25*) and chitin (*26*).

To elucidate the molecular mechanisms underlying plant recognition of nematodes, we sought to identify MAMPs from PPNs and corresponding plant genes that are required for MAMP-triggered immune responses. However, the obligate parasitism of PPNs and a life cycle of several weeks present significant technical challenges in obtaining sufficient material for the biochemical identification of MAMPs. To overcome this, we used the free-living nematode *Caenorhabditis elegans*, leveraging the conserved nature of MAMPs among pathogenic and nonpathogenic organisms. Using *Arabidopsis* expressing the *GUS* reporter system driven by the promoter of the immunity marker gene *CYP71A12* (*pCYP71A12:GUS*) (*22*), we explored responses triggered by *C. elegans* extract. To exclude potential contamination of bacterial MAMPs, we used a *cerk1 fls2 pCYP71A12:GUS* line. Notably, the treatment with *C. elegans* extract for 24 hours induced *GUS* expression in the root elongation zone (Fig. 1A), similar to the responses triggered by the MAMP flg22 and *Meloidogyne incognita* extract and its infection (*22*, *23*). In addition, we observed root pigmentation (Fig. 1B) and lignin accumulation (fig. S1), both hallmarks of the immune responses in resistant plants under nematode attack (*17*, *24*). RNA sequencing (RNA-seq) analysis revealed that *C. elegans* extract up-regulated a significant number of genes in the *efr fls2 cerk1* triple PRR mutant, many of which were also induced by *M. incognita* extract (Fig. 1C and tables S1 and S2), suggesting a shared response. Gene Ontology (GO) enrichment analysis showed that these up-regulated genes were mostly associated with immunity-related pathways (table S3). In addition, *C. elegans* extract induced over half of the genes activated by flg22 (*25*) or chitin (*26*), highlighting transcriptional overlaps among these treatments (Fig. 1D and table S4). These findings demonstrate the existence of potential nematode MAMP(s) in *C. elegans* extract.

### MAMP purification from *C. elegans*

For biochemical identification of MAMP(s), we propagated ~1 kg of *C. elegans* in liquid culture and used 50 to 100 g for each purification. MAMP activity at each purification step was assessed using the *cerk1 fls2 pCYP71A12:GUS* line. The activity was initially detected in the 40 to 70% ammonium sulfate precipitate (Fig. 1E and fig. S2). After desalting, the potential MAMP(s) was purified through a series of chromatography techniques, including anion exchange, hydrophobic interaction, gel filtration, and cation exchange chromatography. Chromatographic purifications were repeated multiple times to optimize conditions and assess reproducibility. Active fractions from the final cation exchange step were analyzed by liquid chromatography tandem mass spectrometry (LC-MS/MS). We conducted two independent purification experiments, consistently identifying the same set of 25 proteins (table S5). Of these, nine proteins were selected for further analysis based on the following criteria: (i) high conservation in PPNs; (ii) isoelectric point of 5.5 to 8.0, consistent with the migration pattern of *GUS*-inducing activity in ion exchange chromatography; and (iii) predicted molecular mass of 20 to 100 kDa, aligning with the behavior of *GUS*-inducing activity in gel filtration chromatography. On the basis of sequence conservation among PPNs, we synthesized 19 peptides from these nine candidate proteins to test their *GUS*-inducing activity (fig. S3). Of the 19 peptides, only one, a 24–amino acid peptide derived from secreted trehalase (designated as Tre*_Ce_*24; Fig. 1F), induced *GUS* expression in the *cerk1 fls2 pCYP71A12:GUS* line (Fig. 1G).

### Sequence specificity of trehalase-derived peptides

Trehalase is an enzyme that catalyzes the hydrolysis of trehalose, a disaccharide, into glucose molecules. *C. elegans* has five trehalase-encoding genes (*Ce*TRE1 to *Ce*TRE5; fig. S4). The peptide Tre*_Ce_*24 is derived from *Ce*TRE3, and multiple specific peptides from *Ce*TRE3 were identified through LC-MS/MS analysis (fig. S4 and table S6). Tre*_Ce_*24 contains predicted substrate-binding residues based on the structural data from yeast trehalase (*27*) and is highly conserved among PPNs (Fig. 1F and figs. S5 to S8). Peptides with longer C-terminal sequences (Tre*_Ce_*26 and Tre*_Ce_*32) demonstrated stronger activity, while those with shorter C-terminal sequences (Tre*_Ce_*16 and Tre*_Ce_*15) exhibited reduced or no *GUS* expression, indicating the importance of the glutamine (17th) and leucine (16th) residues (fig. S9A). Similarly, peptides with truncated N-terminal sequences (Tre*_Ce_*24-2, Tre*_Ce_*24-4, Tre*_Ce_*24-6, and Tre*_Ce_*24-8) also showed reduced activity (fig. S9B), highlighting the critical role of the N-terminal valine, aspartic acid, lysine, and tyrosine residues. These results establish that the sequence VDKYGFVPNGGRVYYL represents the minimal region required for MAMP activity, with peptides containing a longer C terminus exhibiting higher activity. Alanine scanning of the Tre*_Ce_*19 sequence further identified 10 essential residues for *GUS* expression, demonstrating clear sequence specificity (Fig. 1F and fig. S9C). In PPNs, secreted trehalases are released during infection to use host-derived trehalose. In pine wood nematodes, the secretion of trehalase increases in response to pine extract (*28*, *29*). In CNs, a secreted trehalase (encoded by *Hsc_gene_15468*) has been identified as a virulence effector candidate, produced in subventral gland cells, and is likely secreted into the apoplast during the early stages of infection (*30*–*32*). Notably, previous studies showed that *Hsc_gene_15468* transcripts accumulate significantly in gland cells during the migratory parasitic second-stage juvenile (J2) phase and that expression levels in this phase are ~14,000 times higher than the cyst phase and even 14 times higher than in the preparasitic J2 phase (fig. S10, A and B) (*30*–*32*). Similarly, in *M. incognita*, transcripts of secreted trehalase genes (*Mi*Tre-1 and *Mi*Tre-3; see Materials and Methods for refinement of these gene models) have been reported to be enriched in both the preparasitic J2 and parasitic J3 stages based on transcriptomic data from a previous study (fig. S10C) (*32*, *33*). Peptides derived from homologous secreted trehalases in several PPNs, including *Ditylenchus dipsaci* (stem and bulb nematode), *Aphelenchoides bicaudatus* (mycetophagous nematode), *Bursaphelenchus xylophilus* (pine wood nematode), *Globodera pallida* and *Heterodera schachtii* (CNs), and *M. incognita* and *Meloidogyne enterolobii* (RKNs), all exhibited *GUS* induction activity (Fig. 1G and figs. S5 and S11A). Because of the agricultural importance of *M. incognita*, a peptide derived from its trehalase (Tre*_Mi_*) was selected for further analysis. The shorter peptide (Tre*_Mi_*16: VDKFGFVPNGGRIYYA), corresponding to the minimal MAMP active region of Tre*_Ce_*, had weak activity, while the longer Tre*_Mi_*31 peptide induced a stronger immune response (fig. S11B), reflecting the sequence specificity observed in peptides from *C. elegans*. To investigate the spatial relationship of these peptides within the trehalase protein, we predicted the structures of *Ce*TRE-3 and *M. incognita* trehalase using AlphaFold3 (*34*). Comparison with the crystal structure of the yeast neutral trehalase Nth1 in complex with trehalose (Protein Data Bank: 5M4A) (*27*) revealed that the MAMP active peptide regions are located at or near the trehalose-binding site (fig. S12), potentially explaining their strong conservation across diverse nematodes.

### Tre*_Mi_*31 activates a series of MAMP responses

Next, we examined plant responses induced by Tre*_Mi_*31. Tre*_Mi_*31 induced root pigmentation and growth abnormalities, including root tip bending, lateral root formation, and inhibition of primary root growth, which became evident ~7 days after treatment. These phenotypes resemble those triggered by the phytocytokine SCOOP12 (*35*), which is recognized by the PRR MIK2 (MDIS1-INTERACTING RECEPTOR LIKE KINASE 2) (Fig. 1H) (*36*, *37*). In addition, Tre*_Mi_*31 induced lignin accumulation and inhibited seedling growth in a manner comparable to *C. elegans* extract, Tre*_Ce_* 24, known MAMPs (*38*), and nematode culture filtrates (*19*) (Fig. 1I and fig. S13). RNA-seq analysis revealed that Tre*_Mi_*31 up-regulated 1594 genes (table S7), including a significant portion of the genes induced by *C. elegans* and *M. incognita* extract (Fig. 1J). Furthermore, Tre*_Mi_*31 activated more than half of the genes up-regulated by flg22 (*25*) and chitin (*26*) (Fig. 1K). GO enrichment analysis showed that the up-regulated genes were predominantly associated with immunity, phytoalexin biosynthesis, and cell wall thickening, particularly lignin biosynthesis, while down-regulated genes were linked to root growth and development (table S8). In addition, Kyoto Encyclopedia of Genes and Genomes pathway analysis of genes differentially expressed in response to Tre*_Mi_*31, but not to flg22 (*39*), revealed the activation of pathways involved in phenylalanine, tyrosine, and tryptophan biosynthesis, as well as phenylpropanoid biosynthesis (fig. S14, A and B), consistent with the observed lignin accumulation (fig. S14C). Moreover, Tre*_Mi_*31 strongly activated genes such as the immunity marker *CYP71A12*, which is involved in the biosynthesis of tryptophan-derived secondary metabolites such as camalexin, indole glucosinolates, and 4-hydroxy indole-3-carbonyl nitrile (fig. S14D) (*40*).

### LecRK-V.5 and LecRK-V.6 are required for Tre*_Mi_*31 signaling

**Fig. 2.**
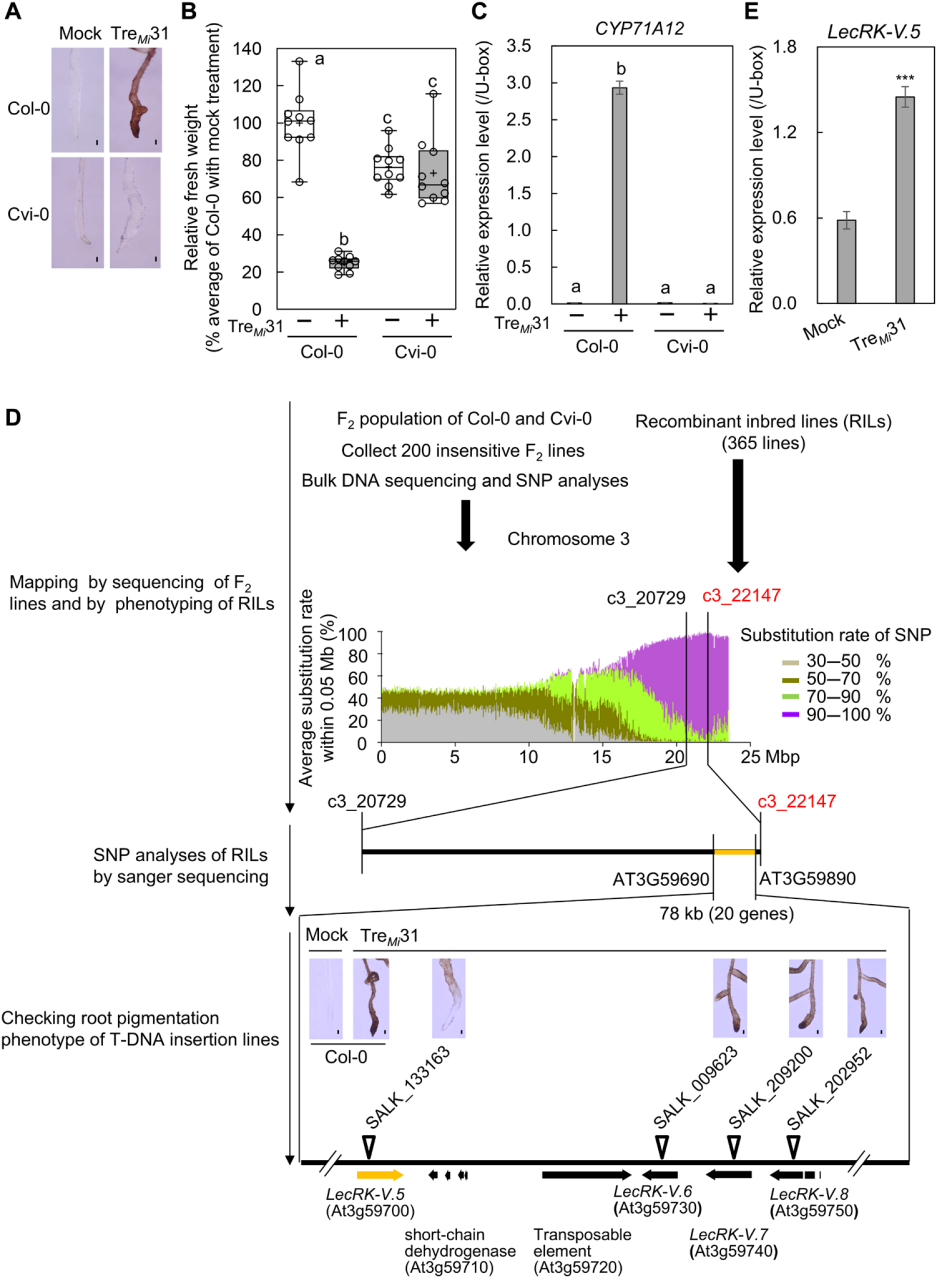
Identification of the genomic region conferring Tre*_Mi_*31 insensitivity in Cvi-0. (**A** to **C**) Cvi-0 is insensitive to Tre*_Mi_*31, unlike Col-0. Col-0 seedlings, but not Cvi-0, display root pigmentation (A), growth inhibition (B), and *CYP71A12* expression (C) upon treatment with 50 μM Tre*_Mi_*31. Four seedlings were analyzed per treatment. Scale bars, 100 μm. Box plots show median (line), interquartile range (box), range (whiskers), and mean (cross); individual points represent 10 biological replicates; different letters denote significant differences (*P* ≤ 0.05, one-way ANOVA with Tukey’s test). In (C), *CYP71A12* transcript levels were measured by reverse transcription quantitative polymerase chain reaction (RT-qPCR) after 6 hours of 50 μM Tre*_Mi_*31 treatment, normalized to the U-box housekeeping transcript *AT5G15400*. Data represent means ± SE of three technical replicates; different letters denote significant differences (*P* ≤ 0.05, one-way ANOVA with Tukey’s test). Experiments were repeated three times with consistent results. (**D**) Whole-genome sequencing of insensitive F_2_ individuals from Col-0 × Cvi-0 crosses and phenotyping of RILs revealed a genomic region on chromosome 3 associated with Tre*_Mi_*31 insensitivity (fig. S18). SNP substitution ratios [Cvi-0/(Col-0 + Cvi-0)] were averaged within 0.05-Mb windows and color-coded by frequency (30 to 50%, 50 to 70%, 70 to 90%, and 90 to 100%). The closest marker identified was C3_22147, with C3_20729 as the second closest. Sanger sequencing narrowed the candidate region to 78 kb between At3g59690 and At3g59890, encompassing 20 genes (table S10). T-DNA insertion screening identified *lecrk-V.5* (SALK_133163: *lecrk-V.5-3*) as showing reduced Tre*_Mi_*31 sensitivity (table S11). Four seedlings were analyzed per treatment. Scale bars [(A) and (D)], 100 μm. (**E**) Tre*_Mi_*31 induces *LecRK-V.5* expression. Transcript levels were measured by RT-qPCR after 6 hours of 40 μM Tre*_Mi_*31 treatment, normalized to *AT5G15400*. Two seedlings per treatment in each biological replicate. Data represent means ± SE of three biological replicates; asterisks indicate significant differences (***P ≤ 0.001, Student’s *t* test).

To elucidate the molecular mechanisms involved in Tre*_Mi_*31 recognition, we first investigated whether known signaling components, including the nematode receptor NILR1 and the co-receptors such as BAK1 (BRI1-ASSOCIATED KINASE 1), BKK1 (BAK1-LIKE 1), and SOBIR1 (SUPPRESSOR OF BIR1-1), were required. Tre*_Mi_*31-induced responses remained unchanged in the *nilr1-2* (*19*), *bak1-5 bkk1* (*41*, *42*), and *sobir1-13* (*43*) mutants (fig. S15). Then, we assessed root pigmentation and abnormality and seedling growth inhibition across various *Arabidopsis* accessions, identifying several accessions insensitive to Tre*_Mi_*31 (figs. S16 and S17). Among them, Cvi-0 failed to induce root pigmentation and abnormality, seedling growth inhibition, and *CYP71A12* expression in response to Tre*_Mi_*31 (Fig. 2, A to C), suggesting that Tre*_Mi_*31 recognition or its downstream signaling is specifically disrupted in this accession. A cross between Col-0 and Cvi-0 showed that all F_1_ plants responded to Tre*_Mi_*31, and the F_2_ population exhibited a 3:1 segregation ratio, indicating that a single dominant locus in Col-0 controls the response to Tre*_Mi_*31. To identify this locus, we collected 200 nonresponsive F_2_ seedlings and performed whole-genome bulk sequencing (Fig. 2D). Single-nucleotide polymorphism (SNP) analysis revealed significant enrichment of Cvi-0–derived SNPs at the distal end of chromosome 3 (fig. S18). Further phenotyping of 365 recombinant inbred lines (RILs) between Col-0 and Cvi-0 identified the locus *c3_22147* as most closely linked to the causal gene, along with the nearby locus *c3_20729* (table S9) (*44*), aligning with the chromosomal region identified by bulk sequencing. While most RILs containing Cvi-0 alleles at these loci were insensitive to Tre*_Mi_*31, and those with Col-0 alleles were sensitive, a few exceptions arose due to homologous recombination between these loci. The SNP analysis of these exceptional RILs via Sanger sequencing further narrowed the genomic region to 78 kb (table S10), containing 20 genes. Among these, four potential candidates were identified, all encoding highly similar lectin receptor kinases: LecRK-V.5, LecRK-V.6, LecRK-V.7, and LecRK-V.8 (fig. S19). T-DNA insertion mutants for candidate genes were tested (table S11), and the mutant SALK_133163 (*lecrk-V.5-3*) (*45*), which has a T-DNA insertion in the exon of *LecRK-V.5*, exhibited reduced root pigmentation (Fig. 2D). Notably, *LecRK-V.5* is transcriptionally up-regulated by Tre*_Mi_*31 (Fig. 2E), a characteristic feature of PRRs, and shows strong coexpression with *NILR1* (fig. S20). In contrast, T-DNA insertions in *LecRK-V.6*, *LecRK-V.7*, and *LecRK-V.8* did not affect root pigmentation, seedling growth inhibition, or *CYP71A12* expression upon Tre*_Mi_*31 treatment (fig. S21). These findings suggest that *LecRK-V.5* plays a major role in Tre*_Mi_*31-induced responses.

**Fig. 3.**
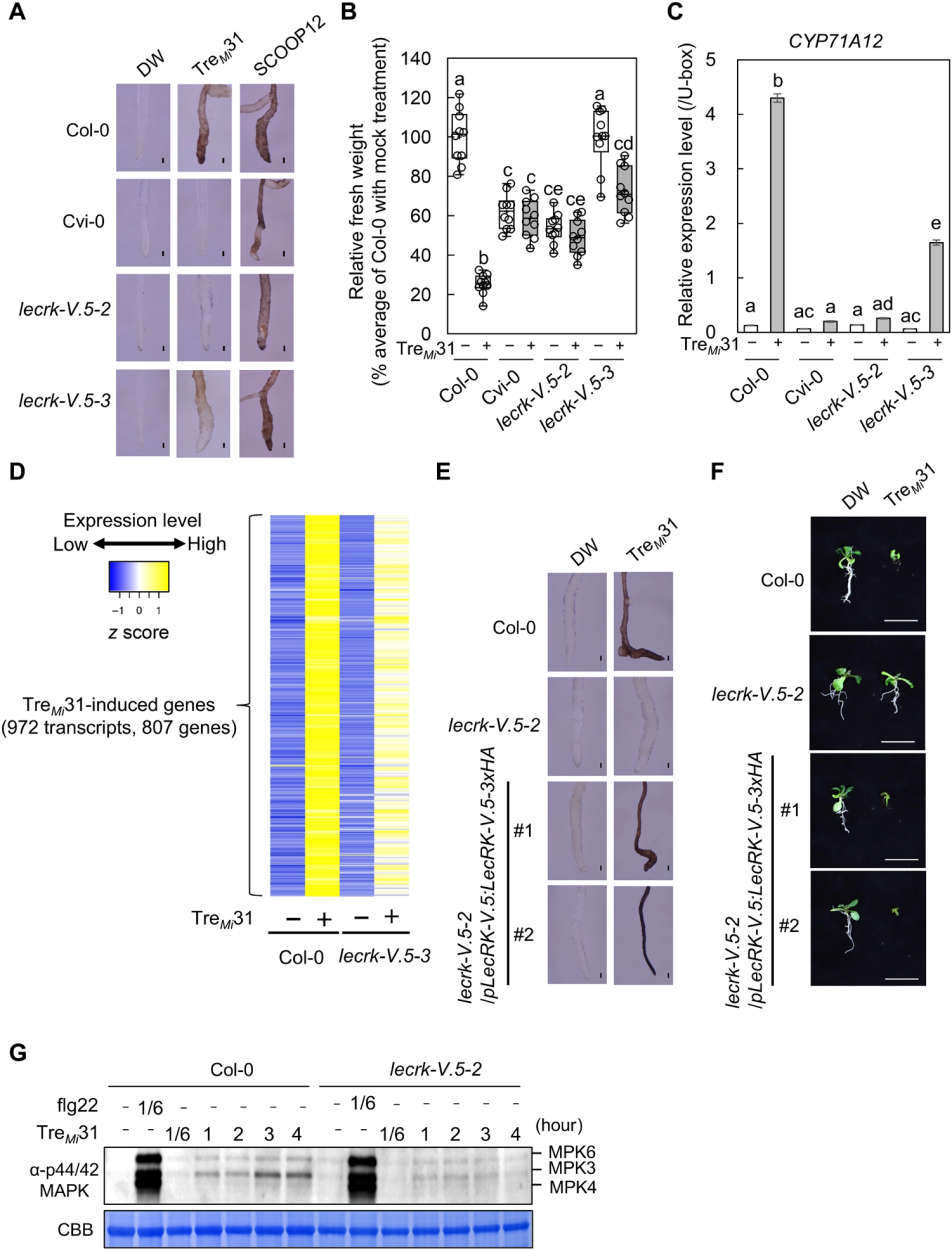
*lecrk-V.5* mutants have defects in Tre*_Mi_*31 signaling. (**A** to **C**) *lecrk-V.5-3* mutants show reduced responses, whereas *lecrk-V.5-2* mutants show no response to Tre*_Mi_*31. Col-0, but not Cvi-0 or *lecrk-V.5-2*, displayed root pigmentation (A), growth inhibition (B), and *CYP71A12* expression (C) upon treatment with 50 μM Tre*_Mi_*31, while all genotypes responded to 2 μM SCOOP12. Four seedlings were analyzed per treatment. Scale bars, 100 μm. Box plots show median (line), interquartile range (box), range (whiskers), and mean (cross); individual points represent 10 biological replicates; different letters denote significant differences (*P* ≤ 0.01, one-way ANOVA with Tukey’s test). In (C), *CYP71A12* transcript levels were measured by RT-qPCR after 6 hours of 50 μM Tre*_Mi_*31, normalized to the U-box housekeeping transcript *AT5G15400*. Values represent means ± SE of three technical replicates, with different letters indicating significant differences (*P* ≤ 0.05, one-way ANOVA with Tukey’s post hoc test). Experiments were repeated three times with consistent results. (**D**) RNA-seq shows reduced induction of Tre*_Mi_*31-upregulated genes (logFC ≥1, adjusted *P* ≤ 0.01) in *lecrk-V.5-3* compared to Col-0 after 12 hours of 30 μM Tre*_Mi_*31 treatment. (**E** and **F**) Expression of *LecRK-V.5-3xHA* under its native promoter rescues root pigmentation (E) and growth inhibition (F) in *lecrk-V.5-2* mutants treated with 50 μM Tre*_Mi_*31. T_2_ complementation lines with the transgene were used for the assay, and homozygous individuals were retrospectively identified on the basis of T3 segregation. At least four confirmed homozygous seedlings were analyzed per treatment. Scale bars, 100 μm (E) and 1 cm (F). Experiments were repeated three times with consistent results. (**G**) Col-0, but not *lecrk-V.5-2*, showed slow, sustained MAPK activation upon 50 μM Tre*_Mi_*31 treatment. The flg22 (1 μM) served as a positive control. Experiments in (E) to (G) were repeated three times with consistent results.

To confirm the role of *LecRK-V.5* in Tre*_Mi_*31 signaling, we examined the phenotypes of two mutant alleles, *lecrk-V.5-3* and *lecrk-V.52* (GK_623G01) (fig. S22, A and B) (*46*). The *lecrk-V.5-2* mutant displayed an almost complete loss of sensitivity to Tre*_Mi_*31, while *lecrk-V.5-3* exhibited a partial response, characterized by reduced root pigmentation, restored seedling growth inhibition, and diminished *CYP71A12* expression (Fig. 3, A to C, and fig. S22C). In contrast, Col-0, Cvi-0, *lecrk-V.5-2,* and *lecrk-V.5-3* mutants induce root pigmentation in response to SCOOP12 (Fig. 3A). Notably, *lecrk-V.5-2* contains a T-DNA insertion in the 3′ half of the gene, whereas *lecrk-V.5-3* has an insertion in the 5′ end (fig. S22A). The *lecrk-V.5-2* showed higher expression of the 5′ region, encoding the ectodomain with the transmembrane region but lacking the kinase domain (fig. S22D). This truncated form may act in a dominant-negative manner, potentially through heterodimerization with other LecRKs or competition for ligand binding, whereas *lecrk-V.5-3* is a true knockout mutant. RNA-seq analysis further demonstrated that nearly all Tre*_Mi_*31-induced genes were downregulated in the *lecrk-V.5-3* mutant (Fig. 3D), indicating that *LecRK-V.5* is important for the recognition or very early stages of the Tre*_Mi_*31 signaling pathway (table S12). This conclusion was further supported by the restoration of Tre*_Mi_*31 responses in the *lecrk-V.5-2* mutant following complementation with *LecRK-V.5-3xHA* under its native promoter (*lecrk-V.5-2 /pLecRK-V.5:LecRK-V.5-3xHA*), with the complementation lines exhibiting even stronger responsiveness, likely due to higher expression levels (Fig. 3, E and F, and fig. S22E). To further explore the role of *LecRK-V.5* in downstream signaling events, we compared the effects of Tre*_Mi_*31 and flg22 on MAPK activation. Tre*_Mi_*31 induced slower and more prolonged MAPK activation than flg22 (Fig. 3G) and did not trigger ROS production (fig. S22F), suggesting that Tre*_Mi_*31 induces downstream pathways that are, at least partially, distinct from most identified MAMP receptors.

**Fig. 4.**
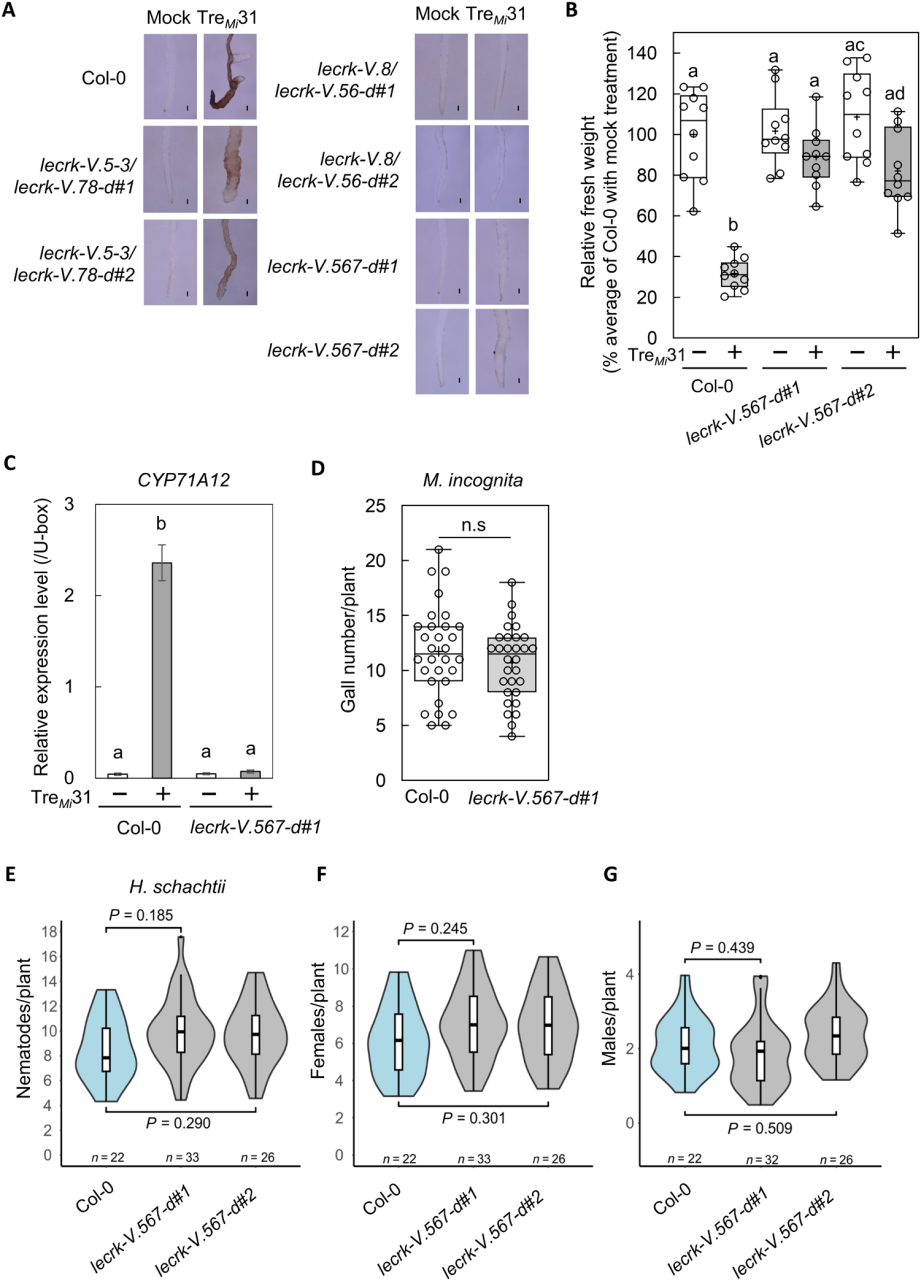
*LecRK-V.5* and *LecRK-V.6* are involved in Tre*_Mi_*31 recognition. (**A**) Root pigmentation upon 50 μM Tre*_Mi_*31 treatment is reduced in the CRISPR deletion line *lecrk-V.5-3/lecrk-V.78-d* and absent in *lecrk-V.567-d* and *lecrk-V.8/lecrk-V.56-d* mutants. Black bars represent 100 μm. (**B** and **C**) *lecrk-V.567-d* mutants fail to show seedling growth inhibition (B) or induction of *CYP71A12* expression (C) in response to 50 μM Tre*_Mi_*31. Box plots show median (line), interquartile range (box), range (whiskers), and mean (cross); individual points represent 10 biological replicates; different letters denote significant differences (*P* ≤ 0.01, one-way ANOVA with Tukey’s test). In (C), transcript levels of *CYP71A12* were measured by RT-qPCR after 6 hours of treatment, normalized to the U-box housekeeping transcript *AT5G15400*. Two seedlings were used per treatment per replicate. Data represent means ± SE of three biological replicates; different letters indicate significant differences (*P* ≤ 0.0001, one-way ANOVA with Tukey’s post hoc test). Experiments were repeated three times with consistent results. (**D**) Nematode infection assay using *M. incognita* (~80 J2s per plant) revealed no significant difference (n.s.) in gall formation between *lecrk-V.567*-d mutants and Col-0 plants (*n* = 30, Student’s *t* test). Experiments were repeated three times with consistent results. (**E** to **G**) Number of total (E), female (F), and male (G) *H. schachtii* CNs per root system at 14 days post-infection (dpi) after inoculation with ~80 J2s per plant. Violin plots show kernel density estimates (polygons), box limits (25th and 75th percentiles), median (thick line), whiskers extending to ≤1.5× the interquartile range (IQR), and outliers (dots). Data shown are from one representative experiment out of three independent experiments with consistent results. To account for environmental variation, data were analyzed using a best linear unbiased estimators model (R v4.2.1, lme4 package); pairwise differences were assessed using the emmeans package and added manually.

As the *lecrk-V.5-3* mutant did not completely abolish the Tre*_Mi_*31 response, we hypothesized that the other LecRK-V(s) might also contribute to the signaling pathway. To investigate this, we generated CRISPR-mediated *lecrk-V.5-3/lecrk-V.78-d* mutants, which lack the region between *LecRK-V.7* and *LecRK-V.8* in the *lecrk-V.5-3* background (fig. S23). These mutants exhibited a weak yet noticeable response to Tre*_Mi_*31 including root pigmentation and shoot growth inhibition, suggesting a role for *LecRK-V.6* in Tre*_Mi_*31 recognition (Fig. 4A and fig. S24A). In contrast, *lecrk-V.8/lecrk-V.56-d* (lacking regions from *LecRK-V.5* to *LecRK-V.6* in *lecrk-V.8* background) and *lecrk-V.567-d* (spanning *LecRK-V.5* to *LecRK-V.7*) showed no response to Tre*_Mi_*31, indicating that *LecRK-V.7* and *LecRK-V.8* are not involved in Tre*_Mi_*31 recognition (Fig. 4, A to C, and fig. S24A). In addition, *lecrk-V.567-d* did not respond to Tre*_Ce_*24 and trehalase-derived peptides from PPNs (fig. S24, B and C), suggesting that *LecRK-V.5* and *LecRK-V.6* are required for the response to Tre*_Mi_*31 and other PPN trehalase-derived peptides. Transcriptomic data from the electronic Fluorescent Pictograph (eFP) browser indicate that *LecRK-V.5* and *LecRK-V.6* are expressed in most root tissues, with *LecRK-V.5* showing relatively high expression in procambial cells and *LecRK-V.6* in cortical cells (fig. S25 and table S13). The phylogenetic analysis of their ectodomains reveals that LecRK-V.5 and LecRK-V.6, along with the closely related LecRK-Vs (3, 4, 7, and 8), are exclusive to the Brassicales (fig. S26). To evaluate whether Tre*_Mi_*31 recognition contributes to resistance against PPNs, we compared the number of galls formed by *M. incognita* in Col-0 and *lecrk-V.567-d* mutants. No significant difference was observed (Fig. 4D), suggesting that Tre*_Mi_*31 recognition plays only a modest role in resistance. We also assessed susceptibility to *H. schachtii*, as a secreted trehalase (*Hsc_gene_15468*) in CNs has been identified as a candidate apoplastic virulence effector, with transcripts highly enriched in subventral gland cells during the early migratory stage (fig. S10B) (*30*–*32*). A synthetic peptide derived from Hsc_gene_15468 induces root pigmentation and *CYP71A12* expression in a LecRK-V.5/V.6–dependent manner (figs. S11A, S24B, and S24C).

Based on this immune activation, we examined whether LecRK-V.5/V.6 contribute to resistance against *H. schachtii*. Although *lecrk-V.567-d* mutants tended to show a slight increase in susceptibility and increased the number of females than Col-0, the difference was not statistically significant at *P* < 0.05 (Fig. 4, E to G) (*47*).

### LecRK-V.5 and LecRK-V.6 are required for the induction of immune responses by trehalase-derived peptides from phytophagous insects and pathogenic fungi

**Fig. 5.**
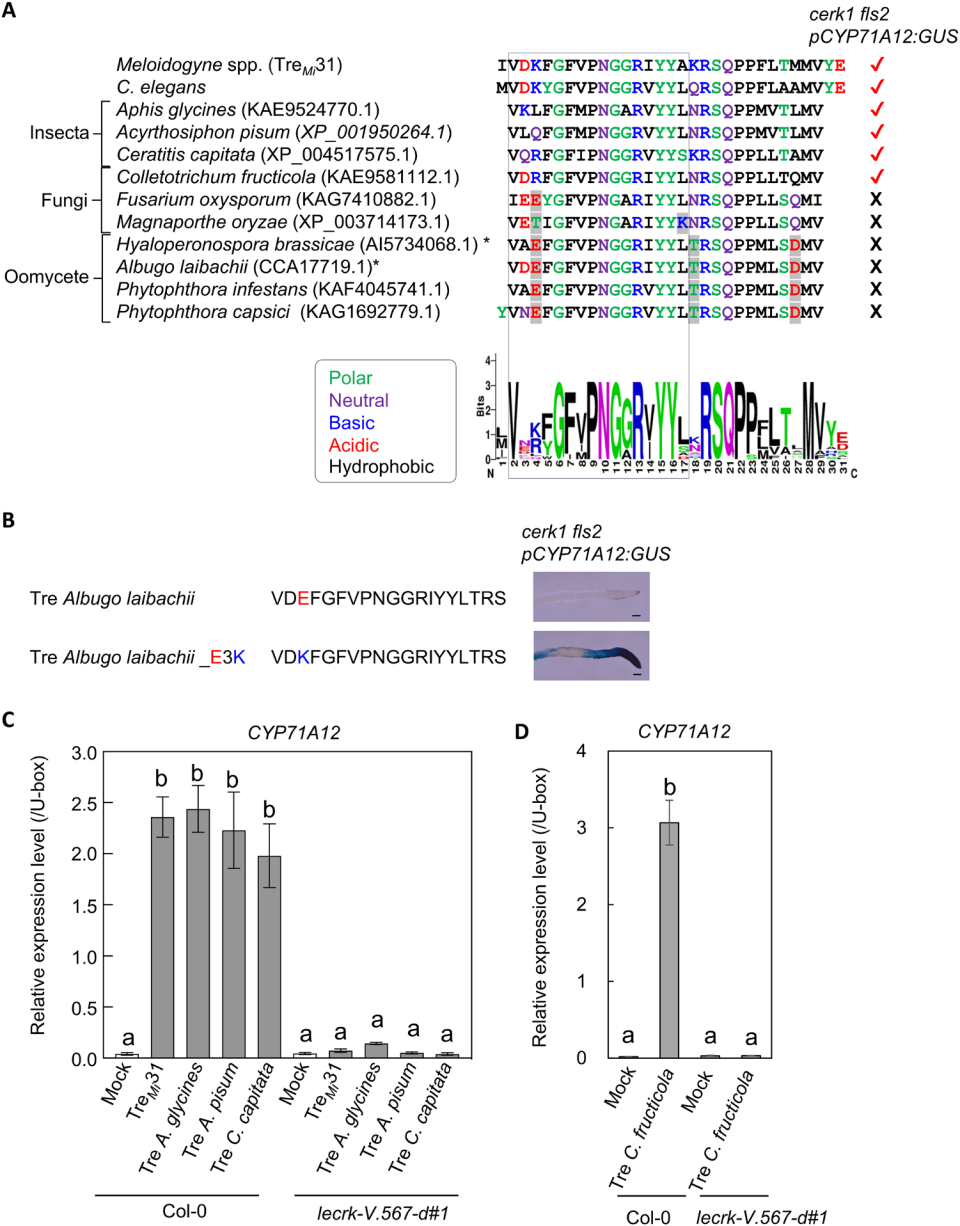
*LecRK-V.5* and *LecRK-V.6* are involved in the recognition of trehalase peptides from phytophagous insects and pathogenic fungi. (**A**) Sequence alignment of trehalase peptides from *C. elegans*, *M. incognita*, phytophagous insects, fungi, and oomycetes, with their MAMP activities determined via the *CYP71A12* expression assay using Tre*_Ce_*19 (fig. S9A). The sequence logo highlights conserved residues in MAMP active peptides from nematodes (fig. S11A), insects, and fungi. Trehalases in *Hyaloperonospora brassicae* (AI5734068.1) and *Albugo laibachii* (CCA17719.1) do not have signal peptides predicted by SignalP-6.0 (*). Amino acid residues highlighted in gray differ in properties from those found in MAMP active peptides. (**B**) A charge-reverse substitution of the third glutamic acid residue to lysine (E3K) in a MAMP-inactive trehalase peptide from *A. laibachii* restores MAMP activity. *CYP71A12* expression was analyzed upon treatment with 50 μM peptide in the *cerk1 fls2 pCYP71A12:GUS* line. Black bars represent 100 μm. (**C** and **D**) Col-0 seedlings, but not the *lecrk-V.567-d* line, show *CYP71A12* expression in response to trehalase-derived peptides from insects (C) and fungi (D). Transcript levels of *CYP71A12* in the seedlings upon treatment with 50 μM trehalase-derived peptides were measured by RT-qPCR after normalization to the U-box housekeeping transcript *AT5G15400*. Data represent means ± SE of three biological replicates, with different letters indicating significant differences (*P* ≤ 0.0001, one-way ANOVA with Tukey’s post hoc test). Experiments were repeated three times with consistent results.

Given the high conservation of secreted trehalases across higher organisms, we conducted a Basic Local Alignment Search Tool (BLAST) search to identify sequences similar to Tre*_Mi_*31 in other plant pathogenic organisms. Several phytophagous insects and fungal and oomycetes pathogens were found to have sequences corresponding to Tre*_Mi_*31 (Fig. 5A and fig. S27). In addition, similar sequences were also identified in nonphytophagous insects and nonpathogenic fungi and oomycetes (fig. S28). To assess potential MAMP activity, we synthesized the peptides from selected phytophagous insects and pathogenic fungi and oomycetes and evaluated their MAMP activity by using the *cerk1 fls2 pCYP71A12:GUS* line. The results showed clear MAMP activity in peptides derived from the fungal pathogen *Colletotrichum fructicola* and phytophagous insects, including aphids (*Aphis glycines* and *Acyrthosiphon pisum*) and the Mediterranean fruit fly (*Ceratitis capitata*) (fig. S29A). Sequence comparison revealed that the MAMP active peptides tended to have more positively charged residues (K, R, and H) at the fourth position from the N terminus, while nonactive peptides were more enriched in negatively charged residues (E) (Fig. 5A). Consistently, substituting positively charged residues with negatively charged ones in Tre*_Ce_*19 and Tre*_Mi_*19 abolished their MAMP activity (fig. S29, B and C). Conversely, introducing a reverse-charge mutation (negative to positive) in the nonactive peptide from the oomycete pathogen *Albugo laibachii* activated its MAMP activity (Fig. 5B), underscoring the importance of the positively charged residue at this position. Furthermore, the *lecrk-V.567-d* mutant failed to induce root pigmentation and *CYP71A12* expression in response to peptides from phytophagous insects and fungal pathogens, highlighting the role of *LecRK-V.5* and *LecRK-V.6* in mediating broad-spectrum pathogen recognition through trehalase-derived peptides (Fig. 5, C and D, and fig. S29D).

## DISCUSSION

This study elucidates how plants recognize PPNs through trehalase-derived peptides, identifying *LecRK-V.5* and *LecRK-V.6* as essential components of this immune signaling pathway (fig. S30). The conservation of this peptide region in trehalases across PPNs, as well as in phytophagous insects and fungal pathogens, underscores the significant role of LecRK-Vs in response to a broad range of pathogens.

Trehalose plays diverse and essential roles in various organisms, including bacteria, fungi, nematodes, and insects. It serves as an energy and carbon source and acts as a stress protectant, stabilizing proteins and membranes under extreme conditions such as heat, cold, desiccation, and osmotic stress (*48*). Trehalose is particularly critical for pathogenic organisms, as mutants of bacterial pathogens (*49*, *50*) and fungal pathogens (*51*) defective in trehalose biosynthesis exhibit significantly reduced virulence in plants, suggesting its possible protective effects against host immunity. Pathogenic organisms also induce trehalose accumulation in the host by activating host trehalose biosynthesis pathways (*49*, *52*). Enhanced host trehalose biosynthesis increases susceptibility to oomycete pathogens, while inhibition of trehalose biosynthesis suppresses disease (*52*), highlighting trehalose’s role as a susceptibility factor. Accumulated trehalose may support pathogen survival under host immune defenses and provide energy during parasitism (*53*), facilitating the development and colonization of pathogenic organisms. Similarly, PPNs induce trehalose accumulation in specialized feeding structures, such as galls (*54*) and syncytia (*55*), likely aiding their growth and survival. Notably, transgenic soybean hairy roots overexpressing nonsecreted trehalase, potentially reducing trehalose levels, showed increased resistance to CNs, emphasizing the importance of trehalose levels in plant tissues in determining susceptibility to nematode infections (international patent publication number: WO2008095919A1).

Consistently, trehalase enzymes are crucial for the survival of many microorganisms, serving diverse roles essential for their physiology and pathogenicity. The significance of trehalases in microbial survival is highlighted by their use as targets in agricultural fungicides and insecticides (*56*). For example, the trehalase inhibitor validamycin is commonly used to control rice sheath blight caused by *Rhizoctonia solani* (*56*). Trehalases are also implicated in virulence and parasitism of PPNs. Host-induced gene silencing of nonsecreted trehalase in *M. incognita* within transgenic tobacco significantly reduces egg production and root-knot formation (*57*), suggesting a critical role for nonsecreted trehalase in parasitism, potentially through its involvement in chitin biosynthesis required for eggshell formation.

While the roles of nonsecreted trehalases in parasitism are well-documented, the function of secreted trehalases remains less explored. Secreted trehalases are conserved across diverse taxa, including bacteria, fungi, oomycetes, nematodes, and insects, suggesting important roles for their survival. Pathogenic *Candida* species secrete trehalases during host colonization, whereas their mammalian hosts do not produce trehalose (*58*–*61*). Knockout mutants of *Candida* trehalases show significantly reduced infectivity, demonstrating their essential role in virulence. These secreted trehalases likely exploit trehalose released from other microbial sources or dying cells within the host environment, providing a readily accessible nutrient and energy source during infection. In PPNs, secreted trehalases are released during infection to use host-derived trehalose. The higher expression of secreted trehalases in CNs and RKNs (fig. S10), coupled with the recognition of secreted trehalases by *Arabidopsis* suggests an important role for these enzymes in nematode survival and parasitism within host plants. Although the broad taxonomic conservation of secreted trehalases across both pathogenic and nonpathogenic organisms supports their classification as MAMPs, some may acquire additional effector-like functions in pathogenic species. Meanwhile, the precise role of apoplastic trehalose in plant-PPN interactions remains unclear. Trehalase-derived peptides may thus occupy a functional space between canonical MAMPs and lineage-specific effectors.

Some LecRKs function as immune receptors, recognizing MAMPs and damage-associated molecular patterns. For instance, DORN1 (LecRK-I.9) recognizes extracellular adenosine 5′-triphosphate (*62*), while LecRK-VI.2 is a potential receptor for extracellular nicotinamide adenine dinucleotide (NAD^+^) and NAD^+^ phosphate (*63*). Similarly, LecRK-VI.8 may be involved in the recognition of extracellular NAD^+^ (eNAD^+^) but not extracellular NADP^+^ (eNADP^+^) (*64*). In addition, LecRK-I.1 and LecRK-I.8 are implicated in the perception of insect eggs, although the specific egg-derived ligands remain unidentified (*65*, *66*). Our findings indicate that LecRK-V.5 and LecRK-V.6 participate in immune responses to trehalase-derived peptides from PPNs, insect parasites, and fungal pathogens. This suggests secreted trehalases are highly conserved MAMPs across species, while they may also function as virulence effectors in some pathogenic organisms such as CNs. Notably, *LecRK-V.5* is transcriptionally up-regulated by Tre*_Mi_*31 (Fig. 2E) and is closely coexpressed with *NILR1* (fig. S20). Moreover, *LecRK-V.5* is transcriptionally up-regulated in *Arabidopsis* tissues surrounding CNs during their migratory stage (*19*), aligning well with the significant up-regulation of secreted trehalase (*Hsc_gene_15468*) during this phase. These findings suggest a possibility that LecRK-V.5 and LecRK-V.6 may function as receptors or components of receptor complexes for trehalase-derived peptides, although further studies are needed to confirm direct interactions. Another possibility is that the trehalase MAMP is directly recognized by another receptor, such as an LRR-RLK that often binds to peptide ligands, and LecRK-V.5 and LecRK-V.6 are activated as downstream components.

Although Tre*_Mi_*31-induced immune responses were dependent on *LecRK-V.5* and *LecRK-V.6*, the contribution of this recognition to resistance against PPNs appeared modest (Fig. 4, D to G). This may be due to the robust virulence mechanisms used by these nematodes to suppress host immunity or to the presence of additional MAMPs recognized by other PRRs, which may buffer the impact of trehalase perception. Another possibility is that Col-0 expresses *LecRK-V.5* and *LecRK-V.6* at low levels, potentially reflecting fitness trade-offs associated with constitutive PRR signaling. Supporting this hypothesis, transgenic complementation lines expressing *LecRK-V.5* under its native promoter showed stronger responsiveness to Tre*_Mi_*31, likely due to elevated receptor expression (Fig. 3, E and F, and fig. S22E). These findings suggest that the full immune potential of trehalase perception may not be realized in Col-0 due to insufficient expression of the relevant receptors. Enhancing *LecRK-V.5* or *LecRK-V.6* expression—either through promoter engineering or transgenic introduction into heterologous species—could potentiate immune signaling and disease resistance. Given the broad conservation of trehalase-derived peptides across nematodes, insects, and fungi and the apparently unique recognition of these peptides by LecRK-V.5 and LecRK-V.6 in the Brassicales lineage (fig. S26), transferring this recognition system to other plant species may provide a strategy to enhance immunity against diverse pathogens in economically important crops (*67*).

## MATERIALS AND METHODS

### Plant materials and growth conditions

Seedlings of *A. thaliana* (L.) Heynh were grown on a half-strength MS medium containing 1% sucrose with or without 0.8% agarose under a continuous light photoperiod or a long-day photoperiod (16-hour light and 8-hour dark) at 23°C. The light is provided by light-emitting diodes (65 to 75 μE m^−2^ s^−1^ for *Arabidopsis*). The humidity was maintained at 60 to 70%.

### Extraction of *C. elegans* and *M. incognita* for MAMP activity assays

*C. elegans* and *M. incognita* were dissolved in the extraction buffer [20 mM MES (pH 5.6) and 0.1% CHAPS detergent (FUJIFILM Wako Pure Chemical Corporation, Osaka, Japan)], and proteins were extracted by sonication. The crude extract was centrifuged at 12,000*g* for 10 min and repeated more than three times to remove debris. The protein concentration was determined by Bradford protein assay (Bio-Rad, Hercules, CA). An equivalent volume of the extraction buffer was treated to the samples as a mock control. Although the extract was prepared multiple times, each preparation consistently induced the same immune responses.

### GUS assay of *cerk1 fls2 pCYP71A12:GUS* line

GUS staining was performed on 10- to 14-day-old seedlings of the *cerk1 fls2 pCYP71A12:GUS* reporter line after treatment with extract or peptides for 24 hours. Seedlings were vacuum infiltrated with staining solution [50 mM sodium phosphate buffer (pH 7.0), 0.5% Triton X-100, 1 mM potassium ferrocyanide, 1 mM potassium ferricyanide, 5% methanol, and 1 mM 5-bromo-4-chloro-3-indolyl d-glucuronide (X-gluc)] and then incubated at 37°C for 30 min to 1 hour.

### Seedling growth inhibition and root pigmentation assay

*Arabidopsis* seeds were sown on half-strength MS agar plates containing 1% sucrose and 0.8% agar. After cold treatment for 2 to 3 days at 4°C, plates were transferred to a growth chamber under continuous light at 23°C. Five-day-old seedlings were then moved to liquid half-strength MS medium with 1% sucrose and treated with extract or peptides for 7 days. Fresh weight was measured using an analytical balance (Mettler Toledo, Zurich, Switzerland), and root pigmentation was observed under a light microscope.

### Peptide synthesis

All peptides were synthesized by GenScript (Piscataway, NJ) with purities exceeding 70%. Trifluoroacetic acid was exchanged for phosphate. The pH of each peptide solution was checked and neutralized as necessary.

### Refinement of secreted trehalase gene models and expression analysis in *M. incognita*

The current gene annotations in *M. incognita* incompletely capture secreted trehalase-encoding genes, possibly due to limited RNA-seq coverage during early infection stages when these genes are expressed. To refine gene prediction of secreted trehalases, we performed tblastn searches using the Tre*_Mi_*31 peptide sequence (IVDKFGFVPNGGRIYYAKRSQPPFLTMMVYE) as a query against the genome assemblies of *M. incognita* (BioProject PRJEB61149 and PRJEB8714) (*68*, *69*). Additional tblastn searches were conducted against Iso-Seq transcriptome datasets from *M. incognita* (BioProject PRJNA787737) (*70*) and *Meloidogyne javanica* (BioProject PRJNA939015) (*71*) to identify full-length cDNA reads corresponding to trehalase genes. These analyses revealed misannotations and allowed us to manually curate three gene models—*Mi*Tre-1, *Mi*Tre-2, and *Mi*Tre-3—each encoding a secreted trehalase with an N-terminal signal peptide and the Tre*_Mi_*31 peptide region (table S14 and figs. S7 and S8). Full-length Iso-Seq transcripts support the accuracy of these refined models: We identified a transcript in *M. incognita* (SRR17191357.374462) that is 100% identical to *Mi*Tre-2 and a transcript in *M. javanica* (SRR23627520.394042) that shares 99.5% cDNA sequence identity with *Mi*Tre-1, with minor differences likely reflecting species-specific variation (figs. S7 and S8). To further validate these models, we aligned cleaned reads from a stage-specific RNA-seq dataset covering egg, J2, J3, J4, and female stages (BioProject PRJNA3900559) (*72*) to the *M. incognita* genome and examined the splicing patterns and gene expression levels of trehalase-encoding genes. A manually modified annotation file was prepared by editing a GFF3 file available at (https://doi.org/10.57745/7KZVMP) (*68*). Raw paired-end reads were quality-checked and trimmed using fastp (v0.23.4) with the parameter -l 50 (*73*) and then mapped using the two-pass mode in STAR (v2.7.11b), with the manually refined annotation used for genome indexing (*74*). Transcripts per million of trehalase genes were estimated using RNA-Seq by Expectation-Maximization (RSEM) (v.1.3.1) (*75*). RNA-seq read mapping showed strong support across the entire regions of *Mi*Tre-1 and *Mi*Tre-3, whereas the N-terminal region of *Mi*Tre-2 exhibited reduced coverage, likely due to sequence variation between the strains used for genome sequencing and RNA-seq analysis or unknown technical factors (fig. S6). Nevertheless, the RNA-seq mapping strongly supports the correctness of the *Mi*Tre-1 and *Mi*Tre-3 gene models, and the presence of a full-length Iso-Seq transcript that is 100% identical to *Mi*Tre-2 confirms the accuracy of the *Mi*Tre-2 gene model.

### RNA-seq and differential gene expression analyses

Ten-day-old *Arabidopsis* seedlings of Col-0 or *efr fls2 cerk1* mutants, grown in liquid half-strength MS medium with 1% sucrose, were treated with *C. elegans* extract (100 μg/ml) for 0.5, 1, 3, 6, and 12 hours (Fig. 1, C and D, and table S1), with *M. incognita* extract (100 μg/ml) for 12 hours (Fig. 1C and table S2) and with 50 μM Tre*_Mi_*31 for 12 hours (Fig. 1, J and K, and table S7), with three biological replicates per condition. In addition, 10-day-old seedlings of Col-0, *lecrk-V.5-3* (Fig. 3D and table S12) were treated with 30 μM Tre*_Mi_*31 for 12 hours with four biological replicates per genotype. Transcript levels were analyzed by RNA-seq, with library preparation performed using the BradSeq protocol (*76*). Single-end 86-bp reads were sequenced on an Illumina NextSeq 500 platform and mapped to the *Arabidopsis* cDNA reference based on TAIR10 using Bowtie v0.12.9 (*77*). Read counts were obtained per transcript model (*78*), and reads per million mapped reads were calculated. Differentially expressed genes (DEGs) with a false discovery rate (FDR) ≤ 0.01 were identified using edgeR. Sequencing reads have been deposited in the DNA Data Bank of Japan under accession number PRJDB19783 (BioProject: SAMD00851270-SAMD00851330).

### Identification of genomic region for Tre*_Mi_*31 insensitivity in Cvi-0 by sequencing

Cvi-0 and Col-0 were crossed to produce F_1_ plants, which were then used to generate an F_2_ population. Root pigmentation assays with 50 μM Tre*_Mi_*31 peptide were performed on the F_2_ population. A total of 200 F_2_ lines insensitive to 50 μM Tre*_Mi_*31 peptide were pooled, and their genomic DNA (gDNA) was extracted using QIAGEN DNeasy Plant Mini Kits (QIAGEN, Venlo, the Netherlands). For DNA library preparation, gDNA was sheared to a size of ~450 to 500 base pairs using the Covaris S2 system (Covaris, Woburn, Massachusetts), and DNA libraries were prepared using the Ovation Rapid DR Multiplex system (NuGEN Technology, Männedorf, Switzerland) according to the manufacturer’s instructions. DNA sequencing was performed with the NextSeq500 (Illumina, San Diego, CA), and potential causal SNPs were identified as previously described (*79*).

### Identification of genomic marker closest to causal mutation by RIL lines

The Cvi-0 x Col-0 RIL population was obtained from INRAE Versailles (*44*). Root pigmentation assays with 50 μM Tre*_Mi_*31 peptide were performed for 365 RIL lines. The positions of genetic markers linked to the phenotype were narrowed down with the genotyping and phenotyping data of RILs (table S9).

### T-DNA insertion lines

T-DNA insertion mutant lines—*lecrk-V.5-2* (GK_623G01) (*46*), *lecrk-V.5-3* (SALK_133163C), *lecrk-V.6* (SALK_009623C), *lecrk-V.7* (SALK_209200C), and *lecrk-V.8* (SALK_202952C)—and other lines listed in table S11 were obtained from the Arabidopsis Biological Resource Center at the Ohio State University. Previously published lines were *bak1-5 bkk1* (*41*, *42*), *fls2* (*7*), *cerk1* (*11*), *nilr1-2* (*19*), and *sobir1* (*43*). Homozygous *cerk1 fls2 pCYP71A12:GUS* line was generated by crossing homozygous lines and then selection by genotyping. Other methods are provided in Supplementary Methods.

### Statistics

Statistical significance was determined using *t* tests and one-way analysis of variance (ANOVA) in GraphPad Prism6 software (GraphPad Software, San Diego, CA, USA). Best linear unbiased estimators were applied using the “lme4” package in R (version 4.2.1), and pairwise comparisons, including those for nematode counts in Fig. 4 (E to G) were performed using the “emmeans” package.

## Supplementary Materials

### The PDF file includes:

Supplementary Methods Figs. S1 to S30 Legends for tables S1 to S15 References
