# Supplementary material for: A trehalase-derived MAMP triggers LecRK-V–mediated immune responses in Arabidopsis
Source: Sci Adv. 2025 Jul 30;11(31):eadv8896. doi: 10.1126/sciadv.adv8896 (PMC12309661; doi:10.1126/sciadv.adv8896)
Supplement: Supplementary file 1 — Supplementary Methods Figs. S1 to S30 Legends for tables S1 to S15 References [file sciadv.adv8896_sm.pdf]

Science Advances
AAAS

Supplementary Materials for

A trehalase-derived MAMP triggers LecRK-V–mediated immune responses in *Arabidopsis*

Erika Iino *et al.*

Corresponding author: Yasuhiro Kadota, yasuhiro.kadota@riken.jp; Ken Shirasu, ken.shirasu@riken.jp

Sci. Adv. **11**, eadv8896 (2025)
DOI: 10.1126/sciadv.adv8896

The PDF file includes:

Supplementary Methods
Figs. S1 to S30
Legends for tables S1 to S15
References

Other Supplementary Material for this manuscript includes the following:

Tables S1 to S15

Supplementary Methods

RNA-Seq and differential gene expression analyses

Ten-day-old *Arabidopsis* seedlings of Col-0 or *efr fls2 cerk1* mutants, grown in liquid half-strength MS medium with 1% sucrose, were treated with 100 µg/mL *Caenorhabditis elegans* extract for 0.5, 1, 3, 6, and 12 hours (Fig. 1, C and D, and Table S1); with 100 µg/mL *Meloidogyne incognita* extract for 12 hours (Table S2); and with 50 µM $Tre_{Mi}31$ for 12 hours (Fig. 1, J and K, and Table S7), with three biological replicates per condition. Additionally, ten-day-old seedlings of Col-0, *lecrk-V.5-3* (Fig. 3D and Table S12) were treated with 30 µM $Tre_{Mi}31$ for 12 hours with four biological replicates per genotype. Transcript levels were analyzed by RNA-Seq, with library preparation performed using the BradSeq protocol (*76*). Single-end 86-bp reads were sequenced on an Illumina NextSeq 500 platform and mapped to the Arabidopsis cDNA reference based on TAIR10 using Bowtie v0.12.9 (*77*). Read counts were obtained per transcript model (*78*) and Reads Per Million mapped reads (PPM) were calculated. Differentially expressed genes (DEGs) with an FDR ≤ 0.01 were identified using edgeR. Sequencing reads have been deposited in the DNA Data Bank of Japan (DDBJ) under accession number PRJDB19783 (BioProject: SAMD00851270-SAMD00851330).

Venn diagram analysis of transcript levels upon treatment with $Tre_{Mi}31$, flg22, and Chitin

Venn diagram analyses were performed using BioVenn (*80*). Genes upregulated or downregulated after treatment with flg22 (*25*), or chitin (*26*) were used.

Gene ontology (GO) term enrichment analysis

GO term enrichment analyses were performed using PANTHER (https://geneontology.org/) from the Gene Ontology Consortium.

Heatmap of transcript levels in Col-0 and *lecrk-V.5-3* upon treatment with $Tre_{Mi}31$

Heatmap was made using Heatmapper (*81*) (http://www.heatmapper.ca/).

Histochemistry of lignin deposition

Lignin deposition was visualized by phloroglucinol-HCl staining as previously described (*24*). Eight-day-old *Arabidopsis* Col-0 seedlings were treated with 100 µg/mL *C. elegans* extract, 50 µM $Tre_{Mi}31$, or distilled water (mock). Seedlings were fixed in a solution of ethanol: acetic acid (9:1, v/v) for 2 hours after a brief vacuum, washed twice with 90% ethanol for 30 min, and incubated overnight in distilled water. Samples were then stained with 2% phloroglucinol (w/v) in 20% HCl. Microphotographs were taken, processed as described and combined manually.

C. elegans culturing

C. elegans was cultured at 25°C on 6 cm Petri dishes containing Nematode Growth Medium (NGM) agar with a lawn of *E. coli* strain OP50. After 4 days on NGM agar plates, *C. elegans* were transferred to S medium supplemented with Streptomycin-Penicillin solution (100 units/mL, FUJIFILM Wako Pure Chemical Corporation, Osaka, Japan) and freeze-dried *E. coli* OP50 powder (2 g/500 mL, LabTIE, MOLGEN Veenendaal, The Netherlands) and grown on a rotary shaker at 25°C. On the fifth day, an additional 2 g/500 mL of freeze-dried *E. coli* OP50 powder was added, and the culture was incubated for another 3 days. After the incubation period, the liquid culture was cooled on ice, and *C. elegans* was collected by gravity sedimentation. The worms were

then thoroughly washed with water, filtered through filter paper by aspiration, and frozen using liquid nitrogen. As the *C. elegans* cultures were not synchronized, the extracted material included individuals at all developmental stages.

Purification of MAMPs from *C. elegans* by chromatography

Fifty to one hundred gram of *C. elegans* was dissolved in the Extraction Buffer (20 mM Tris, pH 8.0, 0.1% CHAPS Detergent (3-((3-cholamidopropyl) dimethylammonio)-1-propanesulfonate) (FUJIFILM Wako Pure Chemical Corporation), cOmplete Ultra Protease Inhibitor Cocktail (Merck, Rahway, New Jersey), and proteins were extracted by sonication. The crude extract was centrifuged at 12,000 g for 10 min and repeated more than three times to remove debris. Immunogenic proteins in the cleared crude extract were precipitated with 40–70% ammonium sulfate. The resulting precipitate was dissolved in Extraction Buffer, followed by debris and aggregate removal through centrifugation at 12,000 g for 10 min and membrane filtration using syringe filters (5 µm, 1.2 µm, 0.8 µm, and 0.45 µm; Minisart, Sartorius, Goettingen, Germany) before purification on an AKTA FPLC system (Cytiva, Marlborough, MA). The crude extract was first desalted using a HiPrep 26/10 desalting column (Cytiva) and Desalting Buffer (same as Extraction Buffer). The desalted extract was then applied to a HiTrap Q anion exchange chromatography column pre-equilibrated with Anion Exchange Binding Buffer (20 mM Tris, pH 8.0, 0.1% CHAPS). Bound proteins were eluted with increasing salt concentrations using Anion Exchange Elution Buffer (20 mM Tris, pH 8.0, 0.1% CHAPS, 1 M NaCl). Each fraction was desalted using an ultrafiltration column (10 kDa MWCO) with 10 mM MES, pH 5.6, and subsequently tested for immunogenic activity by GUS assay with the *cerk1 fls2 pCYP71A12: :GUS* line. Fractions with immunogenic activity were then mixed with Hydrophobic Interaction Binding Buffer (20 mM HEPES, pH 7.0, 2 M ammonium sulfate) and applied to a HiTrap butyl hydrophobic interaction chromatography column (Cytiva). Proteins were eluted by gradually decreasing the ammonium sulfate concentration using Hydrophobic Interaction Elution Buffer (20 mM HEPES, pH 7.0), and the immunogenic activity of each fraction was analyzed by GUS assay. Fractions with immunogenic activity were concentrated using an ultrafiltration column (10 kDa MWCO) and subjected to Superdex 200 gel filtration chromatography in Gel Filtration Buffer (20 mM Tris, pH 8.0, 150 mM NaCl). The buffer of fractions with immunogenic activity was exchanged to Cation Exchange Binding Buffer (20 mM MES, pH 5.5, 0.1% CHAPS) using an ultrafiltration column (10 kDa MWCO) and then applied to a HiTrap SP cation exchange chromatography column. The final fractions with immunogenic activity were submitted for LC-MS/MS analysis to identify proteins.

LC-MS/MS analyses

Proteins were separated by SDS-PAGE (NuPAGe ®, Invitrogen) for 5-10 min, and then the gel areas containing proteins were cut out and digested using an improved in-gel digestion method (*82*) followed by nano-LC-MS/MS using a Q Exactive LC mass spectrometer coupled to an Ultimate 3000 nano-LC system (Thermo Fisher Scientific, Waltham, MA).

RT-qPCR assay

RT-qPCR was performed as described previously (*83*). Total RNA was extracted from Arabidopsis seedlings using RNeasy Plant Mini Kit (Qiagen, Hilden, Germany) or Maxwell RSC Plant RNA Kit (Promega, WI, USA) according to the manufacturer's instructions. RNA was reverse transcribed with a ReverTraAce qPCR RT Kit (Toyobo, Osaka, Japan) according to the

manufacturer's instructions. One µg of total RNA was used as a template for cDNA synthesis. RT-qPCR was carried out using Thunderbird SYBR qPCR Mix (Toyobo) with a Stratagene Mx3000p real-time thermal cycler (Agilent, CA, USA). Relative transcript levels were calculated against a standard curve with normalization to the *U-box* housekeeping gene transcript (*At5g15400*). Primers used for the RT-qPCR are listed in Table S15.

Protein structure prediction by AlphaFold3 and comparison with yeast trehalase structure
The predicted structures of *C. elegans* and *M. incognita* trehalases were generated using AlphaFold3 with default settings via the Google Cloud-based AlphaFold Server (*34*). Model confidence was primarily assessed using the predicted TM-score (pTM). Structural comparison with the yeast neutral trehalase Nth1 bound to trehalose (PDB: 5M4A) was performed, and all models were visualized using PyMOL.

KEGG pathway analysis
KEGG pathway analysis was conducted using KEGG Mapper (https://www.genome.jp/kegg/mapper/).

Vector construction for the complementation line and CRISPR/Cas9-mediated genomic deletion lines
To generate the complementation line, *lecrk-V.5-2/pLecRK-V.5:LecRK-V.5-3×HA*, the genomic region of *LecRK-V.5* was amplified by PCR using KOD One (Toyobo). The resulting PCR products were cloned into epiGreenB5(3xHA) between the *EcoRI* and *BamHI* restriction sites using an In-Fusion HD Cloning Kit (Clontech, CA, USA). *LecRK-V.5-3×HA* in epiGreenB5 was again amplified by PCR using KOD One and the resulting PCR products were cloned into pBin19g vector between the *EcoRI* and *BamHI* restriction sites using an In-Fusion HD Cloning Kit. CRISPR/Cas9-mediated genomic deletion lines (*lecrk-V.567-d*, *lecrk-V.5-3/lecrk-V.78-d*, *lecrk-V.8/lecrk-V.56-d*) were generated using the pKAMA-ITACHI vector (pKI1.1R) as previously described (*84*). Optimal sgRNA sequences were identified using CRISPR-P (http://crispr.hzau.edu.cn/CRISPR2/), CRISPR-PLANT v2 (http://omap.org/crispr2/), CRISPOR (http://crispor.gi.ucsc.edu/), and Chopchop (https://chopchop.cbu.uib.no/). To insert an additional cassette containing the *AtU6.26* promoter, sgRNA, and polyT sequence into pKI1.1R, the cassette from pCRIPSR was amplified by PCR with FW and RV primers targeting two different sequences. The amplified fragments were inserted into the *AarI*-cut pKI1.1R vector using the In-Fusion HD Cloning Kit. Constructs were introduced into *Arabidopsis* via Agrobacterium-mediated transformation using floral dip and floral drop methods. The complementation lines of *lecrk-V.5-2/pLecRK-V.5:LecRK-V.5-3×HA (pBin19g)* were generated by direct transformation of *pLecRK-V.5:LecRK-V.5-3×HA (pBin19g)* to *lecrk-V.5-2* mutant. To select CRISPR/Cas9-mediated genomic deletion lines, T1 seeds were screened for OLE1-RFP fluorescence, and genomic deletions were confirmed by PCR. In the T2 generation, seeds lacking the OLE1-RFP signal were selected to exclude transgene-harboring plants. Primers used for sgRNA construction and genotyping are listed in Table S15.

Propagation of *M. incognita* and infection assay
M. incognita was propagated on *Solanum lycopersicum* cultivar "Micro-Tom" in a greenhouse. Nematode eggs were isolated from infected roots and hatched at 25°C. Freshly hatched J2 juveniles were collected and transferred to a Kimwipe filter placed over a glass beaker filled with sterilized

distilled water containing 100 µg/mL Streptomycin-Penicillin and 10 µg/mL nystatin overnight. Active J2s passed through the filter were collected and surface-sterilized with 0.002% mercuric chloride, 0.002% sodium azide, and 0.001% Triton X-100 for 10 minutes, followed by three rinses with distilled water (*85*). Sterilized J2s were further filtered by Kimwipes to obtain only active J2s. Arabidopsis seeds were sown and grown on half-strength MS medium with 1% sucrose and 0.8% agar under long-day conditions at 23°C. Four or five-day-old seedlings were transferred to quarter-strength MS medium with 0.5% sucrose and 0.6% phytagel pH 5.7. Twelve to thirteen-day-old seedlings were inoculated with approximately 80 nematodes per plant, and the roots were covered with black paper to simulate below-ground conditions. Gall numbers were counted six weeks after the inoculation under the microscope.

Infection assay with *Heterodera schachtii*

The *H. schachtii* infection assay was conducted following the protocol of Kranse *et al.* (*47*). Arabidopsis plants were grown under sterile conditions and inoculated with *H. schachtii* at 21 days after germination. Nematodes were hatched as described in the same protocol. Infected roots were harvested, and nematode infection numbers were counted at 14 days post-inoculation (dpi) using a stereomicroscope (Leica, GXM). To account for potential environmental variation, data were analyzed using a Best Linear Unbiased Estimators (BLUEs) model implemented in R (version 4.2.1) using the 'lme4' package. The resulting estimator values were used for pairwise comparisons using the 'emmeans' package in R. Box plots were generated using R Version 4.2.1; ggplot2 package (version 3.4.3).

ROS burst assay

Eight ten-day-old seedlings grown in 96 well plates and a solution containing 1 µM L-012 (FUJIFILM Wako, Tokyo, Japan) and 20 µg/mL horseradish peroxidase (HRP) (Sigma-Aldrich, St. Louis, MO, USA) were used. Luminescence was measured using a Tristar2 multimode reader (Berthold Technologies, Bad Wildbad, Germany).

MAPK activation assay

MAPK activation assays were performed as described previously (*86*). Ten-day-old *Arabidopsis* seedlings were flash-frozen in liquid nitrogen, and proteins were extracted using protein extraction buffer (50 mM Tris-HCl, pH 7.5, 150 mM NaCl, 10% glycerol, 2 mM EDTA, 5 mM DTT, 1x EDTA-free cOmplete Protease Inhibitor Cocktail (Merck), 0.1% IGEPAL CA630 (Merck), 0.5 mM PMSF, 1 mM Na_2MoO_4, 1 mM NaF, 0.5 mM Na_3VO_4, 20 mM β-glycerophosphate). The extract was centrifuged at 16,000 × g to remove insoluble material and protein concentration was determined by the Bradford method (Bio-Rad Laboratories, Hercules, CA). Proteins were separated by SDS-PAGE and transferred onto a PVDF membrane (Transblot, Bio-Rad Laboratories) following the manufacturer's instructions. Membranes were blocked overnight at 4°C in 5% (w/v) skim milk (FUJIFILM Wako Pure Chemical Corporation) in TBS-T. Phosphorylated MAPKs were detected using α-phospho-p44/42 MAPK (Erk1/2) (Thr202/Tyr204) rabbit monoclonal antibody (1:2000, Cell Signaling Technology, Danvers, MA) for 1 hour at room temperature in 5% (w/v) BSA (Sigma-Aldrich) in TBS-T, followed by a 1-h incubation with α-rabbit IgG-HRP secondary antibody (1:10000, Roche, Basel, Germany) in 5% skim milk in TBS-T. Detection was performed using SuperSignal West Femto Maximum Sensitivity Substrate (Thermo Fisher Scientific) with a LAS 4000 system (GE Healthcare, Chicago, IL, USA). PVDF membranes were stained with Coomassie Brilliant Blue (CBB) to confirm equal loading.

Phylogenetic analyses

The phylogenetic trees in Fig. S26 were drawn using the previously published data and visualized and pruned, and figures were generated with iTOL (*87, 88*). For alignment and phylogeny methods, refer to https://github.com/MWSchmid/Ngou-et-al.-2022. Alignment files and tree files were taken from https://doi.org/10.5281/zenodo.7017981.

Co-expression gene network analysis

Genes that co-express with *LecRK-V.5* in *Arabidopsis* were analyzed using ATTED-II (https://atted.jp) (*89*).

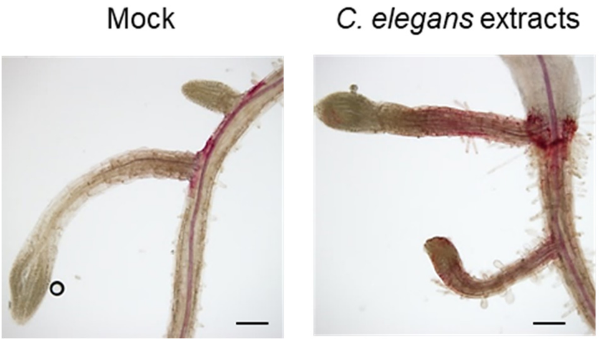

Fig. S1. ***C. elegans*** **extract induces ectopic lignin accumulation in Col-0 roots.** Col-0 seedlings were treated with 100 µg/mL *C. elegans* extract for 24 h, followed by lignin staining using phloroglucinol. Four seedlings were observed per treatment, and experiments were repeated three times with consistent results. Experiments were repeated three times with consistent results. Black bars represent 100 µm.

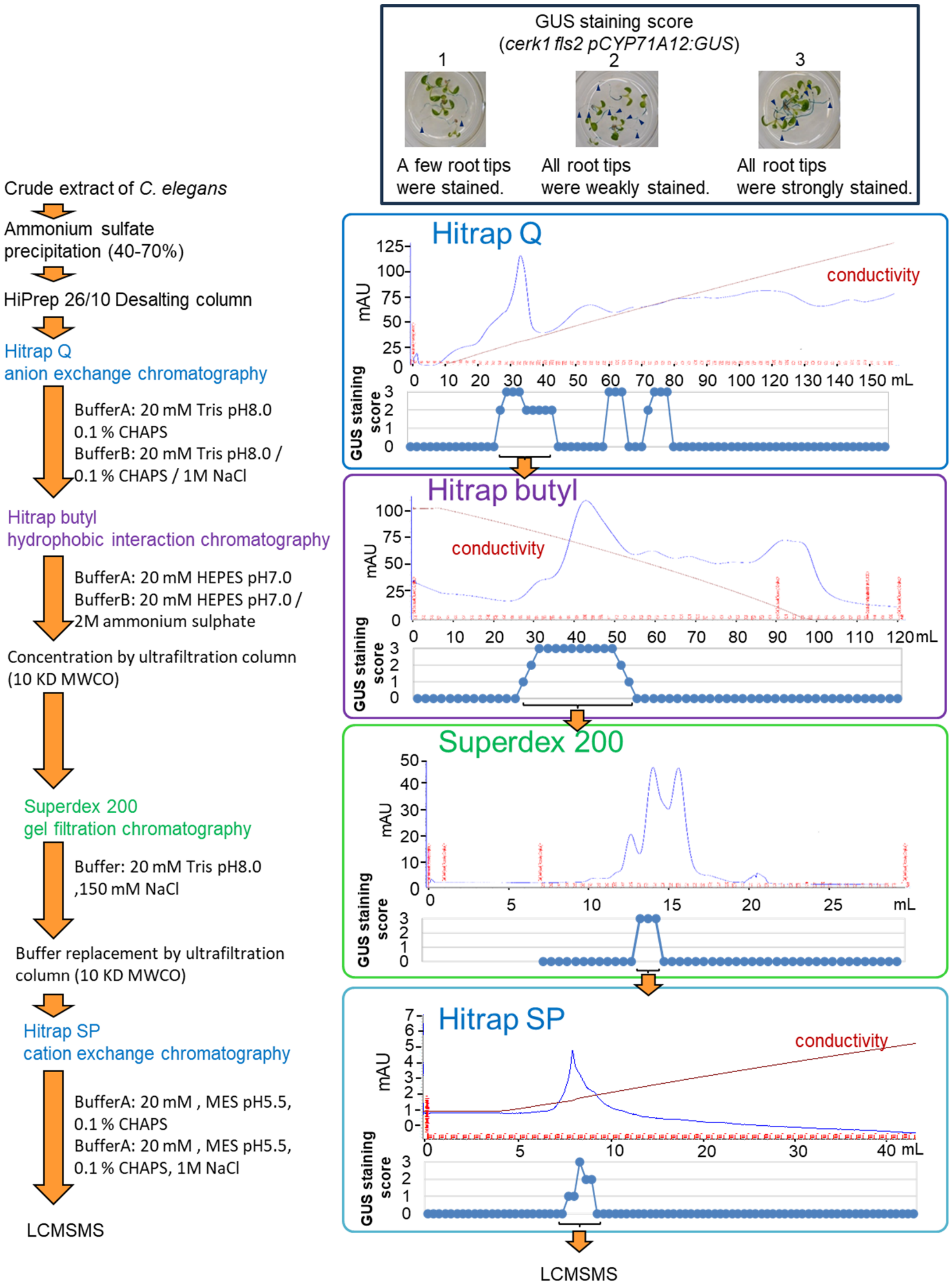

Fig. S2. Workflow for MAMP identification from *C. elegans* extract using chromatography and LC-MS/MS. MAMP activity in each fraction was assessed using the *CYP71A12* expression assay. Scores were assigned based on the number of stained root tips and staining intensities. Four to six seedlings were analyzed per treatment with each fraction. The detailed protocol is provided in the Supplementary Methods.

A

	Candidate MAMPs	Synthesized peptides
1	Trehalase (F5GUD3, G5EG27)	RYSLLYVPNSFIVPGGRFREFYYWDAYWIIKGLIASDM
2		VDKYGFVPNGGRVYYLQRSQPPFL
3		DLASAAESGWDFSTRW
4		GVFTYPGGIPTSMSQESDQQWDFPNGWSPNNHMIIEGLRKSAN
5		GGEYDVQDGFGWSNGAILDLLLTYNDRL
6	Aspartic protease (G5EEI4)	ITLGTPPQPATVVLDTGSSNLWVID
7		SWILGDTFIRQYCNVYDIGNGQIGFATAE
8	Cathepsin B (P43509)	IRDQSDCGSCWAFAAAEAISDRTCIASNG
9		DNGTPYWLVANSWNVAWGEKGYFRIIRGLNECGIEH
10		LVANSWNVAWGEKGYFRIIRGLN
11	Cathepsin B (P43508)	PYWLVANSWNVNWGENGYFRIIRGTN
12	PLB-like 3-1 (Q9BL07)	PTDPFWRQVNLTFAQLTGIYDAY
13		PGHIVTFSGYPGVLISTDDYTITSAGLTSIETTIAIFNQTLY
14		FGRYNSGTYNNQWTVLDWKQFTPEKELPD
15		YLKKYTYFASYNIPFLPKVSEISGF
16		PRARIFDRDHSKVTDIDSLTKLMRYNDYTHEEFARCKCTP
17	Neprilysin-1 (Q18673)	AFPAGILQQPFFDARFPKALNYGGIGAVIGHEITHGFDD
18	GILT-like protein (O17861)	CQHGEEECSINKFE
19	Uncharacterized protein(Q9XTU5)	PDKIKLVTLGQPRTGDYAFA

B

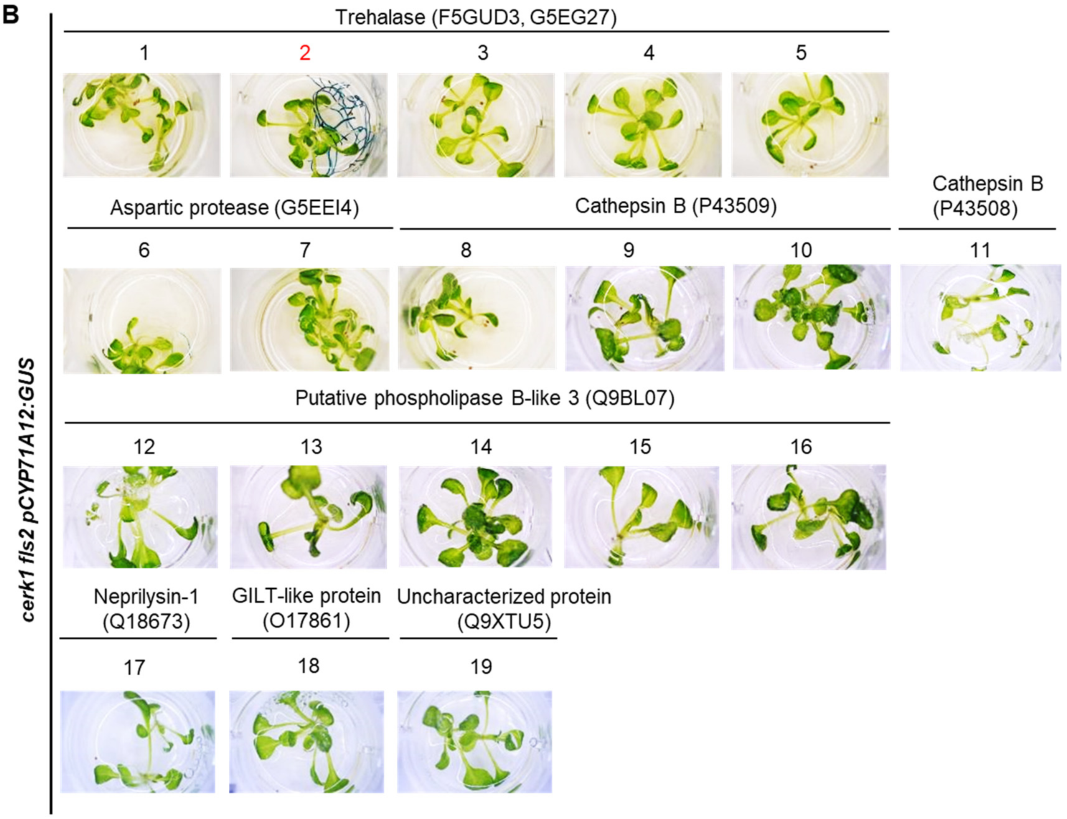

Fig. S3. Candidate MAMPs peptides. (**A**) A list of candidate MAMP peptides synthesized using highly conserved regions between *C. elegans* and plant-parasitic nematodes (PPNs). (**B**) *CYP71A12* expression in roots in *cerk1 fls2 pCYP71A12:GUS* line upon treatment with 60 µM peptides from candidate MAMPs. One to three seedlings were analyzed per treatment with each peptide. Experiments were repeated three times with consistent results. MAMP-active peptide was shown in red.

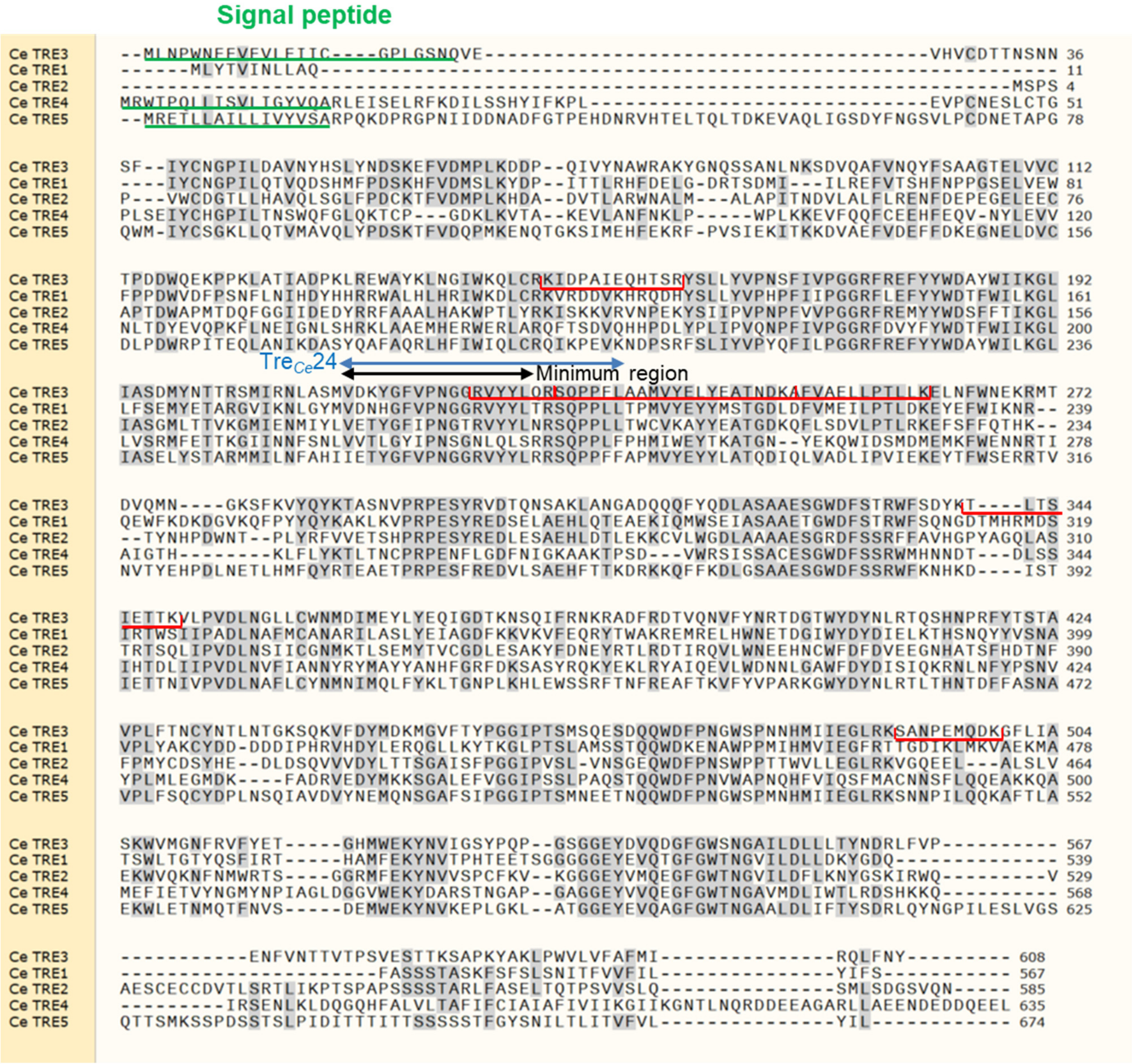

Fig. S4. Amino acid sequence alignment of trehalase proteins in *C. elegans*. Specific peptides for *Ce*TRE3 identified by LC-MS/MS are indicated with red lines. Regions corresponding to predicted signal peptides by SignalP-6.0 (https://services.healthtech.dtu.dk/services/SignalP-6.0/), Tre$_{Ce}$24, and the minimal region required for MAMP activity are also highlighted.

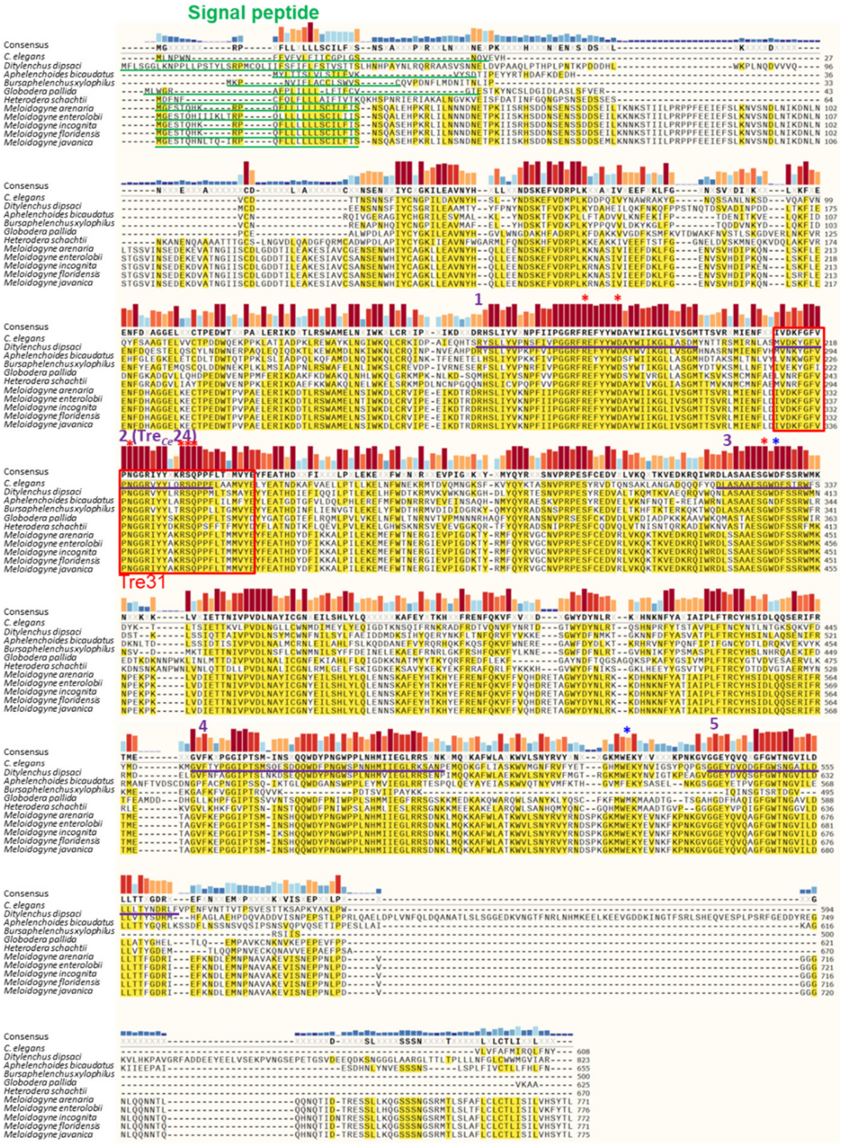

Fig. S5. Amino acid sequence alignment of secreted trehalase proteins of *C. elegans* and PPNs. Five highly conserved regions between *C. elegans* and PPNs, tested for MAMP activity, are highlighted with purple lines (see fig. S3). Regions corresponding to predicted signal peptides by SignalP-6.0 are highlighted with green lines. The 31 amino acid residues of PPNs (Tre31) shown in Fig. 1F are enclosed in a red box. The degree of conservation of each residue among sequences is displayed as colored bars above the alignment. The accession numbers of the sequences are as follows. *C. elegans* (G5EG27), *Ditylenchus dipsaci* (jg10636), *Aphelenchoides bicaudatus* (M3Y97_00305400.t1), *Bursaphelenchus xylophilus* (BXYJ5.020155000.t1), *Globodera pallida* (A0A183CKD0), *H. schachtii* (Hsc_gene_15468), *Meloidogyne arenaria* (Mare_v4_contig_148g0577361), *M. enterolobii* (MENT_LOCUS58867), *M. floridensis* (scf7180000417345.g1163), *M. incognita* (*Mi*Tre-2 (Minc_v4_shac_contig15g0140611_2)), and *M. javanica* (SRR23627520.394042). Red asterisks and blue asterisks indicate predicted substrate-binding and catalytic residues, respectively, based on structural data from yeast trehalase (PDB: 5M4A) (*27*).

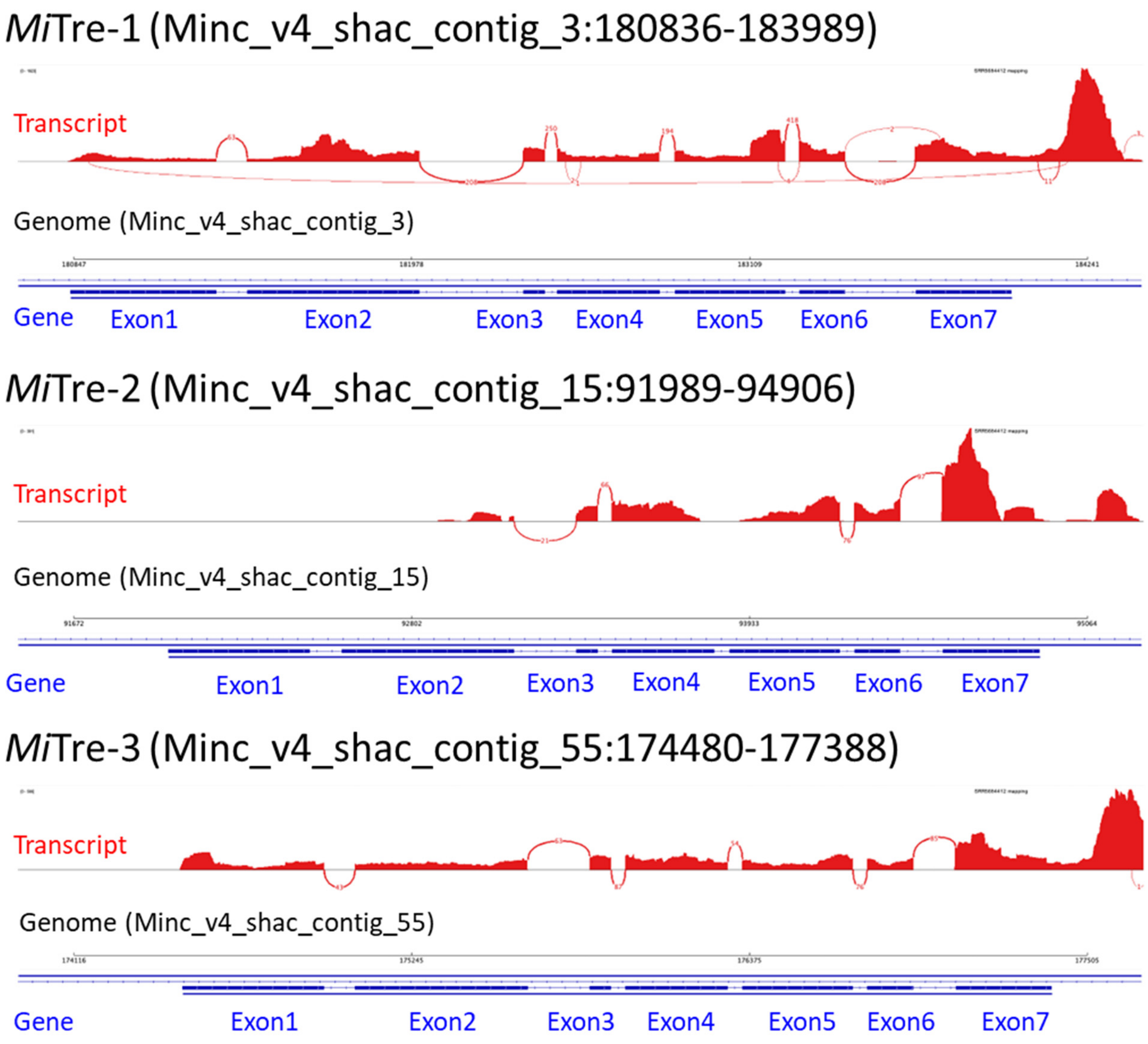

Fig. S6. The sashimi plot showing RNA-seq read mapping to the refined gene models encoding secreted trehalases (*Mi*Tre-1, *Mi*Tre-2, and *Mi*Tre-3) in *M. incognita*. The mapping results for J2 stage nematodes (SRR5684412) are shown (*72*). Only introns ≤ 4 kb are displayed. Sashimi plot was obtained by Integrative Genomics Viewer (IGV) version 2.18.1 (*90*). The y-axes of mapping coverage are not shown to the same scale.

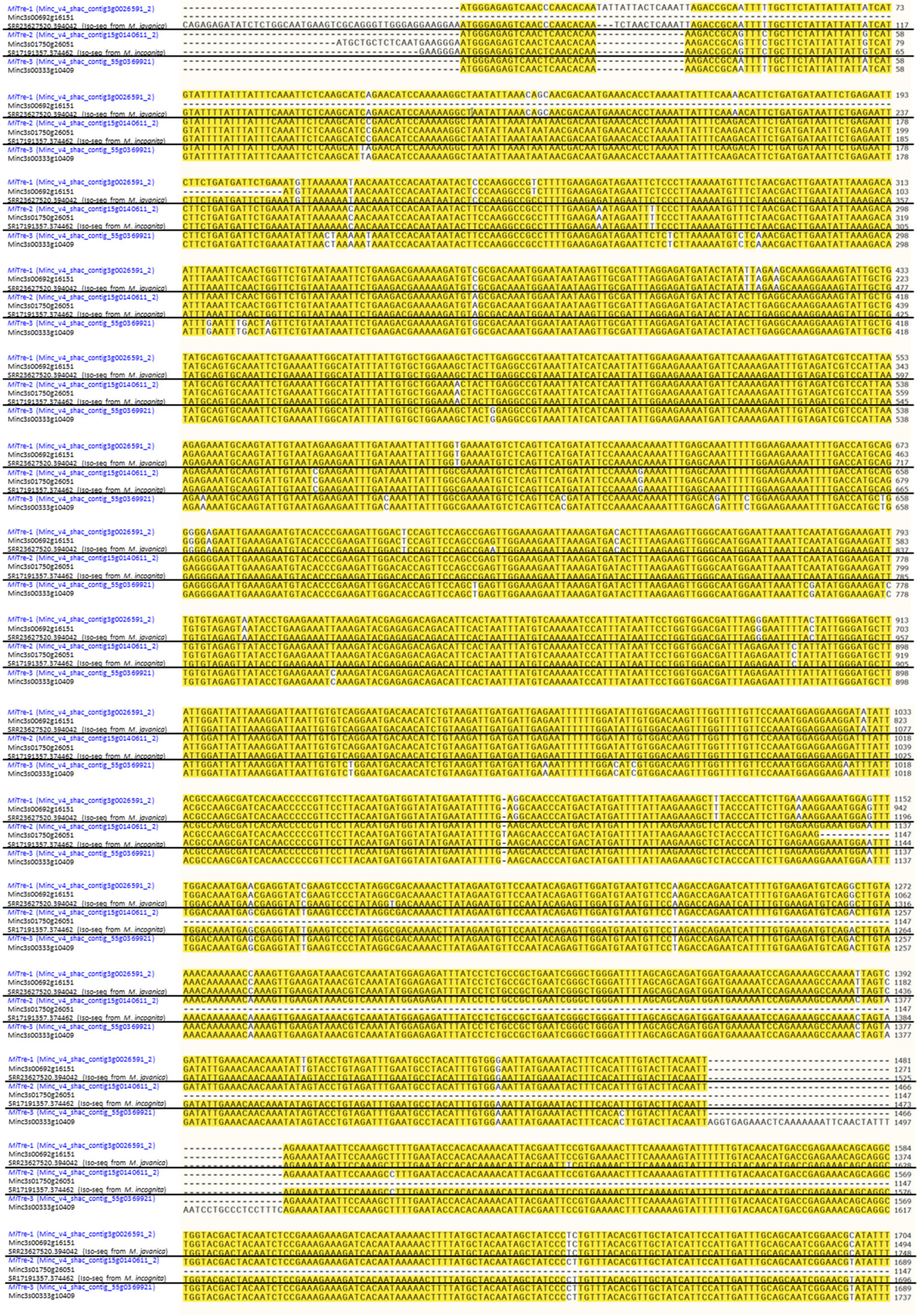

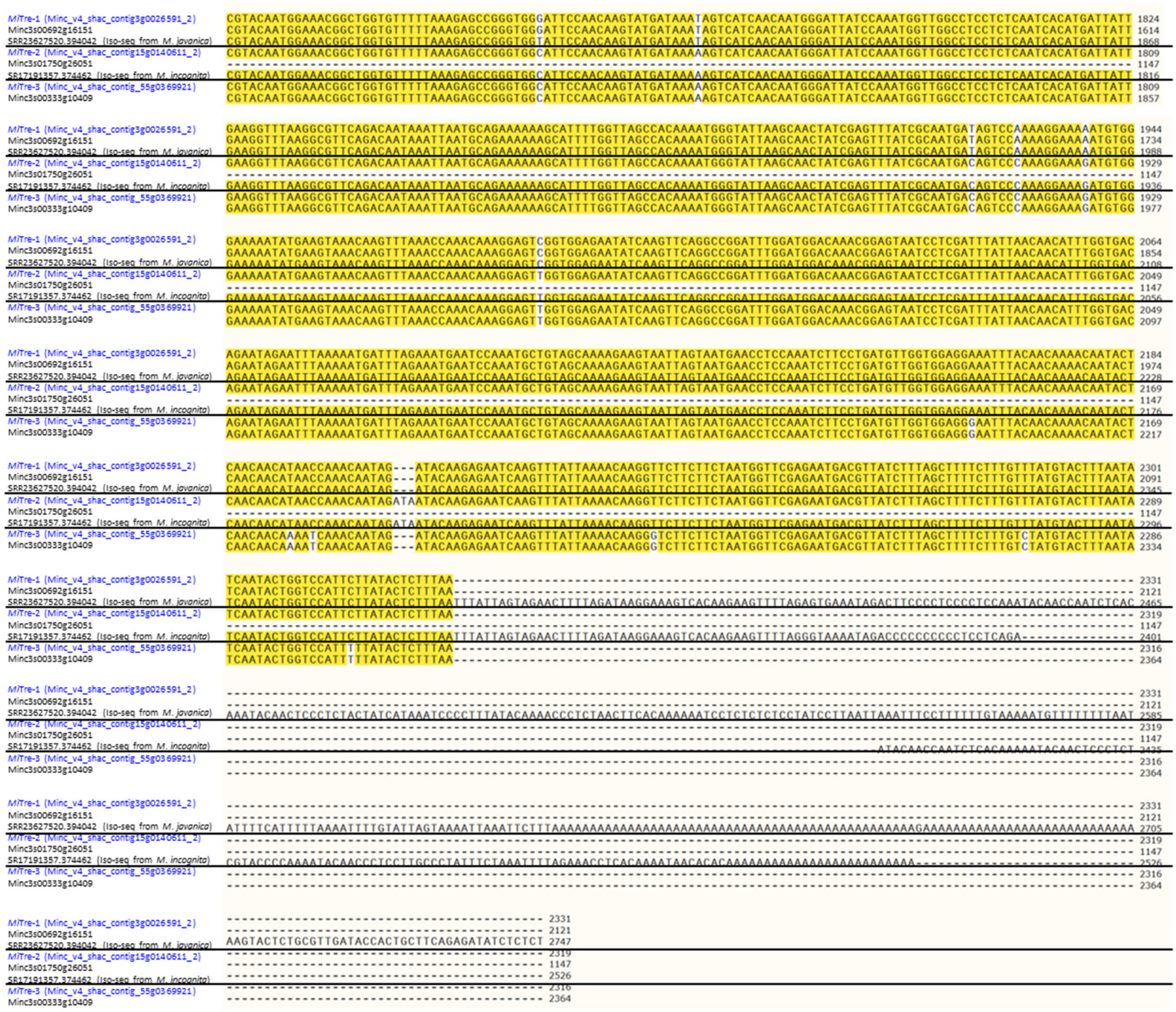

Fig. S7. Alignment of refined transcript models and previous annotations encoding secreted trehalases in *M. incognita*, along with Iso-Seq transcripts from *M. incognita* and *M. javanica*. Through manual refinement of gene models, we identified three genes encoding secreted trehalases that contain both an N-terminal signal peptide and the MAMP-active sequence (*Mi*Tre-1, *Mi*Tre-2, and *Mi*Tre-3). This figure shows the alignment of transcripts corresponding to *Mi*Tre-1, *Mi*Tre-2, and *Mi*Tre-3, together with previously annotated transcripts (Minc3s00692g1615, Minc3s01750g26051, and Minc3s00333g10409) and Iso-Seq transcripts from *M. incognita* (SR17191357.374462) (*68*) and (SRR23627520.394042) *M. javanica* (*71*) as reported in earlier studies.

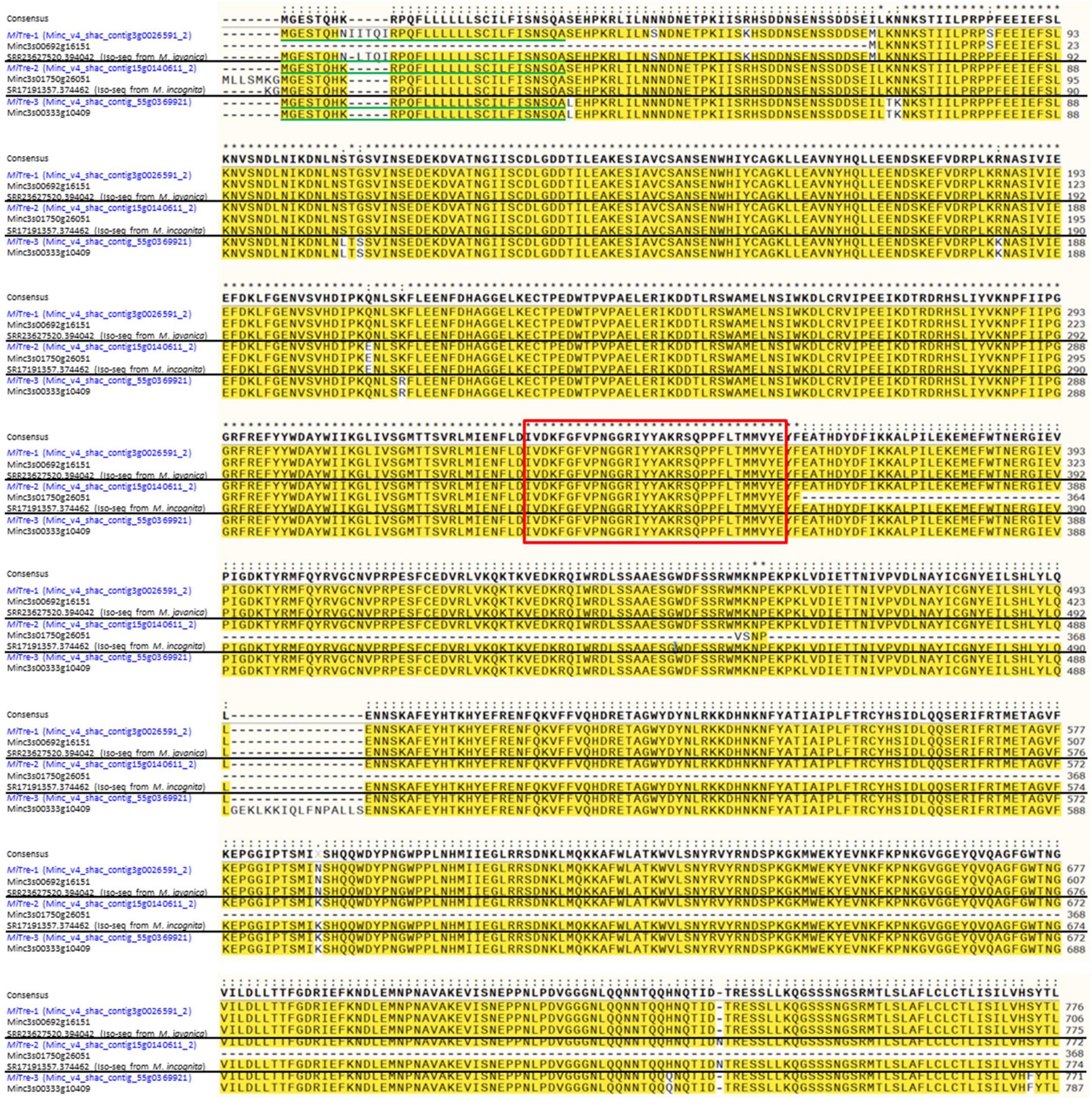

Fig. S8. Alignment of refined protein sequence models and previously annotated sequences of secreted trehalases in *M. incognita*, along with protein sequences derived from Iso-Seq transcripts from *M. incognita* and *M. javanica*. Regions corresponding to predicted signal peptides by SignalP-6.0 are highlighted with green lines. The 31 amino acid residues of Tre$_{Mi}$31 shown in Fig. 1F are enclosed in a red box.

A

cerk1 fls2 pCYP71A12:GUS

Name	Sequence	10 µM	30 µM	50 µM
Tre$_{Ce}$32	VDKYGFVPNGGRVYYLQRSQPPFLAAMVYELY			
Tre$_{Ce}$26	VDKYGFVPNGGRVYYLQRSQPPFLAA			
Tre$_{Ce}$24	VDKYGFVPNGGRVYYLQRSQPPFL			
Tre$_{Ce}$23	VDKYGFVPNGGRVYYLQRSQPPF			
Tre$_{Ce}$21	VDKYGFVPNGGRVYYLQRSQP			
Tre$_{Ce}$20	VDKYGFVPNGGRVYYLQRSQ			
Tre$_{Ce}$19	VDKYGFVPNGGRVYYLQRS			
Tre$_{Ce}$18	VDKYGFVPNGGRVYYLQR			
Tre$_{Ce}$17	VDKYGFVPNGGRVYYLQ			
Tre$_{Ce}$16	VDKYGFVPNGGRVYYL			
Tre$_{Ce}$15	VDKYGFVPNGGRVYY			

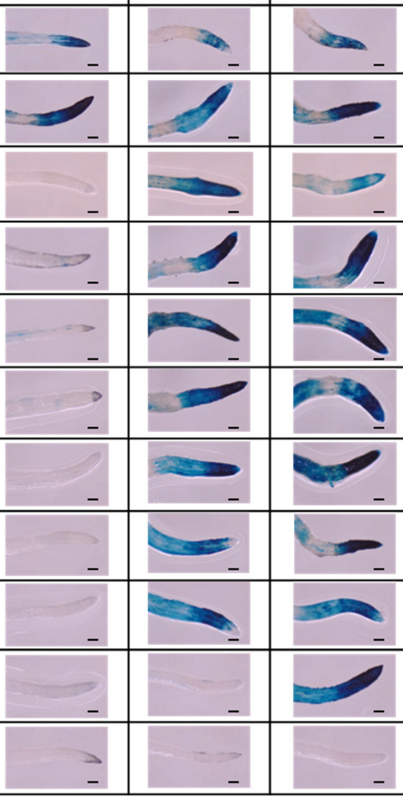

B

cerk1 fls2 pCYP71A12:GUS

Name	Sequence	10 µM	30 µM	50 µM
Tre$_{Ce}$24-2	KYGFVPNGGRVYYLQRSQPPFL			
Tre$_{Ce}$24-4	GFVPNGGRVYYLQRSQPPFL			
Tre$_{Ce}$24-6	VPNGGRVYYLQRSQPPFL			
Tre$_{Ce}$24-8	NGGRVYYLQRSQPPFL			

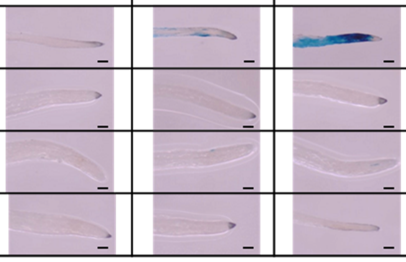

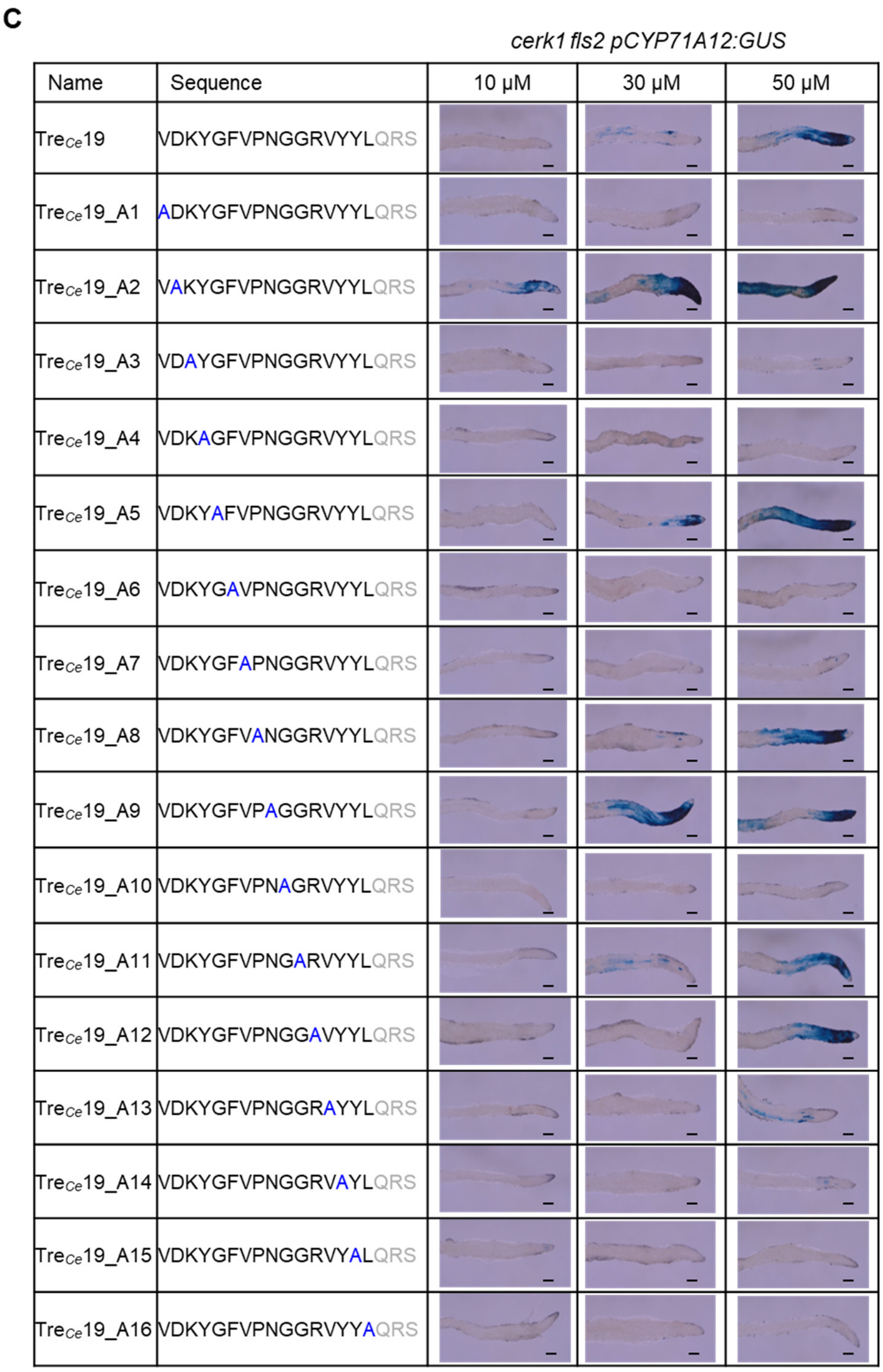

Fig. S9. Minimal region and essential residues for MAMP activity of the *C. elegans* trehalase peptide. (**A**) MAMP activity of C-terminally truncated trehalase peptides and Tre*Ce*24 with hydroxylated prolines, determined via the *CYP71A12* expression assay using *cerk1 fls2 pCYP71A12:GUS* line. (**B**) MAMP activity of N-terminally truncated trehalase peptides. (**C**) Alanine scanning of the Tre*Ce*19 peptide. Black bars represent 100 µm. Four to six seedlings were analyzed per treatment. Experiments were repeated three times with consistent results.

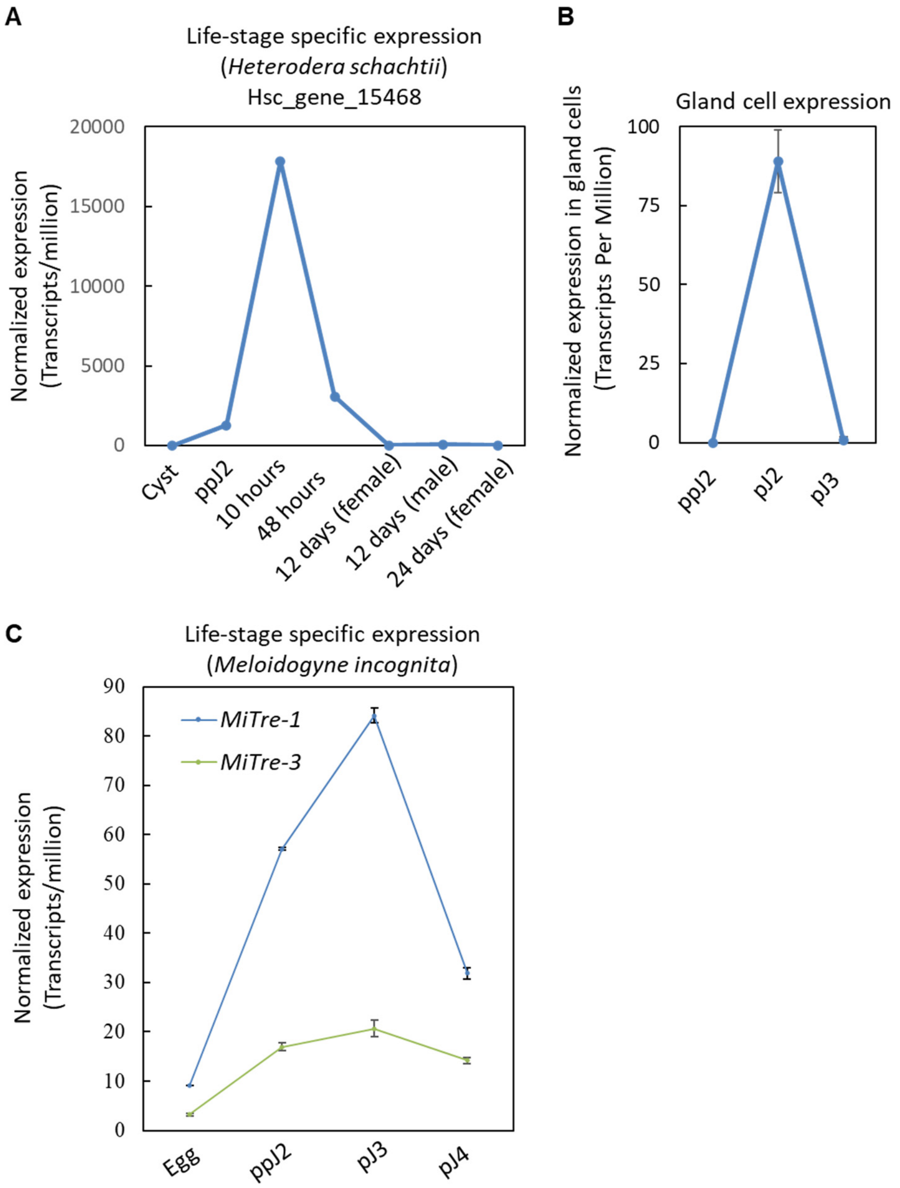

Fig. S10. The transcript levels of secreted trehalases in *H. schachtii* and *M. incognita* during infection. (**A**) Life-stage specific expression of Hsc_gene_15468 in *H. schachtii* based on previously published studies (*30-32*). Samples include cysts, pre-parasitic second-stage juveniles (ppJ2), and infected *Arabidopsis thaliana* root segments collected at various time points: 10 hours post infection (hpi; migratory parasitic J2s [pJ2], pre-feeding site establishment), 48 hpi (post-feeding site establishment), 12 days post-infection (dpi) females (virgin), 12 dpi males (differentiated, pre-emergence, mostly no longer feeding), and 24 dpi females (post-mating). (**B**) Expression of *Hsc_gene_15468* in subventral gland cells of *H. schachtii* at the ppJ2, pJ2, and pJ3 stages. Values are presented as mean ± standard diviasion (SD) (n=3). (**C**) Life stage-specific expression of *MiTre-1* and *MiTre-3* in *M. incognita* at the egg, ppJ2, pJ3, and pJ4 stages, based on previously published data (*33*). Values are presented as mean ±SD (n=3).

A

cerk1 fls2 pCYP71A12:GUS

Name		50 µM	100 µM
Tre *Ditylenchus dipsaci* (jg10636)	MVNKYGFVPNGGRVYYLRRSQP	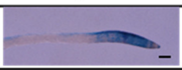	
Tre *Aphelenchoides bicaudatus* (M3Y97_00305400.t1)	LVNKWGFVPNGGRIYYLARSQP	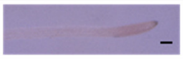	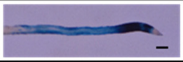
Tre *Bursaphelenchus xylophilus* (BXYJ5.020155000.t1)	IVEKYGFIPNGGRVYYLTRSQP	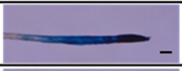	
Tre *Globodera pallida* (A0A183CKD0)	LVNRFGFVPNGGRIYYSKRSQP	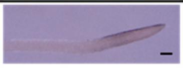	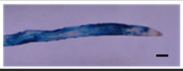
Tre *Heterodera schachtii* (Hsc_gene_15468)	MVNRFGFVPNGGRIYYDKRSQP	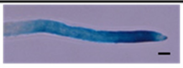	

B

cerk1 fls2 pCYP71A12:GUS

Name	Sequence	10 µM	30 µM	50 µM
Tre$_{Mi}$31	IVDKFGFVPNGGRIYYAKRSQPPFLTMMVYE	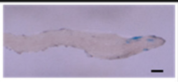	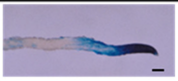	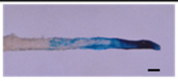
Tre$_{Mi}$16	VDKFGFVPNGGRIYYA	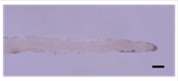	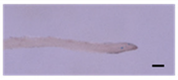	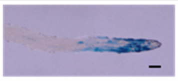

Fig. S11. MAMP activity of trehalase peptides from plant parasitic nematodes. (**A**) MAMP activity of trehalase peptides from plant parasitic nematodes, assessed via the *CYP71A12* expression assay using *cerk1 fls2 pCYP71A12:GUS* line. (**B**) MAMP activity of Tre$_{Mi}$16 and Tre$_{Mi}$31 peptide. Black bars represent 100 µm. Four to six seedlings were analyzed per treatment. Experiments were repeated three times with consistent results.

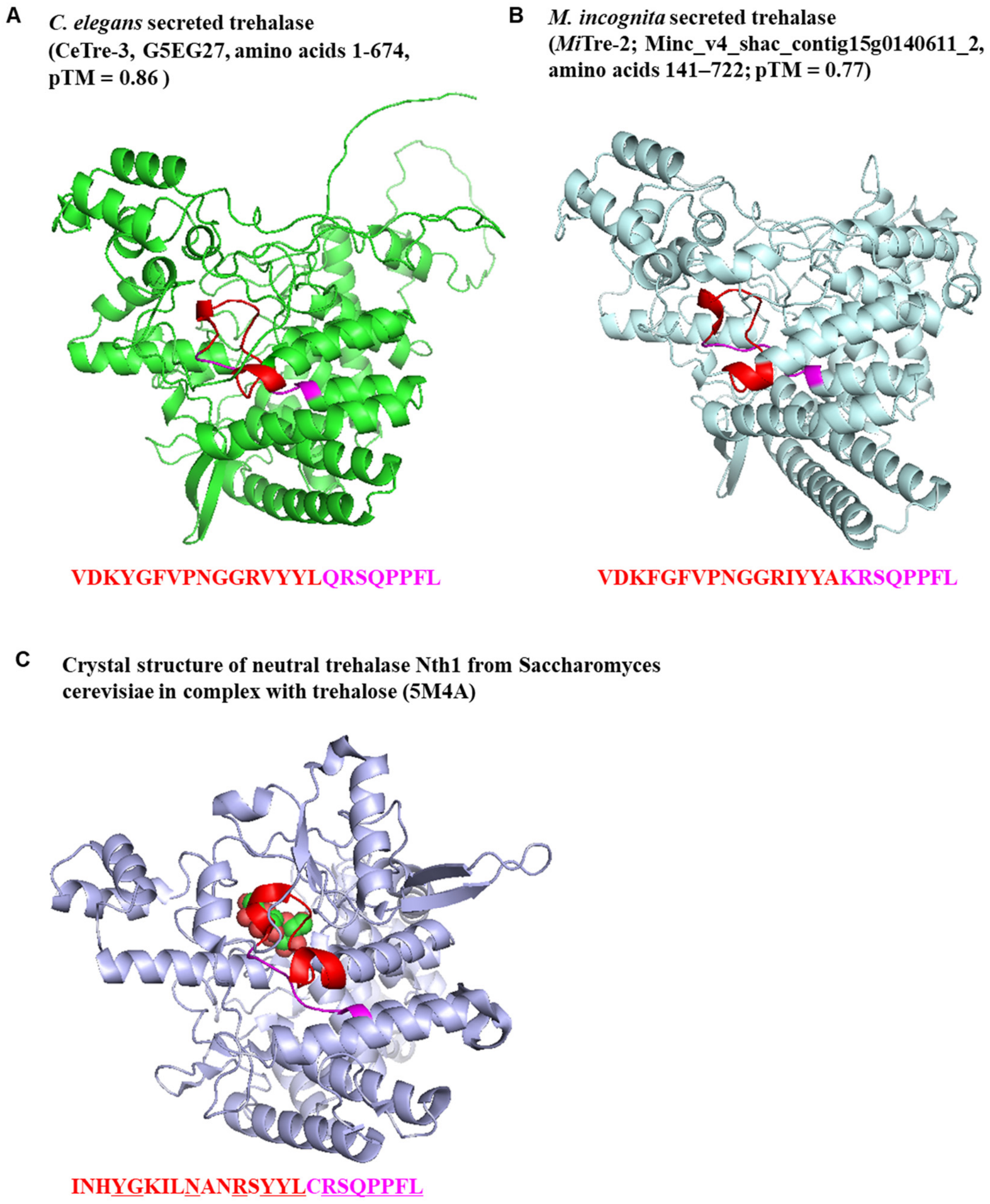

Fig. S12 Protein structure prediction by AlphaFold3 and comparison with yeast trehalase structure. (**A**, **B**) The predicted structures of *C. elegans* and *M. incognita* trehalases were generated using AlphaFold3 with default settings. Model confidence was primarily assessed using the predicted TM-score (pTM). (**C**) Crystal structure of yeast neutral trehalase Nth1 bound to trehalose (PDB: 5M4A). The minimal region required for MAMP activity in the Tre$_{Ce}$ peptide and the corresponding residues in *M. incognita* and yeast trehalase are highlighted in red; the C-terminal extension and corresponding residues are shown in pink. Residues in the yeast trehalase that are identical to those in Tre$_{Ce}$ peptide are underlined.

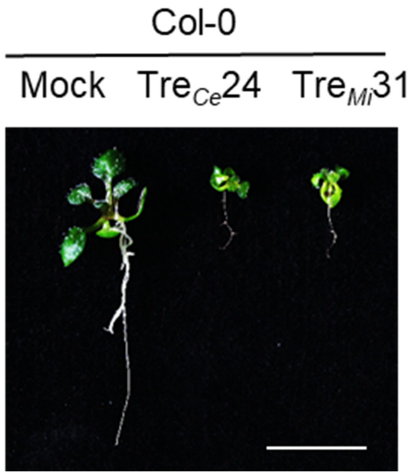

Fig. S13. Characterization of trehalase peptide-induced responses. 50 µM $Tre_{Ce}24$ and $Tre_{Mi}31$ induce seedling growth inhibition in Col-0. The white bar represents 1 cm. Four seedlings were analyzed per treatment. Experiments were repeated three times with consistent results.

A **Phe, Tyr, and Trp biosynthesis**

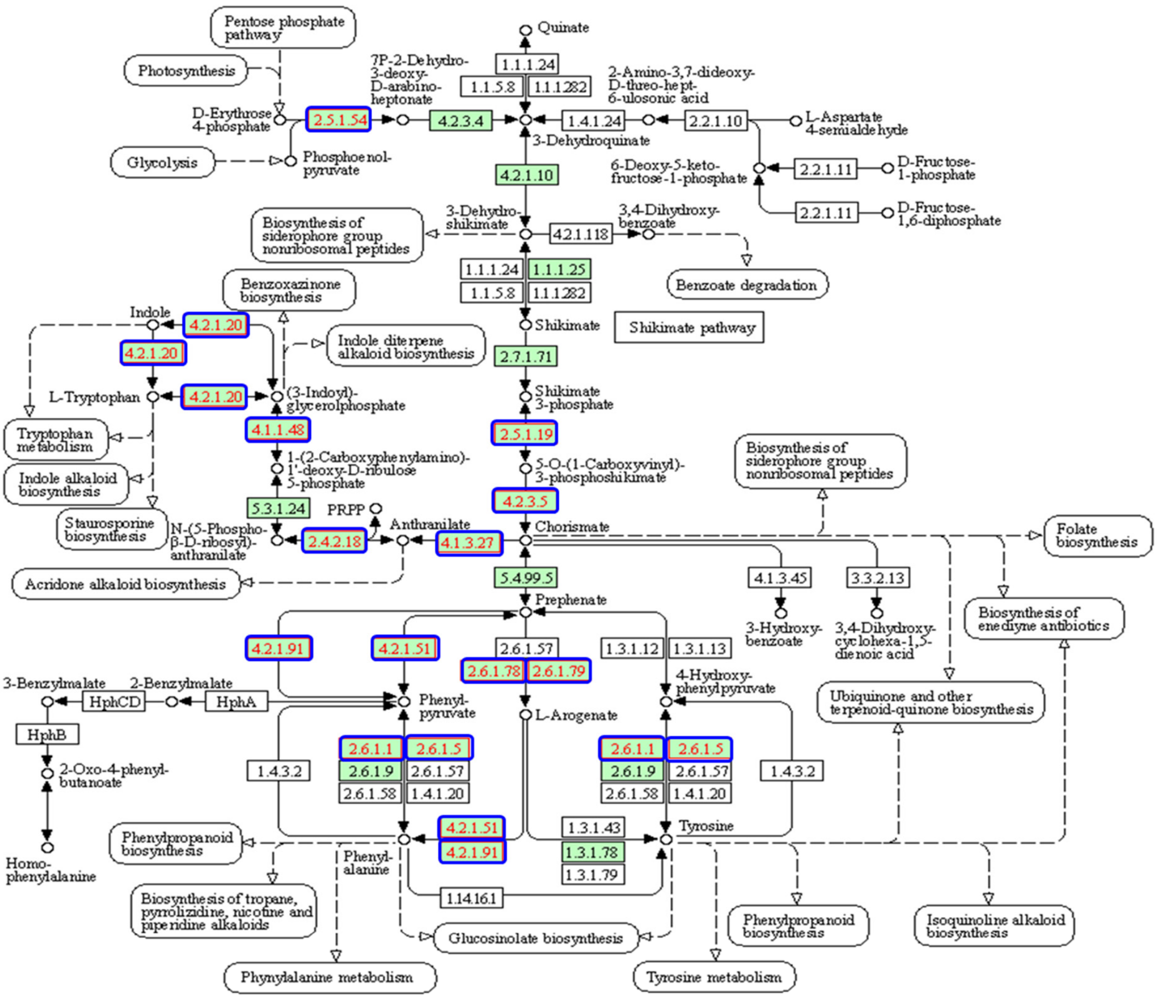

Expression upon Tre$_{Mi}$31 treatment for 12 hours

	Gene ID	logFC	adj_ pvalue
Class-II DAHP synthetase	AT1G22410	2.07	2.64E-20
DHS1	AT4G39980	1.87	1.42E-40
TSA1	AT3G54640	3.41	1.79E-145
IGPS	AT2G04400	2.98	7.49E-88
EPSP synthase	AT2G45300	1.61	1.31E-18
chorismate synthase	AT1G48850	1.56	5.56E-25
Glutamine amidotransferase	AT1G24807	3.51	2.44E-38
ADT4	AT3G44720	1.98	2.61E-18
AAT	AT2G22250	1.64	3.95E-18
Tyrosine transaminase	AT4G28420	8.85	2.36E-19

B **Phenylpropanoid biosynthesis**

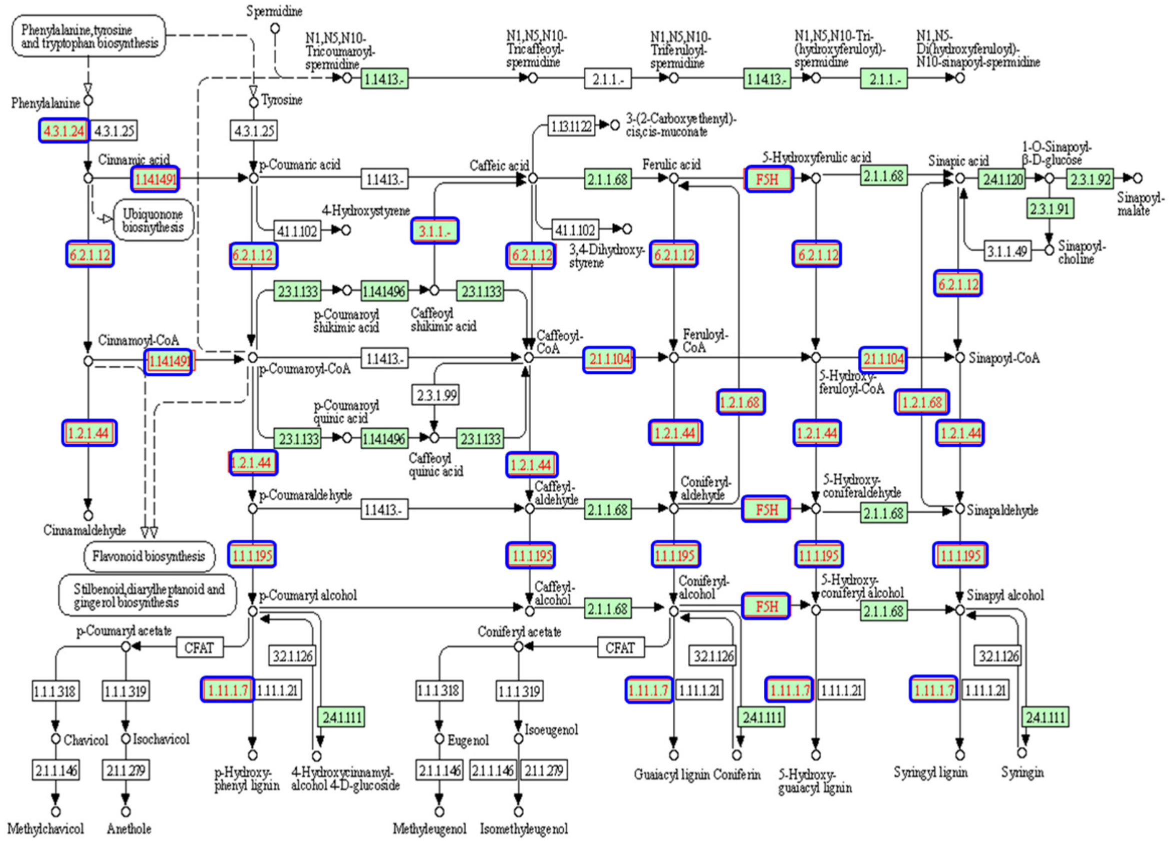

Expression upon $Tre_{Mi}31$ treatment for 12 hours

	Gene ID	logFC	adj_pvalue
PAL	AT2G37040	1.84	5.32E-15
C4H	AT2G30490	1.73	5.16E-20
F5H,FAH1	AT4G36220	1.10	3.31E-07
4CL	AT1G51680	1.83	6.56E-20
LysoPL2	AT1G52760	1.09	1.35E-11
cinnamate-4-hydroxylase	AT2G30490	1.73	5.16E-20
CCOAMT	AT1G67980	8.94	4.28E-169
CCR2	AT1G80820	3.10	4.05E-22
ALDH2C4	AT3G24503	1.75	2.32E-29
CAD	AT4G37990	2.94	8.01E-06
FAH1	AT4G36220	1.10	3.31E-07
Peroxidase	AT1G14540	7.98	1.27E-65

C

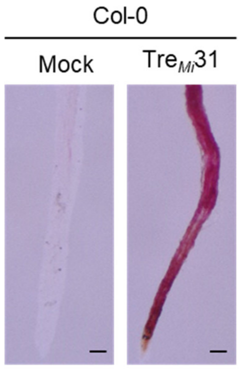

D

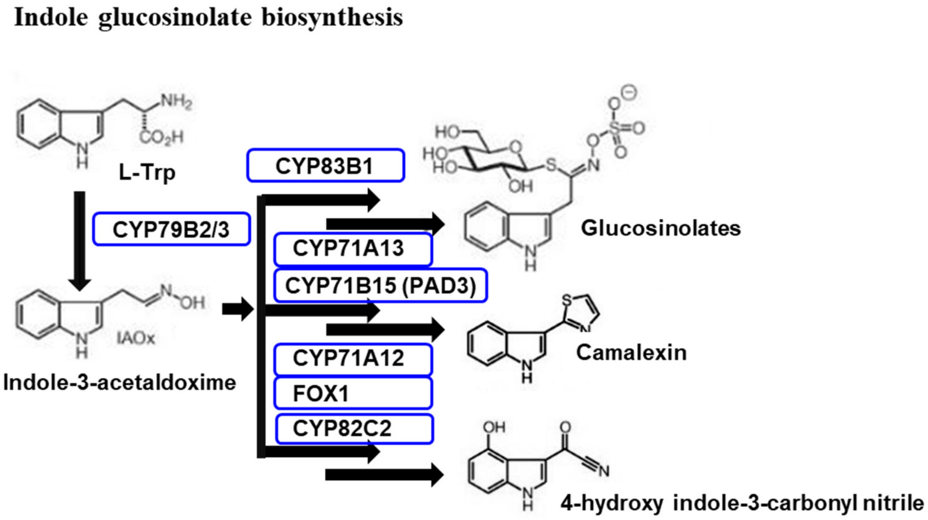

Expression upon Tre$_{Mi}$31 treatment for 12 hours

	Gene ID	logFC	adj_pvalue
CYP79B2	AT4G39950	3.38	6.19E-42
CYP83B1	AT4G31500	2.10	2.06E-34
CYP71A13	AT2G30770	6.44	2.57E-82
CYP71B15 (PAD3)	AT3G26830	6.82	7.67E-121
CYP71A12	AT2G30750	9.13	2.99E-196
FOX1	AT1G26380	9.09	9.26E-165
CYP82C2	AT4G31970	10.71	3.91E-52

Fig. S14. Pathway analysis of genes specifically upregulated upon treatment with Tre$_{Mi}$31. **(A and B)** KEGG (Kyoto Encyclopedia of Genes and Genomes) pathway analysis of genes involved in phenylalanine, tyrosine, and tryptophan biosynthesis (**A**) and phenylpropanoid biosynthesis (**B**), whose expressions are specifically induced by the treatment with 30 µM Tre$_{Mi}$31 for 12 hours (Table S7). Enzymes with expression induced by Tre$_{Mi}$31 are shown in blue boxes. (**C**) Tre$_{Mi}$31 induces lignin accumulation in Col-0 seedlings. Lignin was stained using phloroglucinol upon treatment with 50 µM Tre$_{Mi}$31 for 3 days. Four seedlings were analyzed per treatment. Experiments were repeated three times with consistent results. Black bars represent 100 µm. (**D**) Pathway analysis of genes involved in indole glucosinolate biosynthesis whose expressions are specifically induced by the treatment with 30 µM Tre$_{Mi}$31 for 12 hours. Enzymes with expression induced by Tre$_{Mi}$31 are shown in blue boxes.

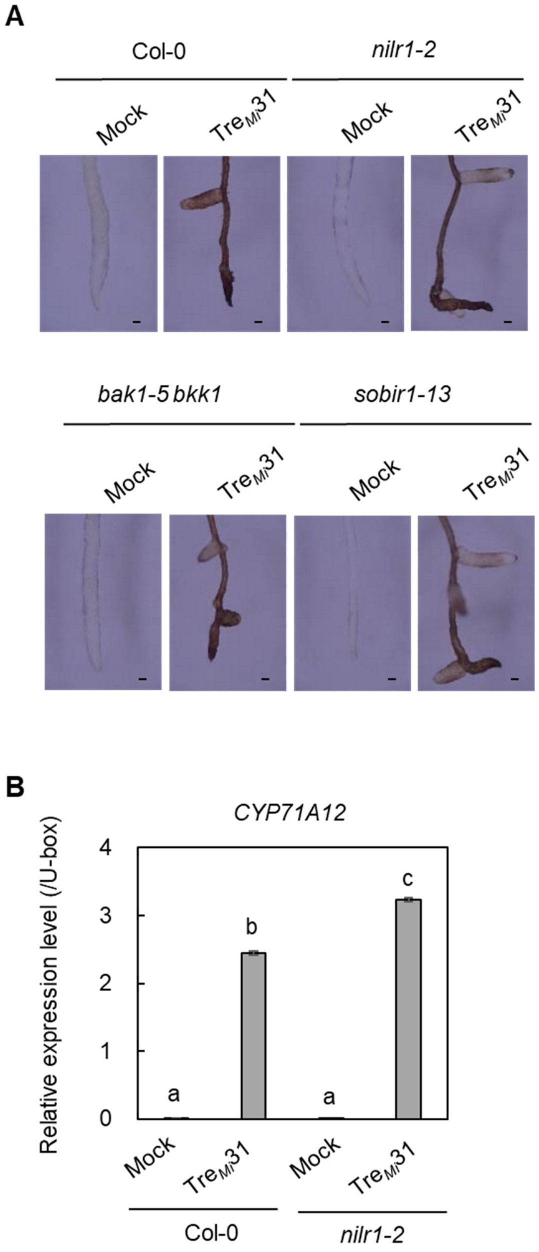

Fig. S15. NILR1, BAK1, and SOBIR1 are not required for Tre$_{Mi}$31-induced responses. (**A**) *nilr1-2*, *bak1-5 bkk1*, and *sorbir1-13* mutants induce root pigmentation similar to Col-0 upon treatment with 50 µM Tre$_{Mi}$31. Four seedlings were analyzed per treatment. Black bars represent 100 µm. **(B)** *nilr1-2* mutant induces *CYP71A12* expression similar to Col-0 upon treatment with 50 µM Tre$_{Mi}$31 for 6 hours. Transcript levels of *CYP71A12* in the seedlings were measured by RT-qPCR after normalization to the *U-box* housekeeping gene transcript (*AT5G15400*). Values are presented as mean ± standard error (SE) of three technical replicates, with different letters indicating significant differences ($P \leq 0.0001$, one-way ANOVA with Tukey's post hoc test). All the experiments were repeated three times with consistent results.

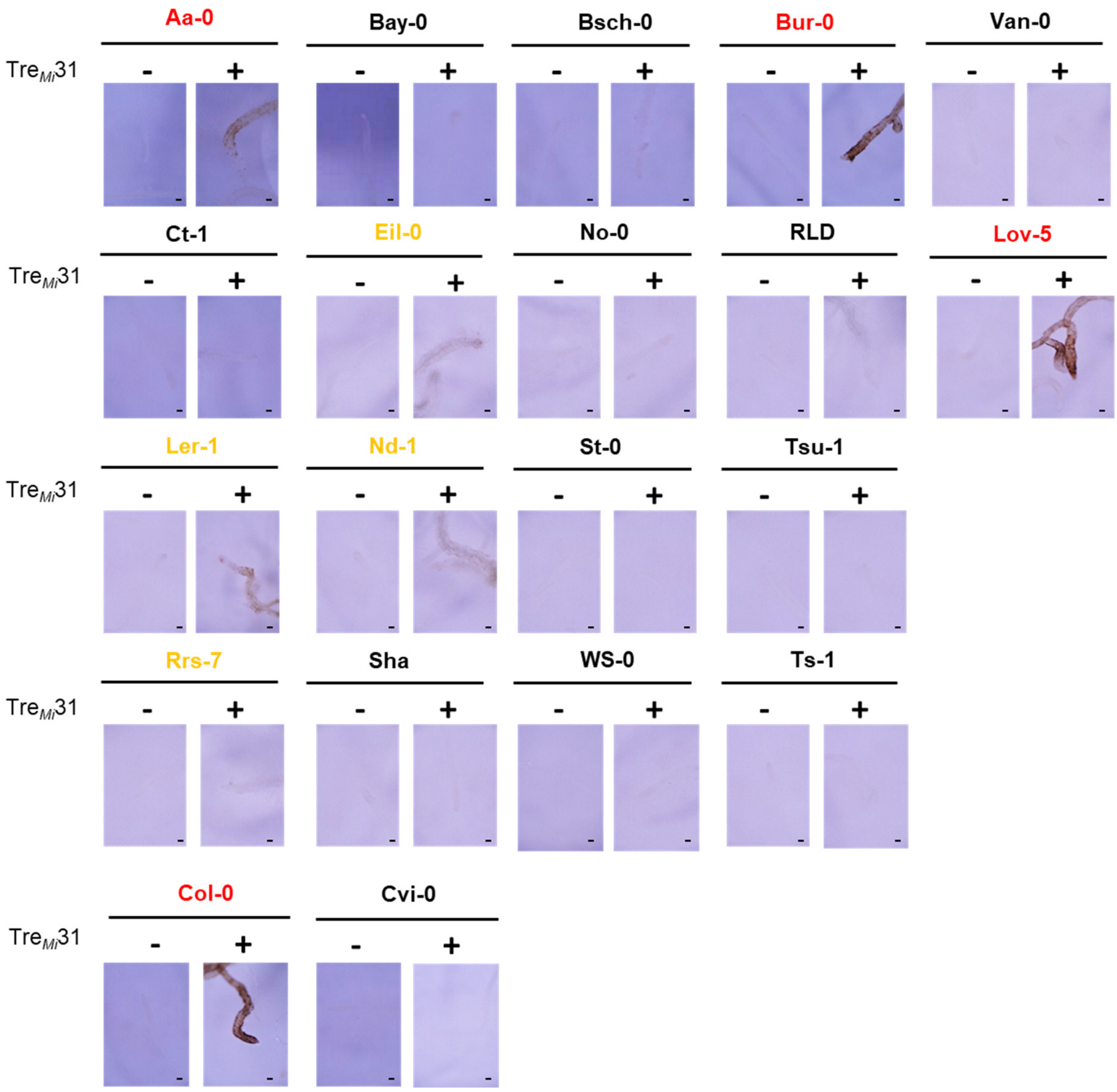

Fig. S16. Variation in root pigmentation induced by 50 µM Tre$_{Mi}$31 across different *Arabidopsis* accessions. Four seedlings were analyzed per treatment. Black bars represent 100 µm. Highly sensitive accessions are indicated in red, while moderately sensitive accessions are shown in yellow.

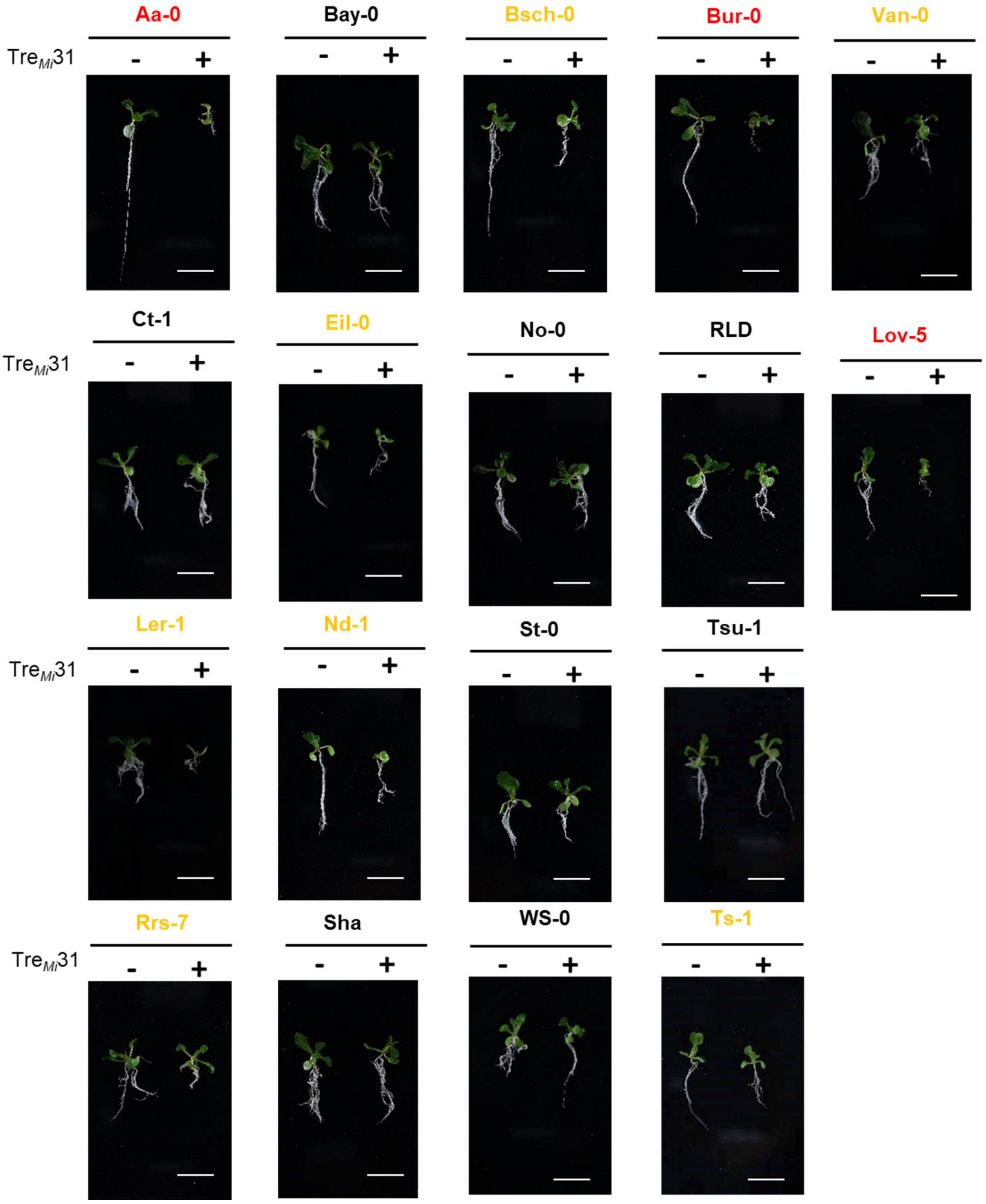

Fig. S17. Variation in seedling growth inhibition induced by 50 µM Tre$_{Mi}$31 across different Arabidopsis accessions. Four seedlings were analyzed per treatment. White bars represent 1 cm. Highly sensitive accessions are indicated in red, while moderately sensitive accessions are shown in yellow.

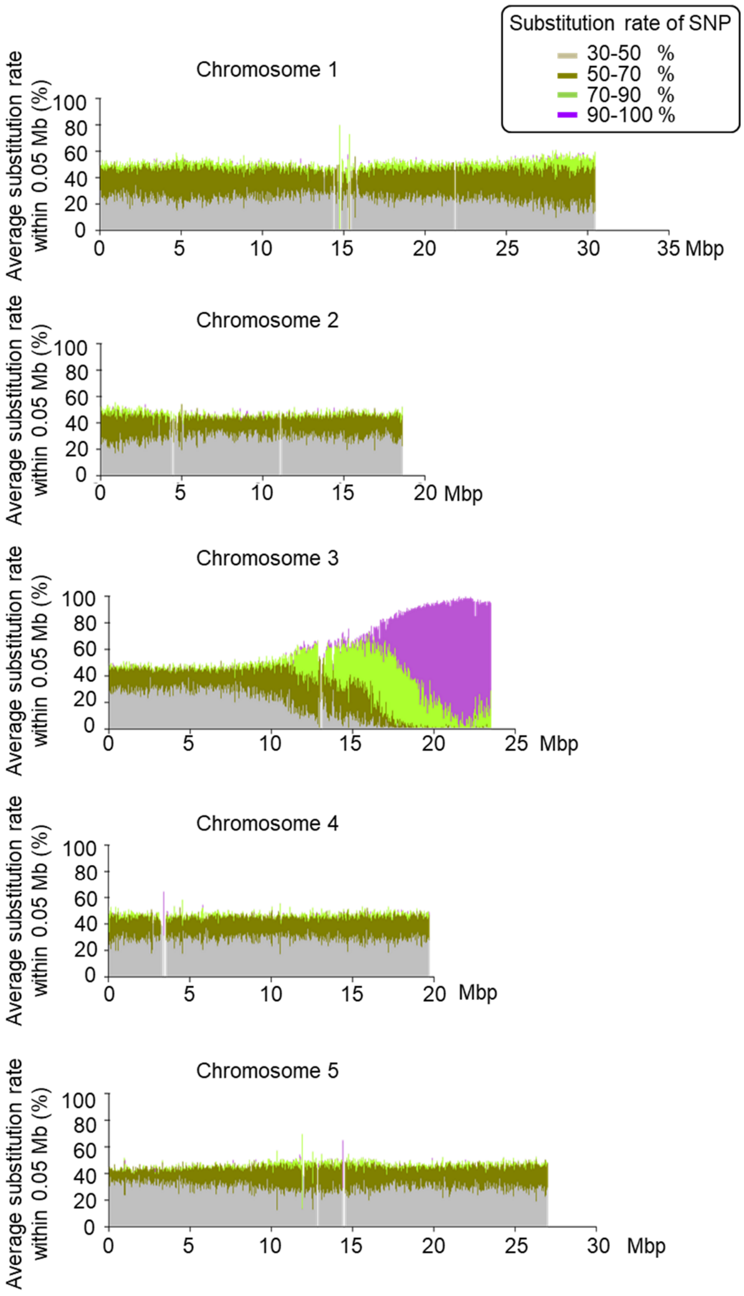

Fig. S18. Mapping of the chromosomal region required for Tre$_{Mi}$31 sensitivity. Genomic sequencing of F2 populations with insensitivity from Col-0 × Cvi-0 crosses identified a genomic region associated with Tre$_{Mi}$31 insensitivity in Cvi-0. Significant accumulations of SNPs from Cvi-0 were detected near the end of chromosome 3. The substitution ratio of SNPs (Col-0 to Cvi-0) was categorized into four levels (30-50%, 50-70%, 70-90%, and 90-100%) and color-coded accordingly. The vertical axis represents the average substitution ratio of SNPs (Cvi-0 / (Col-0 + Cvi-0)) within a 0.05 Mb window.

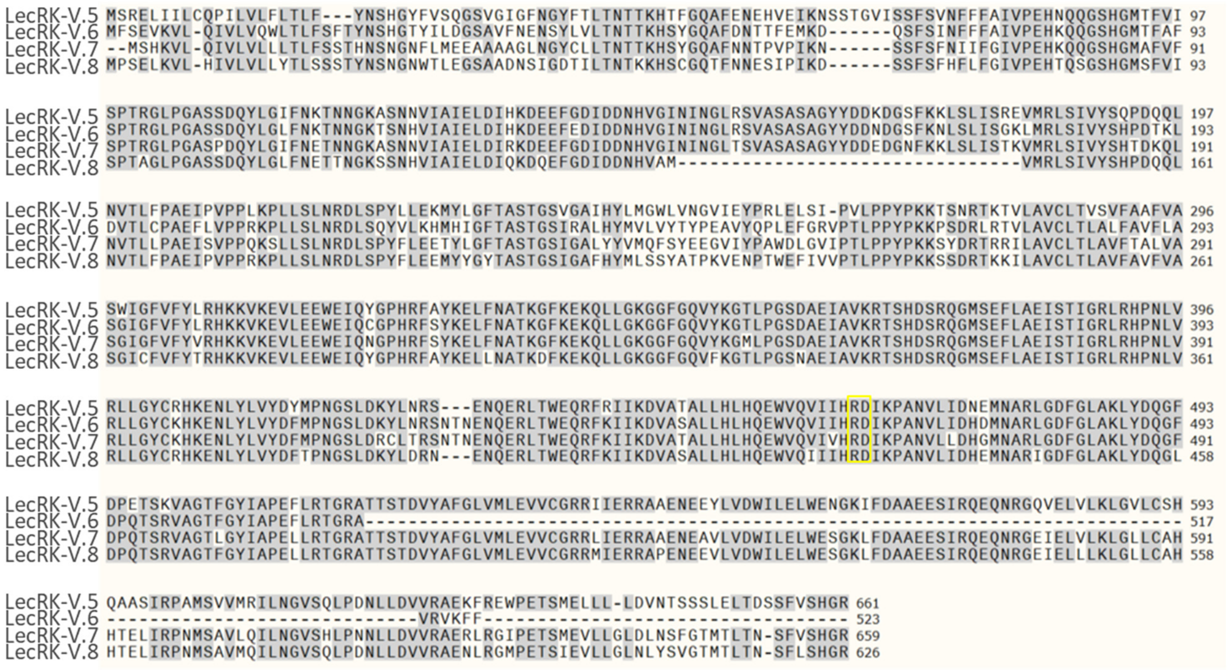

Fig. S19. Amino acid sequence alignment of LecRK-V.5, V.6, V.7, and V.8. The conserved arginine (R) and aspartic acid (D) in the catalytic loop are shown in the yellow box.

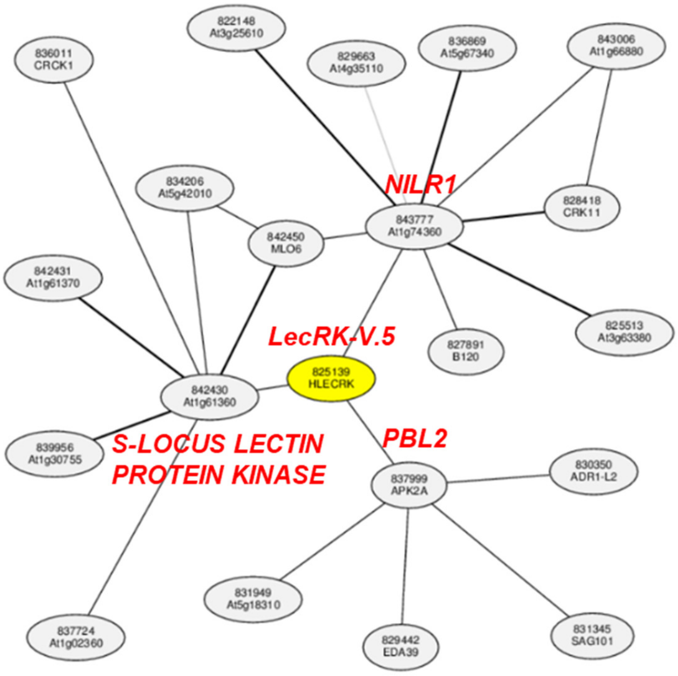

Genes directly connected with *LecRK-V.5* on the network in ATTED-II

Coexpression z score	Locus	Function
6.9	AT1G74360	NILR1 (NEMATODE-INDUCED LRR-RLK 1), Leucine-rich repeat protein kinase family protein
6.9	AT1G61360	S-locus lectin protein kinase family protein
6.1	AT1G14370	protein kinase 2A/PBL2 (PBS1-LIKE 2),

Fig. S20. *LecRK-V.5* co-expressed with *NILR1*. Co-expression analysis of *LecRK-V.5* using ATTED-II shows that *NILR1* is the most co-expressed gene in *Arabidopsis*.

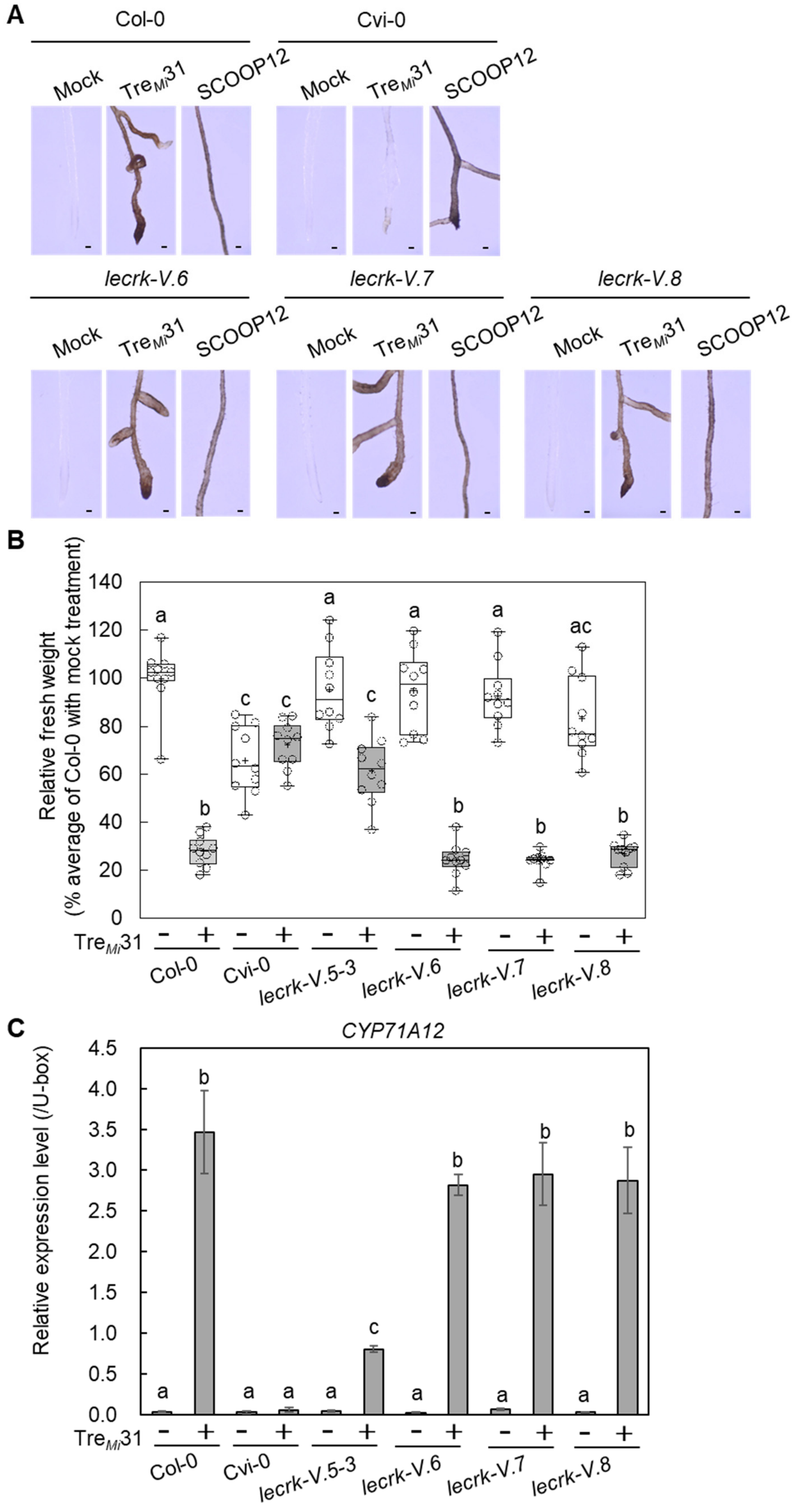

A
Col-0
Cvi-0
Mock
Tre$_{Mi}$31
SCOOP12
lecrk-V.6
lecrk-V.7
lecrk-V.8
B
Relative fresh weight
(% average of Col-0 with mock treatment)
Tre$_{Mi}$31
Col-0
Cvi-0
lecrk-V.5-3
lecrk-V.6
lecrk-V.7
lecrk-V.8
C
CYP71A12
Relative expression level (/U-box)

Fig. S21. T-DNA insertions in *LecRK-V.6*, *LecRK-V.7*, and *LecRK-V.8* do not affect Tre$_{Mi}$31-induced responses. (**A** to **C**) *lecrk-V.6*, *lecrk-V.7*, and *lecrk-V.8* mutants exhibit root pigmentation (**A**), growth inhibition (**B**), and *CYP71A12* expression (**C**) in response to 50 µM Tre$_{Mi}$31, similar to Col-0. In (**A**), root pigmentation was analysed by using four seedlings per treatment. The black bars in (**A**) represent 100 µm. In the box plot (**B**), the 25th-75th percentiles are shown, with the median indicated by a central line, the mean by a black cross, and whiskers representing the full range. Each open circle represents one data point from 10 samples (with one seedling per sample). Different letters denote significant differences ($P \leq 0.05$, one-way ANOVA with Tukey's post hoc test). Experiments were repeated three times with consistent results. In the bar chart (**C**), transcript levels of *CYP71A12* in the seedlings upon treatment with 50 µM Tre$_{Mi}$31 for 6 hours were measured by RT-qPCR after normalization to the *U-box* housekeeping gene transcript (*AT5G15400*). Values are presented as mean ±SE of three biological replicates, with different letters indicating significant differences ($P \leq 0.05$, one-way ANOVA with Tukey's post hoc test).

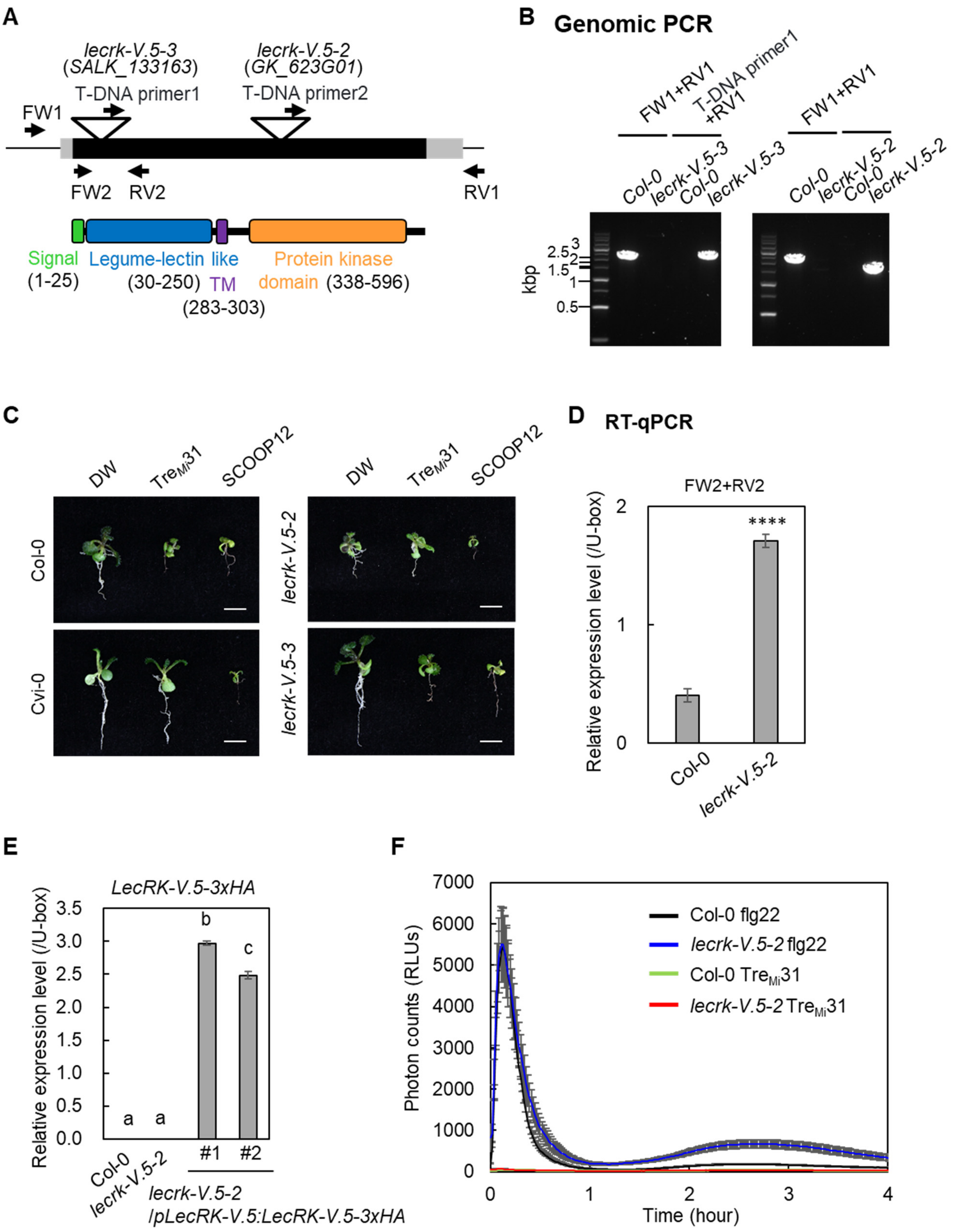
A
lecrk-V.5-3
(SALK_133163)
lecrk-V.5-2
(GK_623G01)
T-DNA primer1
T-DNA primer2
FW1
FW2
RV2
RV1
Signal
(1-25)
Legume-lectin like
(30-250)
TM
(283-303)
Protein kinase
domain
(338-596)
B
Genomic PCR
FW1+RV1
T-DNA primer1
+RV1
FW1+RV1
Col-0
lecrk-V.5-3
Col-0
lecrk-V.5-3
Col-0
lecrk-V.5-2
Col-0
lecrk-V.5-2
kbp
3
2.5
2
1.5
1
0.5
C
DW
Tre$_{Mi}$31
SCOOP12
DW
Tre$_{Mi}$31
SCOOP12
Col-0
Cvi-0
lecrk-V.5-2
lecrk-V.5-3
D
RT-qPCR
FW2+RV2
Relative expression level (/U-box)
2
1
0
****
Col-0
lecrk-V.5-2
E
LecRK-V.5-3xHA
Relative expression level (/U-box)
3.5
3.0
2.5
2.0
1.5
1.0
0.5
0.0
a
a
b
c
#1
#2
Col-0
lecrk-V.5-2
lecrk-V.5-2
/pLecRK-V.5:LecRK-V.5-3xHA
F
Photon counts (RLUs)
7000
6000
5000
4000
3000
2000
1000
0
Col-0 flg22
lecrk-V.5-2 flg22
Col-0 Tre$_{Mi}$31
lecrk-V.5-2 Tre$_{Mi}$31
0
1
2
3
4
Time (hour)

Fig. S22. *LecRK-V.5* is required for Tre$_{Mi}$31 signaling. (**A**) *Schematic representation of the LecRK-V.5 gene, showing the positions of T-DNA insertions and primers used for genotyping and RT-qPCR. The LecRK-V.5 gene has no introns, with exons and untranslated regions depicted in black and gray, respectively.* (**B**) *Genotyping of lecrk-V.5-2 and lecrk-V.5-3 mutants.* (**C**) *lecrk-V.5-3* mutants exhibit reduced seedling growth inhibition upon treatment with 50 µM Tre$_{Mi}$31 compared to Col-0, while *lecrk-V.5-2* mutants and Cvi-0 show no responses. Two µM SCOOP12 induces seedling growth inhibition in all genotypes. Four seedlings were analyzed per treatment. White bars represent 1 cm. (**D**) Transcript levels of truncated *LecRK-V.5* in *lecrk-V.5-2* mutants were measured by RT-qPCR after normalization to the *U-box* housekeeping gene transcript (*AT5G15400*). Values are presented as mean ± SE of three technical replicates, with asterisks indicating significant differences ($^{****}P \leq 0.0001$, Student's t-test). (**E**) Transcript levels of *LecRK-V.5-3xHA* in the complementation lines of *lecrk-V.5-2 /pLecRK-V.5:LecRK-V.5-3xHA* quantified by RT-qPCR, normalized to the *U-box* housekeeping gene transcript (*AT5G15400*). Values are presented as mean ± SE of three technical replicates, with different letters denoting significant differences ($P \leq 0.0001$, one-way ANOVA with Tukey's post hoc test). (**F**) Treatment with 50 µM Tre$_{Mi}$31 does not induce ROS production in Col-0. Flg22 was used as a positive control. The time course of ROS production was measured by a luminol-based assay, with results shown in relative luminescence units (RLUs). Experiments were repeated three times with consistent results.

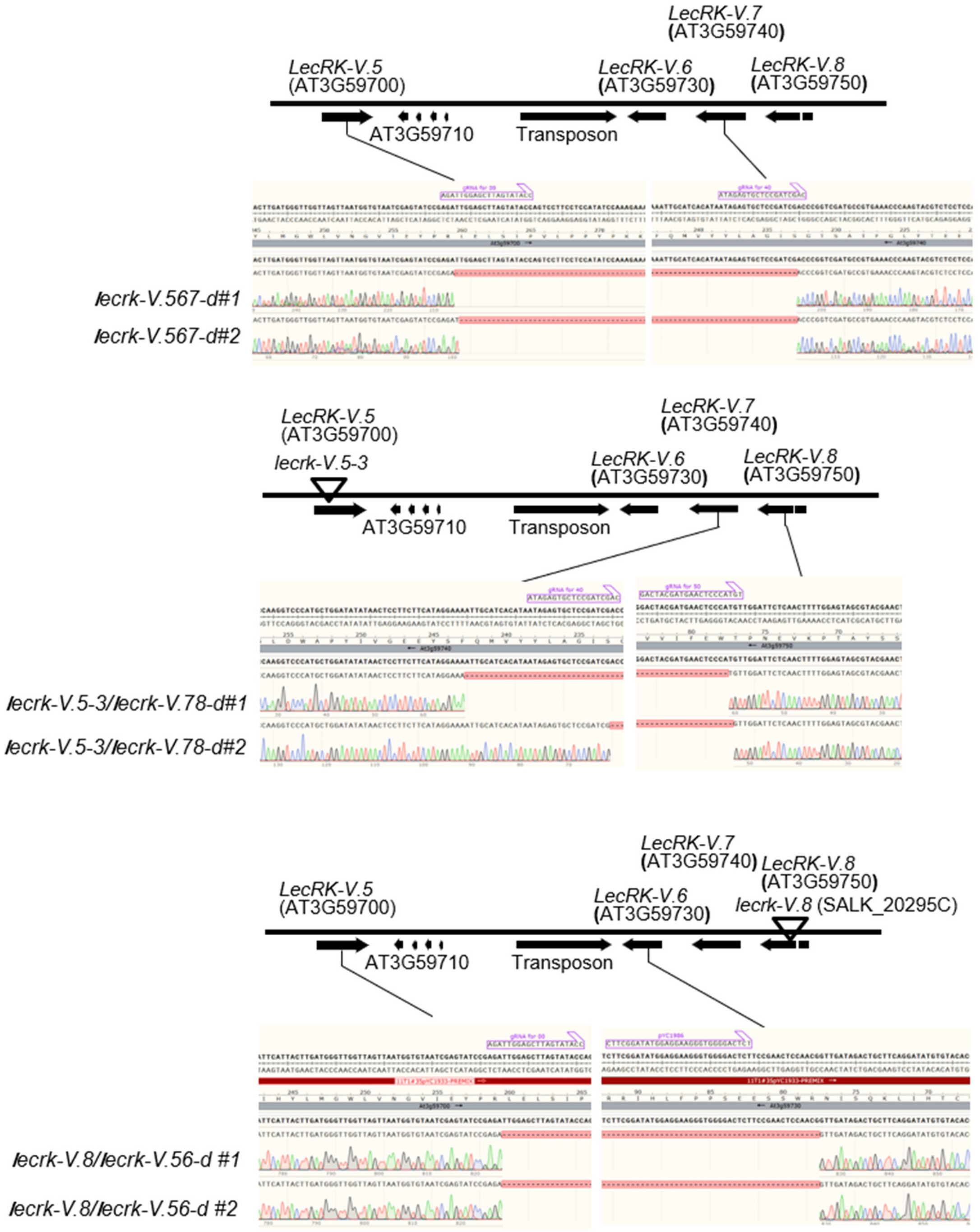

Fig. S23. Confirmation of deletion of genes in CRISPR lines, *lecrk-V.567-d*, *lecrk-V.5-3/lecrk-V.78-d*, and *lecrk-V.8/lecrk-V.56-d* lines by Sanger sequencing.

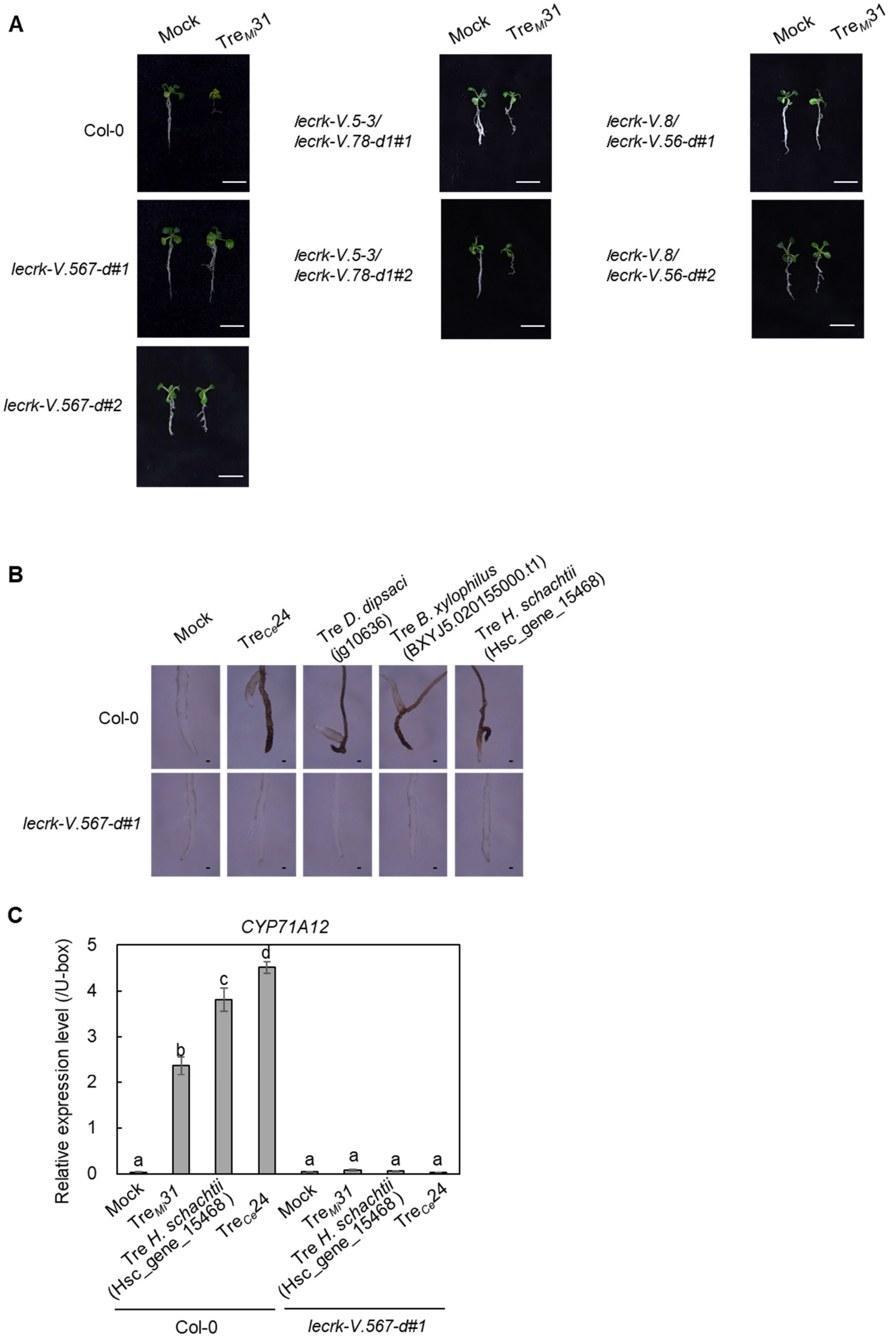
A
Mock
Tre$_{Mi}$31
Col-0
lecrk-V.567-d#1
lecrk-V.567-d#2
lecrk-V.5-3/ lecrk-V.78-d1#1
lecrk-V.5-3/ lecrk-V.78-d1#2
lecrk-V.8/ lecrk-V.56-d#1
lecrk-V.8/ lecrk-V.56-d#2
B
Mock
Tre$_{Ce}$24
Tre D. dipsaci (jg10636)
Tre B. xylophilus (BXYJ5.020155000.t1)
Tre H. schachtii (Hsc_gene_15468)
Col-0
lecrk-V.567-d#1
C
CYP71A12
Relative expression level (/U-box)
0
1
2
3
4
5
a
b
c
d
a
a
a
a
Mock
Tre$_{Mi}$31
Tre H. schachtii (Hsc_gene_15468)
Tre$_{Ce}$24
Col-0
lecrk-V.567-d#1

Fig. S24. *LecRK-V.5* and *LecRK-V.6* are involved in Tre$_{Mi}$31 recognition. (**A**) CRISPR deletion line *lecrk-V.5-3/lecrk-V.78-d* shows reduced seedling growth inhibition upon treatment with 50 µM Tre$_{Mi}$31, while *lecrk-V.567-d* and *lecrk-V.8/lecrk-V.56-d* lines do not induce the responses. White bars represent 1 cm. Four seedlings were analyzed per treatment. (**B**) *lecrk-V.567-d*#1 line does not induce root pigmentation upon treatment with 50 µM trehalose-derived peptides from PPNs. The peptides used are the same as those in Fig. S11A. Four seedlings were analyzed per treatment. Black bars represent 100 µm. (**C**) *lecrk-V.567-d*#1 line does not induce *CYP71A12* expression in response to 50 µM Tre$_{Ce}$24 and the trehalose-derived peptide from *H. schachtii* (Tre *H. schachtii* (Hsc_gene_15468)). Tre *H. schachtii* (Hsc_gene_15468) peptide used is the same as those in Fig. S11A. Transcript levels of *CYP71A12* in the seedlings upon treatment with 50 µM Tre$_{Ce}$24, Tre$_{Mi}$31, or Tre *H. schachtii* were measured by RT-qPCR after normalization to the *U-box* housekeeping gene transcript (*AT5G15400*). Values are presented as mean ± SE of three technical replicates, with different letters indicating significant differences ($P \leq 0.05$, one-way ANOVA with Tukey's post hoc test). Experiments were repeated three times with consistent results.

LecRK-V.5 *LecRK-V.6*

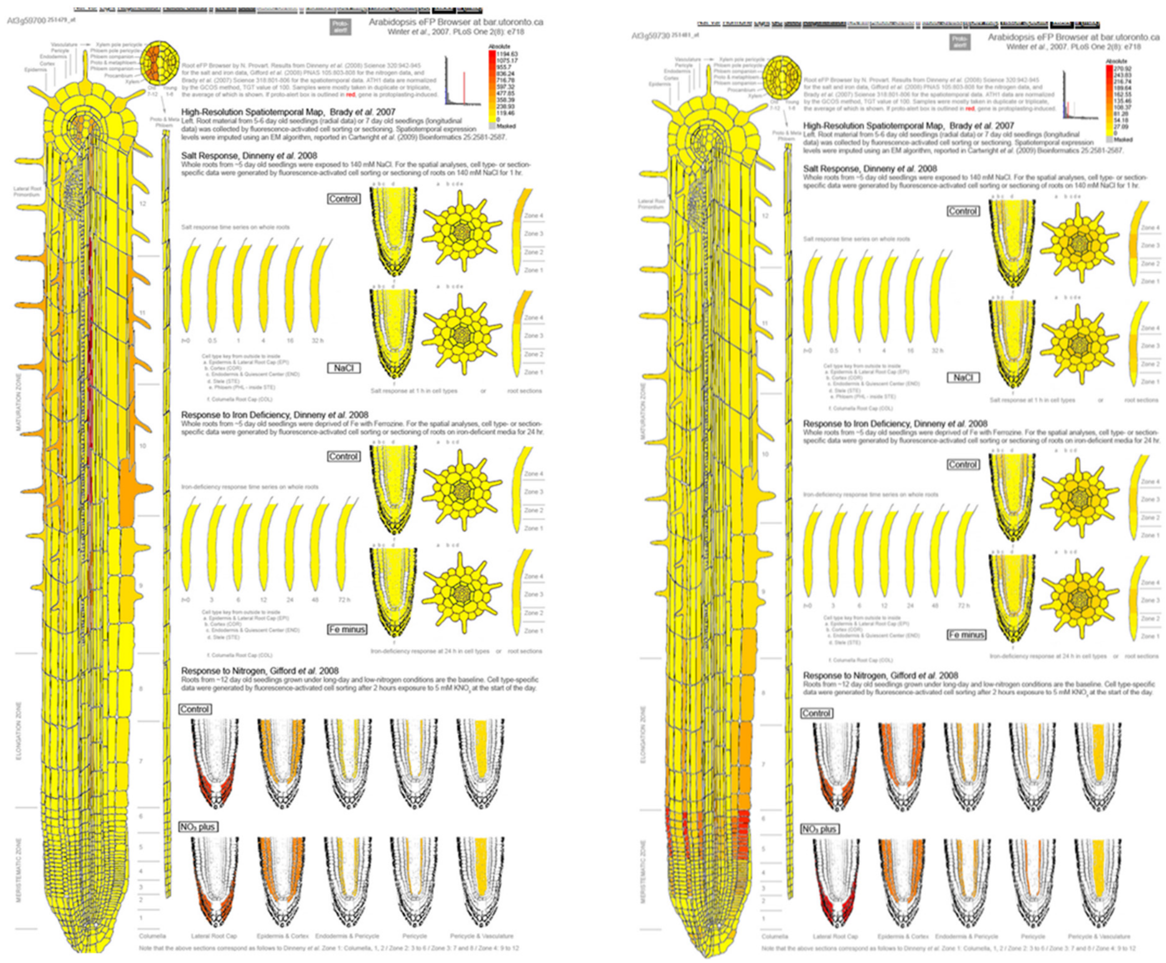

Fig. S25. Tissue-specific transcriptomic data of *LecRK-V.5* and *LecRK-V.6* from the eFP browser. *LecRK-V.5* and *LecRK-V.6* are expressed in most root tissues, with *LecRK-V.5* showing relatively high expression in procambial cells and *LecRK-V.6* in cortical cells. The expression value of *LecRK-V.5* and *LecRK-V.6* is also shown in Table S13.

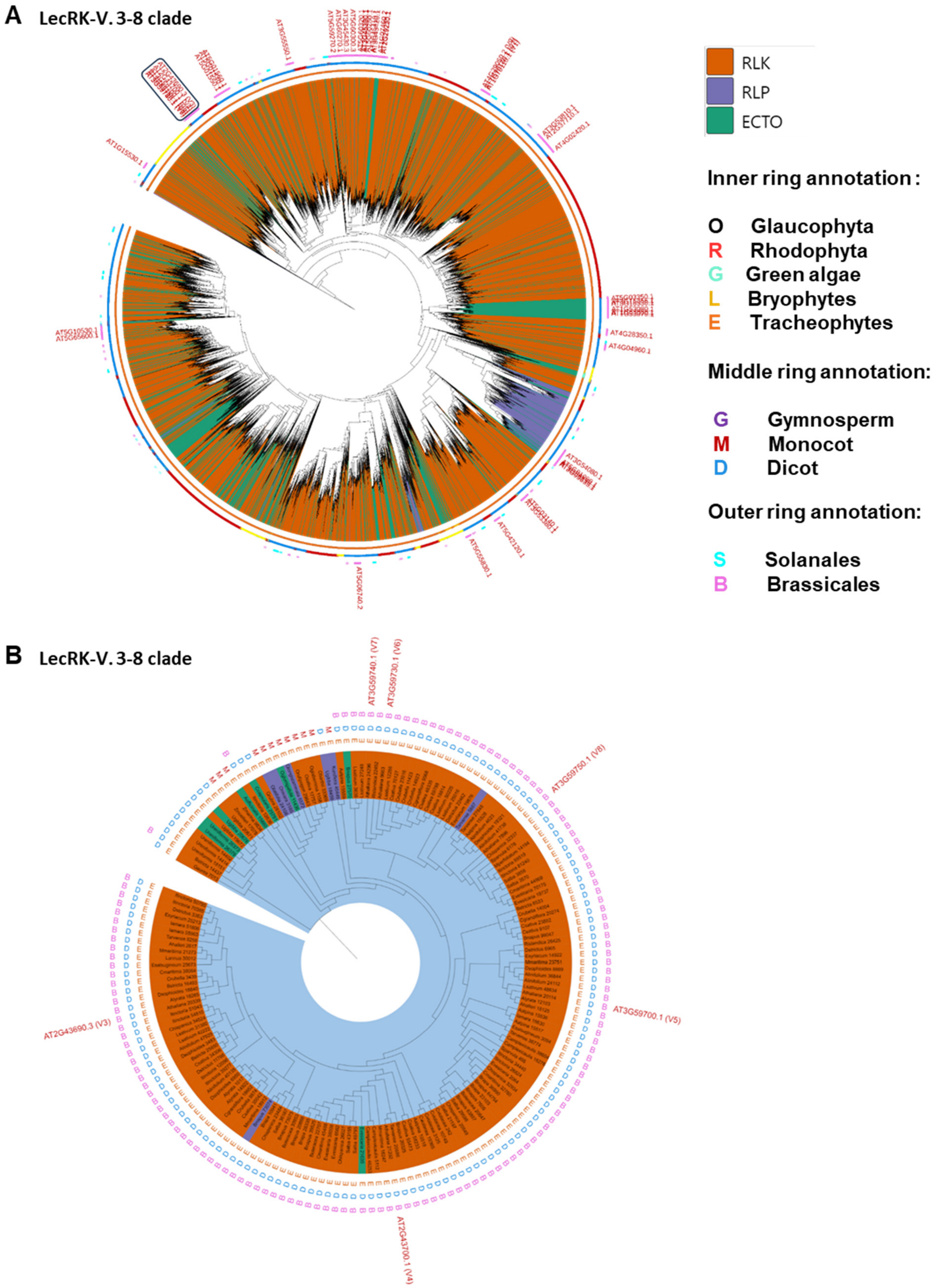
A LecRK-V. 3-8 clade
RLK
RLP
ECTO
Inner ring annotation :
O Glaucophyta
R Rhodophyta
G Green algae
L Bryophytes
E Tracheophytes
Middle ring annotation:
G Gymnosperm
M Monocot
D Dicot
Outer ring annotation:
S Solanales
B Brassicales
B LecRK-V. 3-8 clade
AT3G59740.1 (V7)
AT3G59730.1 (V6)
AT3G59750.1 (V8)
AT3G59700.1 (V5)
AT2G43700.1 (V4)
AT2G43690.3 (V3)

Fig. S26. Phylogenetic analysis reveals that *LecRK-V.5, LecRK-V.6, LecRK-V.7,* and *LecRK-V.8* cluster together and are conserved only in Brassicales. (**A**) Ectodomain phylogenetic tree of all L-lectins (14,465 members) across 350 species from Glaucophyta, red algae, green algae, Bryophytes, and Tracheophytes (*87, 88*), shows that *LecRK-V.3* to *LecRK-V.8* clusters within the same clade. (**B**) Ectodomain phylogenetic tree of *LecRK-V.3* to *LecRK-V.8.* Branches: Orange-RLK, Purple-RLP, Green-ectodomain-only proteins (lacking kinase and transmembrane regions). Inner ring: Black-Glaucophyta, Red-Rhodophyta, Light green-green algae, Light orange-bryophytes, and Orange-tracheophytes. Middle ring: Yellow-basal angiosperms, Red-monocots, Blue-dicots, and Purple-gymnosperms. Outer ring: Pink-Brassicales, Cyan-Solanales. *Arabidopsis* members are labeled.

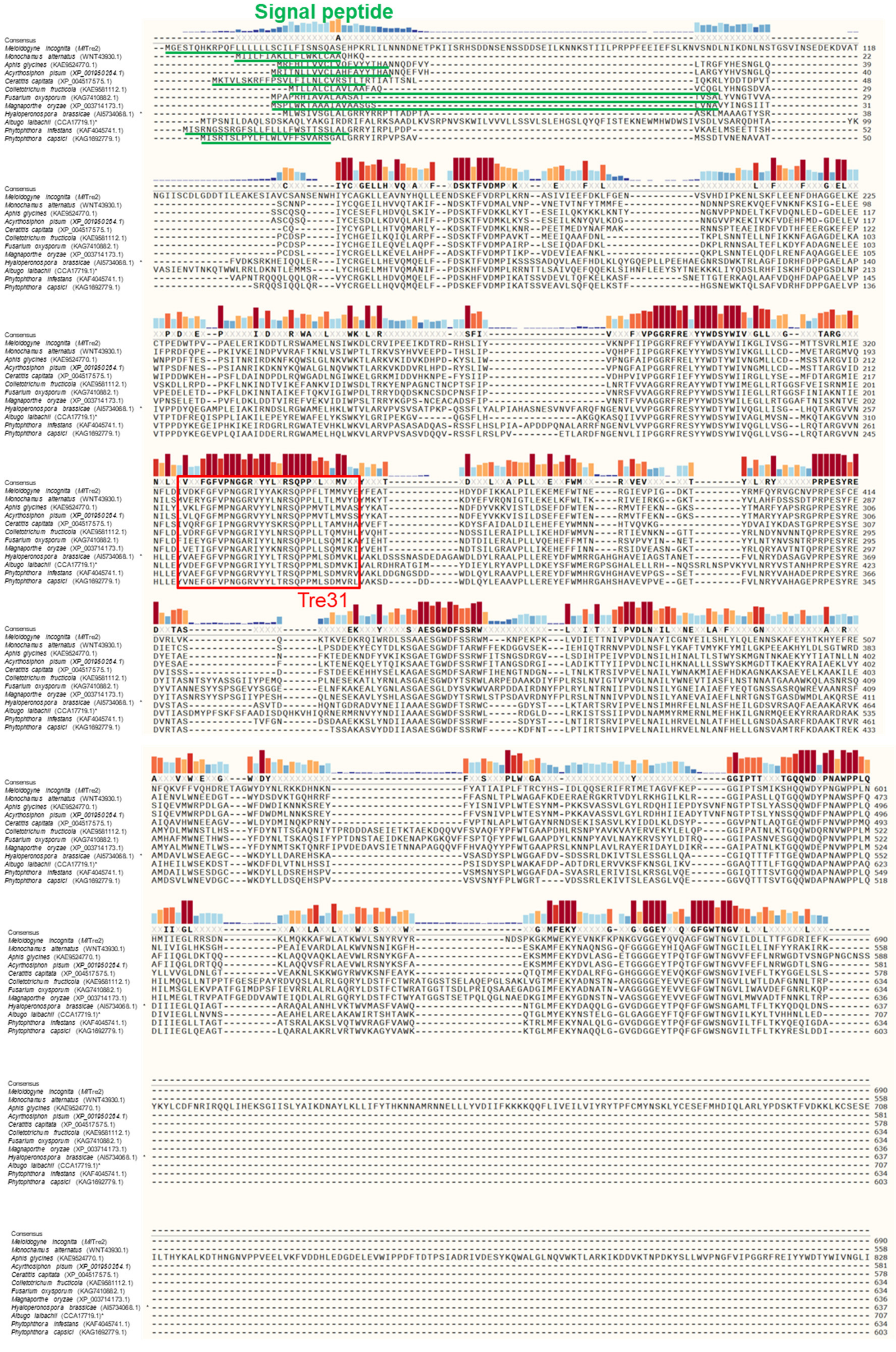
Signal peptide
Tre31
Consensus
Meloidogyne incognita (MiTre2)
Monochamus alternatus (WNT43930.1)
Aphis glycines (KAE9524770.1)
Acyrthosiphon pisum (XP_001950264.1)
Ceratitis capitata (XP_004517575.1)
Colletotrichum fructicola (KAE9581112.1)
Fusarium oxysporum (KAG7410882.1)
Magnaporthe oryzae (XP_003714173.1)
Hyaloperonospora brassicae (AI5734068.1) *
Albugo laibachii (CCA17719.1)*
Phytophthora infestans (KAF4045741.1)
Phytophthora capsici (KAG1692779.1)

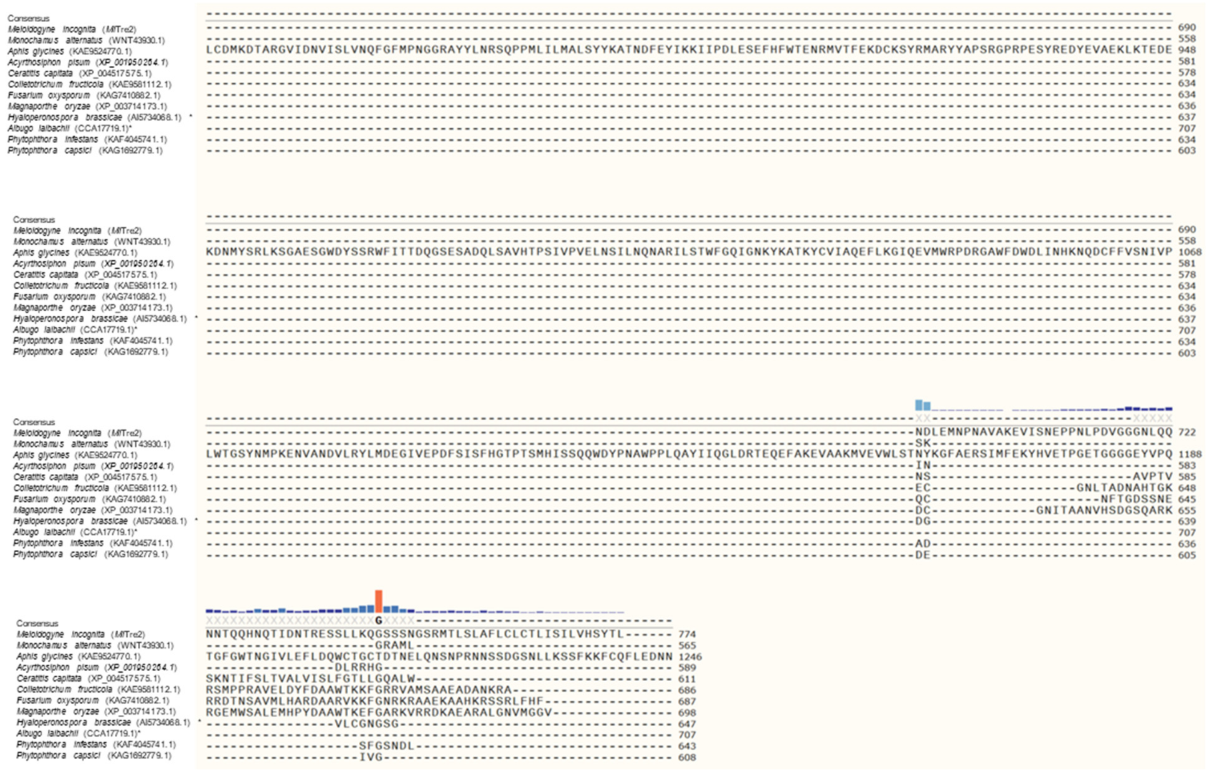

Fig. S27. Amino acid sequence alignment of secreted trehalase proteins of phytophagous insects and pathogenic fungi. Regions corresponding to predicted signal peptides by SignalP-6.0 are highlighted with green lines. The 31 amino acid residues of PPNs (Tre31) are shown in a red box. The degree of conservation of each residue among sequences is displayed as colored bars above the alignment.

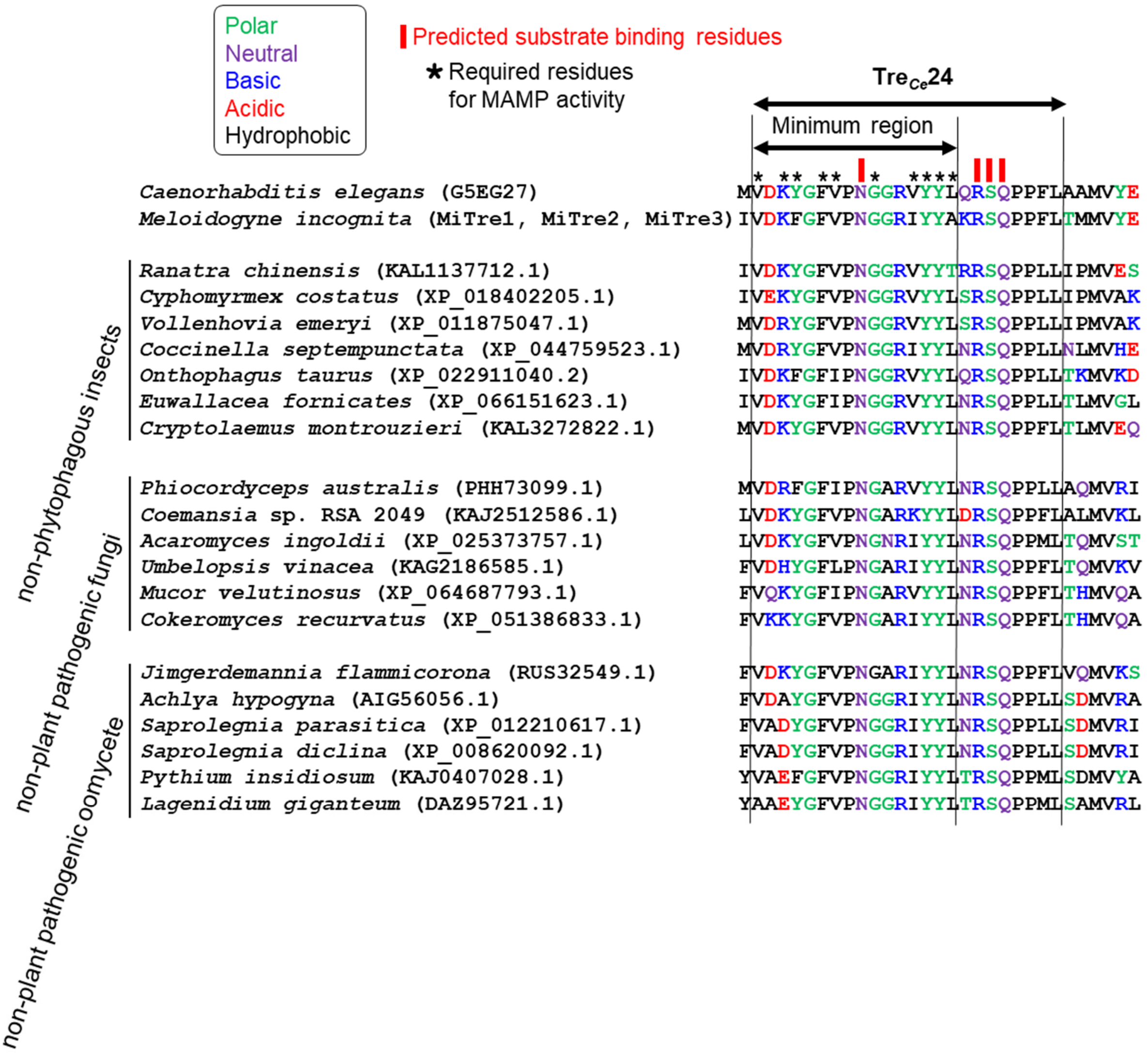

Fig. S28. Sequence alignment of trehalase peptides from non-phytophagous insects, non-plant-pathogenic fungi, and oomycetes. Red lines highlight predicted substrate-binding residues. Asterisks indicate essential residues for MAMP activity, as determined by the *CYP71A12* expression assay using Tre$_{Ce}$19 (fig. S9). Amino acids are color-coded according to their chemical properties.

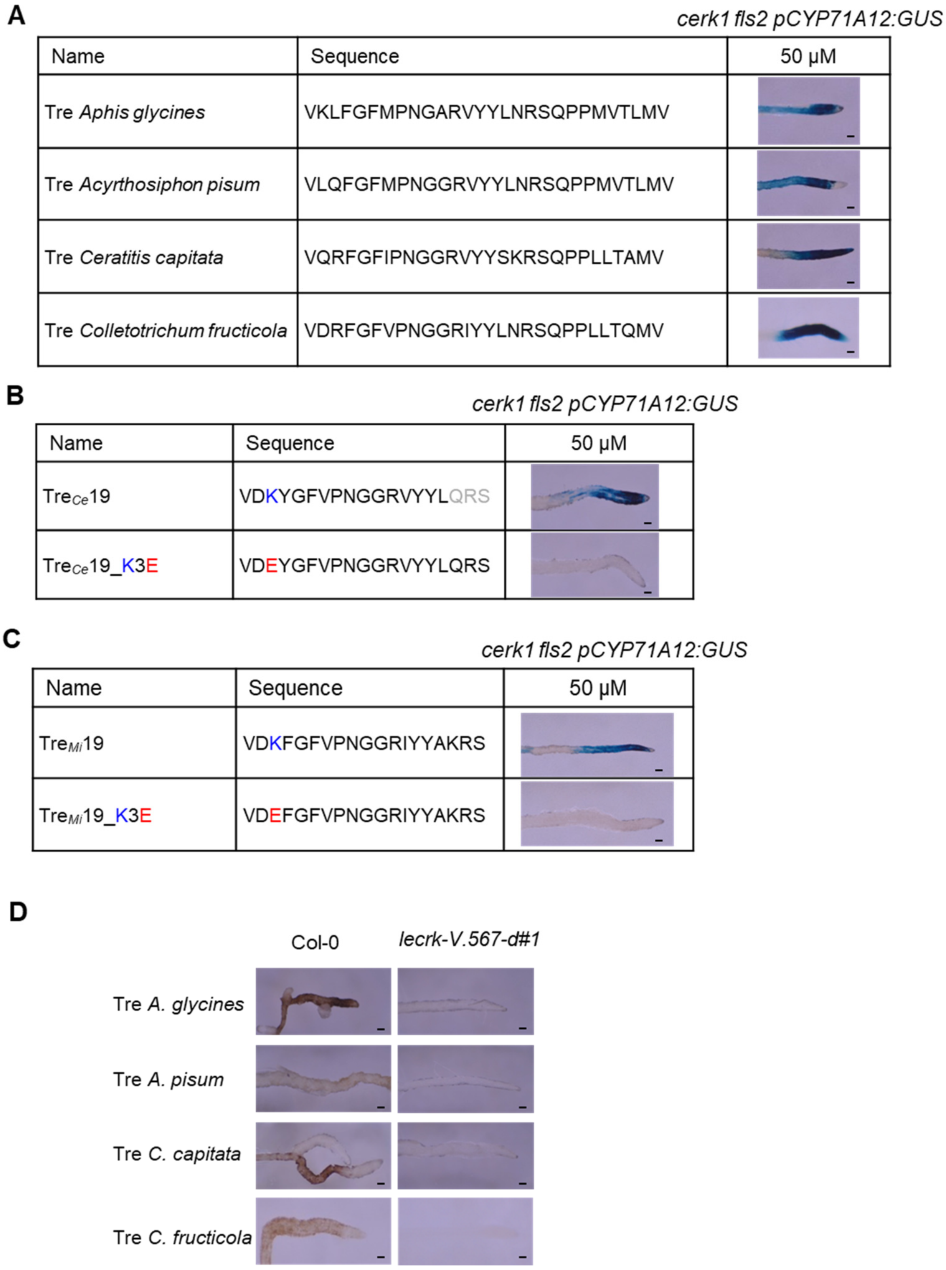

Fig. S29. Characterization of trehalase peptides from plant pathogenic fungi and phytophagous insects. (**A**) Trehalase peptides from phytophagous insects and plant pathogenic fungi induce *CYP71A12* expression in *cerk1 fls2 pCYP71A12:GUS* line. (**B** and **C**) Charge-reverse substitution at the third lysine residue to glutamic acid (K3E) in Tre$_{Ce}$19 and Tre$_{Mi}$19 results in loss of MAMP activity. (**D**) *lecrk-V.567-d*#1 line does not induce root pigmentation in response to 50 µM trehalase-derived peptides from phytophagous insects and plant pathogenic fungi. Black bars represent 100 µm. Four seedlings were analyzed per treatment. Experiments were repeated three times with consistent results.

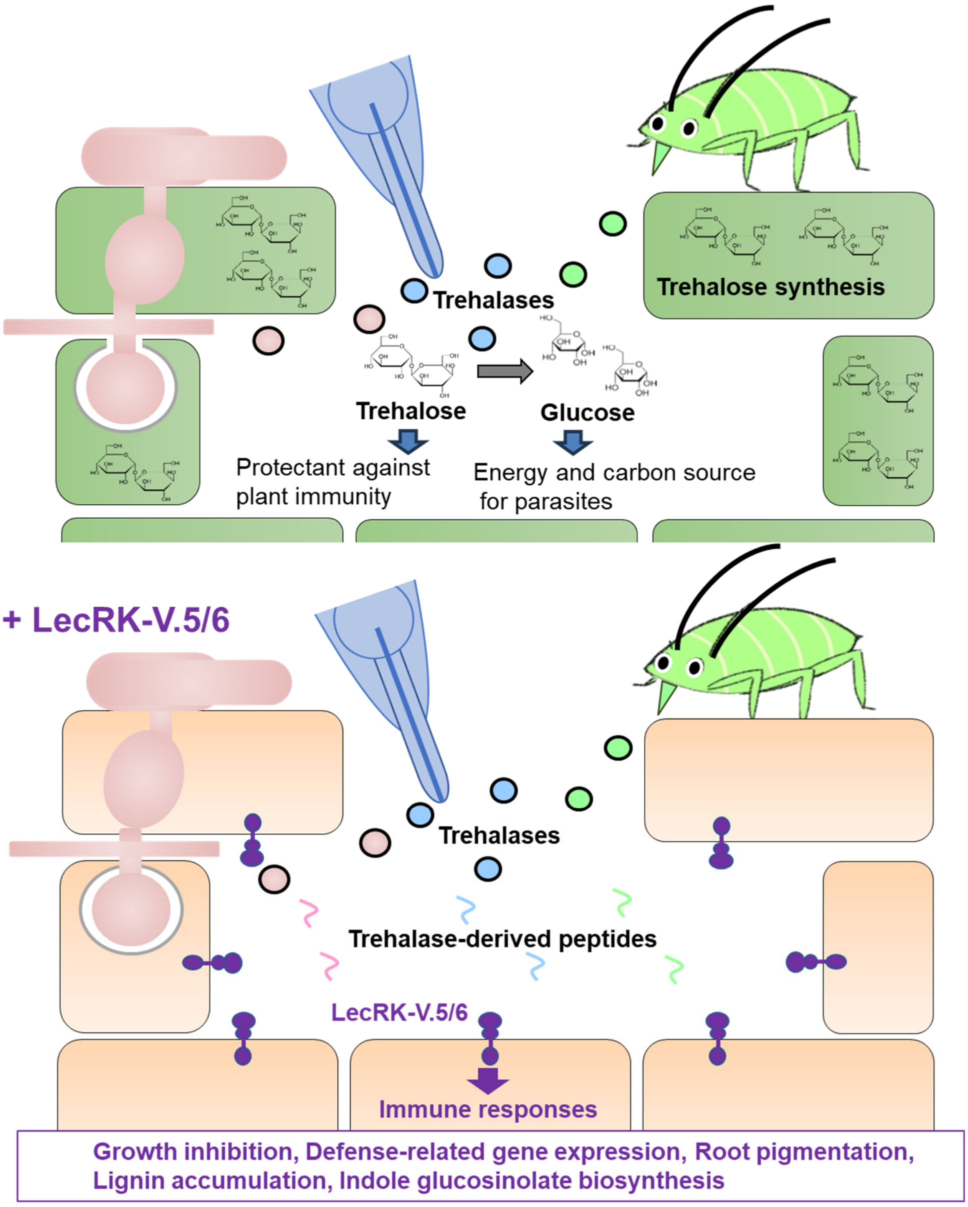

Fig. S30. Model of *Arabidopsis* Col-0 and PPN interaction through apoplastic trehalases and *LecRK-V.5* and *LecRK-V.6*. Trehalase proteins are produced during the migratory stage of some PPNs and are likely secreted into the apoplast, where they catalyze the conversion of trehalose into glucose. Similarly, trehalase proteins may also be secreted by certain fungal pathogens and phytophagous insects (*91*). Trehalose serves as an energy and carbon source for parasites and acts as a protectant against host immune defenses, while trehalase may facilitate glucose absorption by these organisms. In the Col-0 accession of *Arabidopsis*, these secreted trehalases or their peptide fragments are recognized, likely through LecRK-V.5 and LecRK-V.6. This recognition of trehalase-derived peptides activates immune responses, including growth inhibition, defense-related gene expression, root pigmentation, lignin accumulation, and indole glucosinolate biosynthesis.

Other Supplementary Materials for this manuscript include the following:

Table S1. Genes upregulated or downregulated after treatment with *C. elegans* extract compared to mock treatment in Col-0.

Table S2. Genes upregulated or downregulated after treatment with *M. incognita* extract compared to mock treatment in Col-0.

Table S3. Gene Ontology (GO) enrichment analysis of genes upregulated or downregulated after treatment with *M. incognita* or *C. elegans* extract.

Table S4. List of genes and isoforms with differential expression after treatment with *M. incognita* extract, *C. elegans* extract, $Tre_{Mi}31$, flg22, and chitin.

Table S5. List of MAMP candidate proteins identified by chromatography purification and LC-MS/MS analyses.

Table S6. List of identified peptides of *Ce*TRE3 by LC-MS/MS analyses.

Table S7. Genes upregulated or downregulated after treatment with $Tre_{Mi}31$.

Table S8. Gene Ontology (GO) enrichment analysis of genes upregulated or downregulated after treatment with $Tre_{Mi}31$.

Table S9. Root pigmentation phenotypes of Recombinant inbred lines (RILs).

Table S10. SNPs analyses of RILs by Sanger sequencing.

Table S11. Phenotypes of T-DNA insertion mutants of the genes in the narrowed-down region.

Table S12. Genes upregulated or downregulated after treatment with $Tre_{Mi}31$ in Col-0 and *lecrk V.5-3* mutant.

Table S13. Tissue-specific expression values of *LecRK-V.5* and *LecRK-V.6* obtained from the eFP browser.

Table S14. RNA-seq dataset used for transcriptome analysis and refined models of *Mi*Tre genes.

Table S15. Primers that were used in this paper.

REFERENCES AND NOTES

1. J. T. Jones, A. Haegeman, E. G. Danchin, H. S. Gaur, J. Helder, M. G. Jones, T. Kikuchi, R. Manzanilla-Lopez, J. E. Palomares-Rius, W. M. Wesemael, R. N. Perry, Top 10 plant-parasitic nematodes in molecular plant pathology. *Mol. Plant Pathol.* **14**, 946–961 (2013).

2. M. R. Khan, in *Novel Biological and Biotechnological Applications in Plant Nematode Management,* M. R. Khan, Ed. (Springer Nature Singapore, Singapore, 2023), pp. 3–45.

3. B. Molloy, T. Baum, S. Eves-van den Akker, Unlocking the development- and physiology-altering 'effector toolbox' of plant-parasitic nematodes. *Trends Parasitol.* **39**, 732–738 (2023).

4. S. Siddique, F. M. Grundler, Parasitic nematodes manipulate plant development to establish feeding sites. *Curr. Opin. Microbiol.* **46**, 102–108 (2018).

5. D. Couto, C. Zipfel, Regulation of pattern recognition receptor signalling in plants. *Nat. Rev. Immunol.* **16**, 537–552 (2016).

6. G. Felix, J. D. Duran, S. Volko, T. Boller, Plants have a sensitive perception system for the most conserved domain of bacterial flagellin. *Plant J.* **18**, 265–276 (1999).

7. L. Gómez-Gómez, T. Boller, FLS2: An LRR receptor-like kinase involved in the perception of the bacterial elicitor flagellin in Arabidopsis. *Mol. Cell* **5**, 1003–1011 (2000).

8. G. Kunze, C. Zipfel, S. Robatzek, K. Niehaus, T. Boller, G. Felix, The N terminus of bacterial elongation factor Tu elicits innate immunity in Arabidopsis plants. *Plant Cell* **16**, 3496–3507 (2004).

9. C. Zipfel, G. Kunze, D. Chinchilla, A. Caniard, J. D. Jones, T. Boller, G. Felix, Perception of the bacterial PAMP EF-Tu by the receptor EFR restricts Agrobacterium-mediated transformation. *Cell* **125**, 749–760 (2006).

10. A. Yamada, N. Shibuya, O. Kodama, T. Akatsuka, Induction of phytoalexin formation in suspension-cultured rice cells by *N*-acetyl-chitooligosaccharides. *Biosci. Biotechnol. Biochem.* **57**, 405–409 (1993).

11. A. Miya, P. Albert, T. Shinya, Y. Desaki, K. Ichimura, K. Shirasu, Y. Narusaka, N. Kawakami, H. Kaku, N. Shibuya, CERK1, a LysM receptor kinase, is essential for chitin elicitor signaling in Arabidopsis. *Proc. Natl. Acad. Sci. U.S.A.* **104**, 19613–19618 (2007).

12. K. Thor, S. Jiang, E. Michard, J. George, S. Scherzer, S. Huang, J. Dindas, P. Derbyshire, N. Leitao, T. A. DeFalco, P. Koster, K. Hunter, S. Kimura, J. Gronnier, L. Stransfeld, Y. Kadota, C. A. Bucherl, M. Charpentier, M. Wrzaczek, D. MacLean, G. E. D. Oldroyd, F. L. H. Menke, M. R. G. Roelfsema, R. Hedrich, J. Feijo, C. Zipfel, The calcium-permeable channel OSCA1.3 regulates plant stomatal immunity. *Nature* **585**, 569–573 (2020).

13. W. Tian, C. C. Hou, Z. J. Ren, C. Wang, F. G. Zhao, D. Dahlbeck, S. P. Hu, L. Y. Zhang, Q. Niu, L. G. Li, B. J. Staskawicz, S. Luan, A calmodulin-gated calcium channel links pathogen patterns to plant immunity. *Nature* **572**, 131–135 (2019).

14. Y. Kadota, J. Sklenar, P. Derbyshire, L. Stransfeld, S. Asai, V. Ntoukakis, J. D. Jones, K. Shirasu, F. Menke, A. Jones, C. Zipfel, Direct regulation of the NADPH oxidase RBOHD by the PRR-associated kinase BIK1 during plant immunity. *Mol. Cell* **54**, 43–55 (2014).

15. L. Li, M. Li, L. Yu, Z. Zhou, X. Liang, Z. Liu, G. Cai, L. Gao, X. Zhang, Y. Wang, S. Chen, J.-M. Zhou, The FLS2-associated kinase BIK1 directly phosphorylates the NADPH oxidase RbohD to control plant immunity. *Cell Host Microbe* **15**, 329–338 (2014).

16. A. P. Macho, C. Zipfel, Plant PRRs and the activation of innate immune signaling. *Mol. Cell* **54**, 263–272 (2014).

17. K. Sato, Y. Kadota, K. Shirasu, Plant immune responses to parasitic nematodes. *Front. Plant Sci.* **10**, 1165 (2019).

18. P. Manosalva, M. Manohar, S. H. von Reuss, S. Chen, A. Koch, F. Kaplan, A. Choe, R. J. Micikas, X. Wang, K. H. Kogel, P. W. Sternberg, V. M. Williamson, F. C. Schroeder, D. F. Klessig, Conserved nematode signalling molecules elicit plant defenses and pathogen resistance. *Nat. Commun.* **6**, 7795 (2015).

19. B. Mendy, M. W. Wang'ombe, Z. S. Radakovic, J. Holbein, M. Ilyas, D. Chopra, N. Holton, C. Zipfel, F. M. Grundler, S. Siddique, Arabidopsis leucine-rich repeat receptor-like kinase NILR1 is required for induction of innate immunity to parasitic nematodes. *PLOS Pathog.* **13**, e1006284 (2017).

20. L. Huang, Y. Yuan, C. Lewis, J. Kud, J. C. Kuhl, A. Caplan, L. M. Dandurand, I. Zasada, F. Xiao, NILR1 perceives a nematode ascaroside triggering immune signaling and resistance. *Curr. Biol.* **33**, 3992–3997.e3 (2023).

21. L. Huang, Y. Yuan, C. Ramirez, Z. Zhao, L. Chen, T. Griebel, J. Kud, J. C. Kuhl, A. Caplan, L.-M. Dandurand, F. Xiao, A receptor for dual ligands governs plant immunity and hormone response and is targeted by a nematode effector. *Proc. Natl. Acad. Sci. U.S.A.* **121**, e2412016121 (2024).

22. Y. A. Millet, C. H. Danna, N. K. Clay, W. Songnuan, M. D. Simon, D. Werck-Reichhart, F. M. Ausubel, Innate immune responses activated in Arabidopsis roots by microbe-associated molecular patterns. *Plant Cell* **22**, 973–990 (2010).

23. M. A. Teixeira, L. Wei, I. Kaloshian, Root-knot nematodes induce pattern-triggered immunity in Arabidopsis thaliana roots. *New Phytol.* **211**, 276–287 (2016).

24. K. Sato, T. Uehara, J. Holbein, Y. Sasaki-Sekimoto, P. Gan, T. Bino, K. Yamaguchi, Y. Ichihashi, N. Maki, S. Shigenobu, H. Ohta, R. B. Franke, S. Siddique, F. M. W. Grundler, T. Suzuki, Y. Kadota, K. Shirasu, Transcriptomic analysis of resistant and susceptible responses in a new model root-knot nematode infection system using *Solanum torvum* and *Meloidogyne arenaria*. *Front. Plant Sci.* **12**, 680151 (2021).

25. M. Safaeizadeh, T. Boller, C. Becker, Comparative RNA-seq analysis of Arabidopsis thaliana response to AtPep1 and flg22, reveals the identification of PP2-B13 and ACLP1 as new members in pattern-triggered immunity. *PLOS ONE* **19**, e0297124 (2024).

26. J. Wan, X. C. Zhang, D. Neece, K. M. Ramonell, S. Clough, S. Y. Kim, M. G. Stacey, G. Stacey, A LysM receptor-like kinase plays a critical role in chitin signaling and fungal resistance in Arabidopsis. *Plant Cell* **20**, 471–481 (2008).

27. M. Alblova, A. Smidova, V. Docekal, J. Vesely, P. Herman, V. Obsilova, T. Obsil, Molecular basis of the 14-3-3 protein-dependent activation of yeast neutral trehalase Nth1. *Proc. Natl. Acad. Sci. U.S.A.* **114**, E9811–E9820 (2017).

28. R. Shinya, H. Morisaka, T. Kikuchi, Y. Takeuchi, M. Ueda, K. Futai, Secretome analysis of the pine wood nematode *Bursaphelenchus xylophilus* reveals the tangled roots of parasitism and its potential for molecular mimicry. *PLOS ONE* **8**, e67377 (2013).

29. H. Silva, S. I. Anjo, B. Manadas, I. Abrantes, L. Fonseca, J. M. S. Cardoso, Comparative analysis of *Bursaphelenchus xylophilus* secretome under *Pinus pinaster* and *P. pinea* stimuli. *Front. Plant Sci.* **12**, 668064 (2021).

30. S. Siddique, Z. S. Radakovic, C. Hiltl, C. Pellegrin, T. J. Baum, H. Beasley, A. F. Bent, O. Chitambo, D. Chopra, E. G. J. Danchin, E. Grenier, S. S. Habash, M. S. Hasan, J. Helder, T. Hewezi, J. Holbein, M. Holterman, S. Janakowski, G. D. Koutsovoulos, O. P. Kranse, J. L. Lozano-Torres, T. R. Maier, R. E. Masonbrink, B. Mendy, E. Riemer, M. Sobczak, U. Sonawala, M. G. Sterken, P. Thorpe, J. J. M. van Steenbrugge, N. Zahid, F. Grundler, S. Eves-van den Akker, The genome and lifestage-specific transcriptomes of a plant-parasitic nematode and its host reveal susceptibility genes involved in trans-kingdom synthesis of vitamin B5. *Nat. Commun.* **13**, 6190 (2022).

31. B. Molloy, D. S. Shin, J. Long, C. Pellegrin, B. Senatori, P. Vieira, P. J. Thorpe, A. Damm, M. Ahmad, K. Vermeulen, L. Derevnina, S. Wei, A. Sperling, E. Reyes Estevez, S. Bruty, V. H. M. de Souza, O. P. Kranse, T. Maier, T. Baum, S. Eves-van den Akker, The origin, deployment, and evolution of a plant-parasitic nematode effectorome. *PLOS Pathog.* **20**, e1012395 (2024).

32. C. Pellegrin, A. Damm, A. L. Sperling, B. Molloy, D. S. Shin, J. Long, P. Brett, T. C. Iguh, O. P. Kranse, A. D. Bravo, S. J. Lynch, B. Senatori, P. Vieira, J. Mejias, A. Kumar, R. E. Masonbrink, T. R. Maier, T. J. Baum, S. Eves-van den Akker, The SUbventral-Gland Regulator (SUGR-1) of nematode virulence. *Proc. Natl. Acad. Sci. U.S.A.* **122**, e2415861122 (2025).

33. M. Da Rocha, C. Bournaud, J. Dazeniere, P. Thorpe, M. Bailly-Bechet, C. Pellegrin, A. Pere, P. Grynberg, L. Perfus-Barbeoch, S. Eves-van den Akker, E. G. J. Danchin, Genome expression dynamics

reveal the parasitism regulatory landscape of the root-knot nematode *Meloidogyne incognita* and a promoter motif associated with effector genes. *Genes* **12**, 771 (2021).

34. J. Abramson, J. Adler, J. Dunger, R. Evans, T. Green, A. Pritzel, O. Ronneberger, L. Willmore, A. J. Ballard, J. Bambrick, S. W. Bodenstein, D. A. Evans, C.-C. Hung, M. O'Neill, D. Reiman, K. Tunyasuvunakool, Z. Wu, A. Žemgulytė, E. Arvaniti, C. Beattie, O. Bertolli, A. Bridgland, A. Cherepanov, M. Congreve, A. I. Cowen-Rivers, A. Cowie, M. Figurnov, F. B. Fuchs, H. Gladman, R. Jain, Y. A. Khan, C. M. R. Low, K. Perlin, A. Potapenko, P. Savy, S. Singh, A. Stecula, A. Thillaisundaram, C. Tong, S. Yakneen, E. D. Zhong, M. Zielinski, A. Žídek, V. Bapst, P. Kohli, M. Jaderberg, D. Hassabis, J. M. Jumper, Accurate structure prediction of biomolecular interactions with AlphaFold 3. *Nature* **630**, 493–500 (2024).

35. M. C. Guillou, E. Vergne, S. Aligon, S. Pelletier, F. Simonneau, A. Rolland, S. Chabout, G. Mouille, K. Gully, P. Grappin, F. Montrichard, S. Aubourg, J. P. Renou, The peptide SCOOP12 acts on reactive oxygen species homeostasis to modulate cell division and elongation in Arabidopsis primary root. *J. Exp. Bot.* **73**, 6115–6132 (2022).

36. S. Hou, D. Liu, S. Huang, D. Luo, Z. Liu, Q. Xiang, P. Wang, R. Mu, Z. Han, S. Chen, J. Chai, L. Shan, P. He, The Arabidopsis MIK2 receptor elicits immunity by sensing a conserved signature from phytocytokines and microbes. *Nat. Commun.* **12**, 5494 (2021).

37. J. Rhodes, H. Yang, S. Moussu, F. Boutrot, J. Santiago, C. Zipfel, Perception of a divergent family of phytocytokines by the Arabidopsis receptor kinase MIK2. *Nat. Commun.* **12**, 705 (2021).

38. L. Gomez-Gomez, Z. Bauer, T. Boller, Both the extracellular leucine-rich repeat domain and the kinase activity of FSL2 are required for flagellin binding and signaling in Arabidopsis. *Plant Cell* **13**, 1155–1163 (2001).

39. M. Kanehisa, Y. Sato, M. Kawashima, KEGG mapping tools for uncovering hidden features in biological data. *Protein Sci.* **31**, 47–53 (2022).

40. J. Rajniak, B. Barco, N. K. Clay, E. S. Sattely, A new cyanogenic metabolite in Arabidopsis required for inducible pathogen defence. *Nature* **525**, 376–379 (2015).

41. B. Schwessinger, M. Roux, Y. Kadota, V. Ntoukakis, J. Sklenar, A. Jones, C. Zipfel, Phosphorylation-dependent differential regulation of plant growth, cell death, and innate immunity by the regulatory receptor-like kinase BAK1. *PLOS Genet.* **7**, e1002046 (2011).

42. M. Roux, B. Schwessinger, C. Albrecht, D. Chinchilla, A. Jones, N. Holton, F. G. Malinovsky, M. Tor, S. de Vries, C. Zipfel, The *Arabidopsis* leucine-rich repeat receptor-like kinases BAK1/SERK3 and BKK1/SERK4 are required for innate immunity to hemibiotrophic and biotrophic pathogens. *Plant Cell* **23**, 2440–2455 (2011).

43. M. Gao, X. Wang, D. Wang, F. Xu, X. Ding, Z. Zhang, D. Bi, Y. T. Cheng, S. Chen, X. Li, Y. Zhang, Regulation of cell death and innate immunity by two receptor-like kinases in Arabidopsis. *Cell Host Microbe* **6**, 34–44 (2009).

44. M. Simon, O. Loudet, S. Durand, A. Berard, D. Brunel, F.-X. Sennesal, M. Durand-Tardif, G. Pelletier, C. Camilleri, Quantitative trait loci mapping in five new large recombinant inbred line populations of *Arabidopsis thaliana* genotyped with consensus single-nucleotide polymorphism markers. *Genetics* **178**, 2253–2264 (2008).

45. Y. Wang, K. Bouwmeester, P. Beseh, W. Shan, F. Govers, Phenotypic analyses of Arabidopsis T-DNA insertion lines and expression profiling reveal that multiple L-type lectin receptor kinases are involved in plant immunity. *Mol. Plant Microbe Interact.* **27**, 1390–1402 (2014).

46. M. Desclos-Theveniau, D. Arnaud, T.-Y. Huang, G. J.-C. Lin, W. Y. Chen, Y.-C. Lin, L. Zimmerli, The Arabidopsis lectin receptor kinase LecRK-V.5 represses stomatal immunity induced by *Pseudomonas syringae* pv. *tomato* DC3000. *PLOS Pathog.* **8**, e1002513 (2012).

47. O. P. Kranse, I. Ko, R. Healey, U. Sonawala, S. Wei, B. Senatori, F. De Batte, J. Zhou, S. Eves-van den Akker, A low-cost and open-source solution to automate imaging and analysis of cyst nematode infection assays for *Arabidopsis thaliana*. *Plant Methods* **18**, 134 (2022).

48. D. Kuczynska-Wisnik, K. Stojowska-Swedrzynska, E. Laskowska, Intracellular protective functions and therapeutical potential of trehalose. *Molecules* **29**, 2088 (2024).

49. A. M. MacIntyre, J. X. Barth, M. C. Pellitteri Hahn, C. O. Scarlett, S. Genin, C. Allen, Trehalose synthesis contributes to osmotic stress tolerance and virulence of the bacterial wilt pathogen *Ralstonia solanacearum*. *Mol. Plant Microbe Interact.* **33**, 462–473 (2020).

50. S. Djonovic, J. M. Urbach, E. Drenkard, J. Bush, R. Feinbaum, J. L. Ausubel, D. Traficante, M. Risech, C. Kocks, M. A. Fischbach, G. P. Priebe, F. M. Ausubel, Trehalose biosynthesis promotes *Pseudomonas aeruginosa* pathogenicity in plants. *PLOS Pathog.* **9**, e1003217 (2013).

51. A. J. Foster, J. M. Jenkinson, N. J. Talbot, Trehalose synthesis and metabolism are required at different stages of plant infection by Magnaporthe grisea. *EMBO J.* **22**, 225–235 (2003).

52. X. Zhu, D. Fang, D. Li, J. Zhang, H. Jiang, L. Guo, Q. He, T. Zhang, A. P. Macho, E. Wang, Q. H. Shen, Y. Wang, J. M. Zhou, W. Ma, Y. Qiao, Phytophthora sojae boosts host trehalose accumulation to acquire carbon and initiate infection. *Nat. Microbiol.* **8**, 1561–1573 (2023).

53. M. Vanaporn, R. W. Titball, Trehalose and bacterial virulence. *Virulence* **11**, 1192–1202 (2020).

54. F. Baldacci-Cresp, M. Maucourt, C. Deborde, O. Pierre, A. Moing, R. Brouquisse, B. Favery, P. Frendo, Maturation of nematode-induced galls in Medicago truncatula is related to water status and primary metabolism modifications. *Plant Sci.* **232**, 77–85 (2015).

55. J. Hofmann, N. El Ashry Ael, S. Anwar, A. Erban, J. Kopka, F. Grundler, Metabolic profiling reveals local and systemic responses of host plants to nematode parasitism. *Plant J.* **62**, 1058–1071 (2010).

56. M. D. García, J. C. Argüelles, Trehalase inhibition by validamycin A may be a promising target to design new fungicides and insecticides. *Pest Manag. Sci.* **77**, 3832–3835 (2021).

57. V. Mani, C. S. Reddy, S. K. Lee, S. Park, H. R. Ko, D. G. Kim, B. S. Hahn, Chitin biosynthesis inhibition of *Meloidogyne incognita* by RNAi-mediated gene silencing increases resistance to transgenic tobacco plants. *Int. J. Mol. Sci.* **21**, 6626 (2020).

58. M. Van Ende, B. Timmermans, G. Vanreppelen, S. Siscar-Lewin, D. Fischer, S. Wijnants, C. L. Romero, S. Yazdani, O. Rogiers, L. Demuyser, G. Van Zeebroeck, Y. Cen, K. Kuchler, S. Brunke, P. Van

Dijck, The involvement of the Candida glabrata trehalase enzymes in stress resistance and gut colonization. *Virulence* **12**, 329–345 (2021).

59. Y. Pedreño, P. González-Párraga, M. Martínez-Esparza, R. Sentandreu, E. Valentín, J.-C. Argüelles, Disruption of the Candida albicans ATC1 gene encoding a cell-linked acid trehalase decreases hypha formation and infectivity without affecting resistance to oxidative stress. *Microbiology* **153** (Pt. 5), 1372–1381 (2007).

60. R. Sánchez-Fresneda, M. Martínez-Esparza, S. Maicas, J.-C. Argüelles, E. Valentín, In *Candida parapsilosis* the *ATC1* gene encodes for an acid trehalase involved in trehalose hydrolysis, stress resistance and virulence. *PLOS ONE* **9**, e99113 (2014).

61. R. G. Lopes, J. E. Muñoz, L. M. Barros, S. L. Alves-Jr, C. P. Taborda, B. U. Stambuk, The secreted acid trehalase encoded by the *CgATH1* gene is involved in *Candida glabrata* virulence. *Mem. Inst. Oswaldo Cruz* **115**, e200401 (2020).

62. J. Choi, K. Tanaka, Y. Cao, Y. Qi, J. Qiu, Y. Liang, S. Y. Lee, G. Stacey, Identification of a plant receptor for extracellular ATP. *Science* **343**, 290–294 (2014).

63. C. Wang, X. Huang, Q. Li, Y. Zhang, J. L. Li, Z. Mou, Extracellular pyridine nucleotides trigger plant systemic immunity through a lectin receptor kinase/BAK1 complex. *Nat. Commun.* **10**, 4810 (2019).

64. Q. Li, M. Zhou, F. Harris, Z. Mou, A group of L-type lectin receptor kinases function redundantly in mediating extracellular NAD(P) signaling in Arabidopsis. *Plant Physiol.* **195**, 2524–2527 (2024).

65. C. Gouhier-Darimont, E. Stahl, G. Glauser, P. Reymond, The *Arabidopsis* lectin receptor kinase LecRK-I.8 is involved in insect egg perception. *Front. Plant Sci.* **10**, 623 (2019).

66. R. Groux, E. Stahl, C. Gouhier-Darimont, E. Kerdaffrec, P. Jimenez-Sandoval, J. Santiago, P. Reymond, Arabidopsis natural variation in insect egg-induced cell death reveals a role for LECTIN RECEPTOR KINASE-I.1. *Plant Physiol.* **185**, 240–255 (2021).

67. S. Lacombe, A. Rougon-Cardoso, E. Sherwood, N. Peeters, D. Dahlbeck, H. P. van Esse, M. Smoker, G. Rallapalli, B. P. Thomma, B. Staskawicz, J. D. Jones, C. Zipfel, Interfamily transfer of a plant

pattern-recognition receptor confers broad-spectrum bacterial resistance. *Nat. Biotechnol.* **28**, 365–369 (2010).

68. A. P. Z. Mota, G. D. Koutsovoulos, L. Perfus-Barbeoch, E. Despot-Slade, K. Labadie, J. M. Aury, K. Robbe-Sermesant, M. Bailly-Bechet, C. Belser, A. Pere, C. Rancurel, D. K. Kozlowski, R. Hassanaly-Goulamhoussen, M. Da Rocha, B. Noel, N. Mestrovic, P. Wincker, E. G. J. Danchin, Unzipped genome assemblies of polyploid root-knot nematodes reveal unusual and clade-specific telomeric repeats. *Nat. Commun.* **15**, 773 (2024).

69. R. Blanc-Mathieu, L. Perfus-Barbeoch, J. M. Aury, M. Da Rocha, J. Gouzy, E. Sallet, C. Martin-Jimenez, M. Bailly-Bechet, P. Castagnone-Sereno, J. F. Flot, D. K. Kozlowski, J. Cazareth, A. Couloux, C. Da Silva, J. Guy, Y. J. Kim-Jo, C. Rancurel, T. Schiex, P. Abad, P. Wincker, E. G. J. Danchin, Hybridization and polyploidy enable genomic plasticity without sex in the most devastating plant-parasitic nematodes. *PLOS Genet.* **13**, e1006777 (2017).

70. D. Dai, C. Xie, Y. Zhou, D. Bo, S. Zhang, S. Mao, Y. Liao, S. Cui, Z. Zhu, X. Wang, F. Li, D. Peng, J. Zheng, M. Sun, Unzipped chromosome-level genomes reveal allopolyploid nematode origin pattern as unreduced gamete hybridization. *Nat. Commun.* **14**, 7156 (2023).

71. M. R. Winter, A. P. Taranto, H. Z. Yimer, A. Coomer Blundell, S. Siddique, V. M. Williamson, D. H. Lunt, Phased chromosome-scale genome assembly of an asexual, allopolyploid root-knot nematode reveals complex subgenomic structure. *PLOS ONE* **19**, e0302506 (2024).

72. I. Choi, P. Subramanian, D. Shim, B.-J. Oh, B.-S. Hahn, RNA-Seq of plant-parasitic nematode *Meloidogyne incognita* at various stages of its development. *Front. Genet.* **8**, 190 (2017).

73. S. Chen, Y. Zhou, Y. Chen, J. Gu, fastp: An ultra-fast all-in-one FASTQ preprocessor. *Bioinformatics* **34**, i884–i890 (2018).

74. A. Dobin, C. A. Davis, F. Schlesinger, J. Drenkow, C. Zaleski, S. Jha, P. Batut, M. Chaisson, T. R. Gingeras, STAR: Ultrafast universal RNA-seq aligner. *Bioinformatics* **29**, 15–21 (2013).

75. B. Li, C. N. Dewey, RSEM: Accurate transcript quantification from RNA-Seq data with or without a reference genome. *BMC Bioinformatics* **12**, 323 (2011).

76. B. T. Townsley, M. F. Covington, Y. Ichihashi, K. Zumstein, N. R. Sinha, BrAD-seq: Breath Adapter Directional sequencing: A streamlined, ultra-simple and fast library preparation protocol for strand specific mRNA library construction. *Front. Plant Sci.* **6**, 366 (2015).

77. B. Langmead, C. Trapnell, M. Pop, S. L. Salzberg, Ultrafast and memory-efficient alignment of short DNA sequences to the human genome. *Genome Biol.* **10**, R25 (2009).

78. T. Suzuki, T. Shinagawa, T. Niwa, H. Akeda, S. Hashimoto, H. Tanaka, Y. Hiroaki, F. Yamasaki, H. Mishima, T. Kawai, T. Higashiyama, K. Nakamura, The DROL1 subunit of U5 snRNP in the spliceosome is specifically required to splice AT-AC-type introns in Arabidopsis. *Plant J.* **109**, 633–648 (2022).

79. T. Suzuki, T. Kawai, S. Takemura, M. Nishiwaki, T. Suzuki, K. Nakamura, S. Ishiguro, T. Higashiyama, Development of the Mitsucal computer system to identify causal mutation with a high-throughput sequencer. *Plant Reprod.* **31**, 117–128 (2018).

80. T. Hulsen, J. de Vlieg, W. Alkema, BioVenn - a web application for the comparison and visualization of biological lists using area-proportional Venn diagrams. *BMC Genomics* **9**, 488 (2008).

81. S. Babicki, D. Arndt, A. Marcu, Y. Liang, J. R. Grant, A. Maciejewski, D. S. Wishart, Heatmapper: Web-enabled heat mapping for all. *Nucleic Acids Res.* **44**, W147–W153 (2016).

82. K. Kano, S. Noda, S. Sato, K. Kuwata, E. Mishiro-Sato, An efficient in-gel digestion method on small amounts of protein sample from large intact gel pieces. *Sep. Sci. Plus* **6**, 2200121 (2023).

83. Y. Kadota, T. W. H. Liebrand, Y. Goto, J. Sklenar, P. Derbyshire, F. L. H. Menke, M. A. Torres, A. Molina, C. Zipfel, G. Coaker, K. Shirasu, Quantitative phosphoproteomic analysis reveals common regulatory mechanisms between effector- and PAMP-triggered immunity in plants. *New Phytol.* **221**, 2160–2175 (2019).

84. H. Tsutsui, T. Higashiyama, pKAMA-ITACHI vectors for highly efficient CRISPR/Cas9-mediated gene knockout in Arabidopsis thaliana. *Plant Cell Physiol.* **58**, 46–56 (2017).

85. H. Nishiyama, B. T. Ngan, S. Nakagami, C. Ejima, T. Ishida, S. Sawa, Protocol for root-knot nematode culture by a hydroponic system and nematode inoculation to Arabidopsis. *Nematol. Res.* **45**, 45–49 (2015).

86. Y. Goto, N. Maki, Y. Ichihashi, D. Kitazawa, D. Igarashi, Y. Kadota, K. Shirasu, Exogenous treatment with glutamate induces immune responses in Arabidopsis. *Mol. Plant Microbe Interact.* **33**, 474–487 (2020).

87. B. P. M. Ngou, M. Wyler, M. W. Schmid, Y. Kadota, K. Shirasu, Evolutionary trajectory of pattern recognition receptors in plants. *Nat. Commun.* **15**, 308 (2024).

88. B. P. M. Ngou, R. Heal, M. Wyler, M. W. Schmid, J. D. G. Jones, Concerted expansion and contraction of immune receptor gene repertoires in plant genomes. *Nat. Plants* **8**, 1146–1152 (2022).

89. T. Obayashi, H. Hibara, Y. Kagaya, Y. Aoki, K. Kinoshita, ATTED-II v11: A plant gene coexpression database using a sample balancing technique by subagging of principal components. *Plant Cell Physiol.* **63**, 869–881 (2022).

90. J. T. Robinson, H. Thorvaldsdottir, D. Turner, J. P. Mesirov, igv.js: An embeddable JavaScript implementation of the Integrative Genomics Viewer (IGV). *Bioinformatics* **39**, (2023).

91. Y. Zhang, J. Fan, J. Sun, F. Francis, J. Chen, Transcriptome analysis of the salivary glands of the grain aphid, *Sitobion avenae*. *Sci. Rep.* **7**, 15911 (2017).
